# Deficiency of autism susceptibility gene Trio in cerebellar Purkinje cells leads to delayed motor impairments

**DOI:** 10.3389/fpsyt.2024.1396716

**Published:** 2025-04-10

**Authors:** Jinxin Wang, Yimeng Li, Dai Zhang, Wenzhi Sun, Jun Li

**Affiliations:** ^1^ State Key Laboratory of Cognitive Neuroscience and Learning and IDG/McGovern Institute for Brain Research, Beijing Normal University, Beijing, China; ^2^ Chinese Institute for Brain Research, Beijing, China; ^3^ NHC Key Laboratory of Mental Health, National Clinical Research Center for Mental Disorders, Peking University Sixth Hospital, Peking University Institute of Mental Health, Peking University, Beijing, China

**Keywords:** autism spectrum disorder, trio, motor dysfunctions, cerebellum, Purkinje cells

## Abstract

Autism spectrum disorder (ASD) is a group of neurodevelopmental disorders characterized by social interaction deficits, restricted interests and repetitive behaviors. The co-occurrence of motor impairments exacerbates the severity and societal impact of ASD, but the underlying mechanism remains to be elucidated. Research on the comorbidities of ASD including motor impairments could benefit in the life quality improvement in patients with ASD. Here we aimed at investigating the motor behaviors in mice with *Trio* deletion in Purkinje cells (PCs), and further exploring the cellular and molecular mechanisms. The protein level of Calbindin as PCs’ marker was determined. Behaviors including spontaneous locomotion activity, rotarod, beam balance and gait were tested in mice with the ages of 12-week and 20-week. Magnetic resonance imaging (MRI) scanning with T2 and DTI sequencing was performed in 12-week old mice. Although *Trio^fl/fl; Pcp2-Cre^* mice showed significant impairments of spontaneous locomotion activity in both 12-week and 20-week ages, only the 20-week but not 12-week *Trio^fl/fl; Pcp2-Cre^* mice showed extra mild abnormal motor, fine motor coordination, and gait. The decreased expression of Calbindin existed in both 12-week and 20-week old mice compared with control. Differentially expressed genes analysis from RNA-Seq and Gene Co-expression Network Analysis (GCNA) showed that Syne1 and its co-expressed genes were upregulated in *Trio^fl/fl; Pcp2-Cre^* mice compared to controls. In addition, abnormal ADC values suggested the long-term chronic damage in the cerebellum. Together, our findings indicate that the motor dysfunction in ASD are affected by *Trio* deletion in PCs with delayed in onset, accompanied with alterations in MRI, histological, and epigenetic level.

## 1 Introduction

Autism Spectrum Disorder (ASD) is a range of highly hereditary and heterogeneous neurodevelopmental disorders primarily diagnosed based on social impairment and repetitive behaviors. The prevalence of ASD has steadily increased in recent years, with current global estimates around 1% (1, 2). Over 70% of individuals with ASD also present with a wide range of comorbidities, including atypical cognitive impairments, anxiety and Attention Deficit Hyperactivity Disorder (ADHD). Motor dysfunction is also a common comorbidity of ASD (3), which exacerbates the core symptoms and augments the overall disorder burden.

Motor impairments have been observed in 50%-85% of individuals with ASD, with the main symptoms of deficits in visuomotor coordination and balance, muscle tone, and gait abnormalities (4, 5). Large cohort studies and systematic reviews suggested that these motor impairments potentially represent initial and primary features of atypical symptoms in autism (6). Mounting evidence pointed that cerebellar abnormalities play a critical role in ASD-related motor dysfunctions (7). Clinical research has identified cerebellar abnormalities in over 95% of individuals with ASD (8). Purkinje cells (PCs), as the only output neurons in the cerebellar cortex, are crucial in the generation and execution of motion (7). A significant reduction in PC number has been reported in ASD patients with autism (9), and dysfunction of PCs has also been shown to lead to abnormal motor behaviors in ASD animal models (10–12). However, the exact role of genetic factors in PC dysfunction and the resultant motor impairments in ASD remains unclear.

Trio protein serves as a pivotal guanine nucleotide exchange factor (GEF) in the ambit of cellular development, featuring an array of protein functional domains that orchestrate cellular proliferation, migration, and axonal evolution, notably inclusive of the GEF1 and GEF2 domains (13). Mutations within Trio, especially functional alterations in the GEF1 domain sequence, have been identified as related to the etiology of ASD and familial ataxia (14, 15). Concurrent clinical investigation revealed that patients with ASD harboring functional deficiency mutations in *TRIO* not only displays the conventional characteristics of ASD but also manifest developmental delays in gross motor or fine motor function (16). The loss of *Trio* function is associated with significant aberrations in cerebral morphology, evidenced by diminished cerebral mass, malformations within the hippocampal architecture, and disturbed cerebellar foliation processes in murine models (17). Given the cerebellum’s role in motor balance and emotional regulation, it may play a role in the motor impairment observed in ASD patients with *Trio* deficiency. *Trio* isoform, *Trio8/Solo*, manifests a unique expression profile within the PCs, critically modulating axonal maturation, with notably reduced expression in the cerebellum of murine models for Purkinje cell degeneration (PCD)-associated ataxia (18). Cerebellar research indicates that it is not yet entirely clear whether Trio knockout in PCs causes mice to develop motor behavioral phenotypes associated with ASD.

*De novo* mutations leading to functional loss of Trio are frequently reported in patient with ASD. However, the mechanisms underlying the concurrent occurrence of ASD and Trio deficiency-related impairments are not completely characterized. Hence, examining the postnatal role of Trio in cerebellar development and its potential in modulating ASD phenotypes is important. This study aims to investigate ASD-related motor dysfunctions in a conditional knockout (cKO) mouse model, in which *Trio* is deleted in PCs (*Trio^fl/fl;Pcp2-Cre^*). Our work provides an in-depth analysis of the PC abnormalities that contribute to motor deficits observed in ASD models.

## 2 Materials and methods

### 2.1 Animals

*Trio^fl/fl^* mice were purchased from Model Animal Research Center (Nanjing, China). *Trio^fl/fl^* mice were backcrossed with C57BL/6 mice for more than 10 generations. Ai14 mice (Express *td*Tomato fluorescence following Cre-mediated recombination, Jax007914) and *Pcp2-Cre* (express Cre recombinase under the control of the mouse PCs protein, Jax004146.) mice were supplied by the CIBR from Jackson lab. We generated *Trio^fl/fl;Pcp2-Cre^* mice by crossing *Trio^fl/fl^* mice with *Pcp2-Cre* mice. While *Trio^fl/fl; Pcp2-Cre^* ^;^ *Ai14 mice* was generated by *Trio^fl/+;^Ai14* and *Trio^fl/+;Pcp-2Cre^.* Animals were kept in standard environmental conditions with temperatures maintained between 22-25°C and a 12h light/12h dark cycle. All animal care and handling procedures were strictly in compliance with the ethical committee’s regulation for use and management of experimental animals at the CIBR (LARC-T019, CIBR-IACUC-023). The design and conduct of the experiments made every effort to adhere to the principles of animal welfare. At least 24 hours prior to behavioral experiments, mice were placed in the testing room to acclimate to the behavioral testing environment, which was designed to reflect their husbandry conditions. In addition, the mice were handled to become familiar with the experimenters. For MRI studies, mice were acclimated to the experimental environment 24 hours in advance. Following the completion of experiments, mice were euthanized by cervical dislocation under 1.25% tribromoethanol anesthesia or by perfusion with cold PBS for tissue collection.

### 2.2 Behavioral experiments

#### 2.2.1 Open field test

The open field test assessed spontaneous activity abilities of mice in an enclosure measuring 27.5 cm × 27.5 cm × 20 cm. Mice were placed at the center of the arena to start the experiment, with recording over a 2-hour period. Subsequent to each mouse’s trial, feces and urine were washed away as much as possible. 75% ethanol was used to remove any scents. Measured parameters included total distance moved, time spent in the center area, and the number of entries into the center zone.

#### 2.2.2 Rotarod test

The rotarod test was performed to evaluate the motor coordination of mice. Initially, mice were accustomed to the rotarod at a speed of 4 rpm for 1-minute. The speed was then accelerated from 4 rpm to 40 rpm over 180 seconds, and the velocity was maintained at 40 rpm for 120 seconds. The time until the mice fell off and the speed of the rotarod at the moment of falling were recorded. Each mouse underwent the test for three times, with intervals of 10 minutes between trials, over a consecutive 3-day period. If a mouse did not fall, its fall time was recorded as 300 seconds.

#### 2.2.3 Balance beam test

The balance beam test was employed to evaluate the locomotor balance and coordination of mice. The apparatus is consisted of beams positioned 50 cm above the ground with different sizes: a 1.5 cm square beam for the acclimation and training phase, and a 1 cm square beam and a 1 cm diameter round beam for the testing phase. Luminescent devices at the start and a dark box containing food and nesting materials at the end marked the course, which spanned three consecutive days, the first two for acclimation and training and the final day for testing. Mice traversed the same beam for three times per day, reaching the end box, with full crossing time recorded. On the testing day, the number of slip-offs by the hindlimbs was noted.

#### 2.2.4 Gait test

The Gait test (19) is an experiment employed to assess the accuracy of limb placement and the consistency of stride in the gait of mice. In this study, 12 and 20-week old male mice were made to traverse an open-top runway (50 cm long, 10 cm wide, with 10 cm high walls), with white paper laid at the bottom. The mice’s fore and hind paws were painted with two distinctly contrasting colors of non-toxic paint (for instance, in this experiment, red for the forepaws and green for the hind paws), allowing for the capture of footprints on paper during the run for subsequent analysis. The experiment gauged the following four parameters: Stride length: the average longitudinal distance between the left/right fore/hind paws, Hind-base width: the average lateral distance between the left and right hind paws, Front-base width: the average lateral distance between the left and right forepaws. Overlap between forepaw and hind paw placement: the vertical distance between the footprints of forelimbs and hind limbs on the same side. In a normal gait, the center of a hind paw in the subsequent step should align over the center of the forepaw from the preceding step, meaning the vertical distance between the overlapping footprints of the ipsilateral fore and hind paws should be very close to zero. For each parameter, three values from the central section of the recorded footprints were selected for measurement, and the average of these three values was taken as the parameter value for each mouse. As the mice employed in this experiment were genetically modified knockout mice, there is no theoretical presence of unilateral brain damage; hence, the data for the left and right paws were amalgamated for analysis.

### 2.3 Cerebellar measurement

To assess overall changes in the cerebellum of mice, we weighed the cerebella of knockout and control mice with similar body weights (25-30g). Using blunted dissection, intact cerebella were harvested, weighed, and the size was measured across two orthogonal axes. For further quantification of cerebellar size, we calculated the cross-sectional area of cerebellar slices taken along the vermis’s mid-sagittal plane. The mice were anesthetized and perfused with cold PBS and 4% paraformaldehyde. The brains were dehydrated with gradient sucrose, and the frozen sections were prepared with a thickness of 30μm, and photographed under fluorescence microscopy. Measure total cerebral area, molecular layer (ML), granular cell layer(GCL),and cerebral white matter(WM) area. Areas of all layers were computed using Imaris software. Also quantified was the thickness of the molecular layer across the first four lobules of the vermis in median sections. These data were derived from at least three pairs of mice, with a minimum of five slices per mouse.

### 2.4 Western blot

The mice were anesthetized with 5% sodium pentobarbital, and perfused with pre-cooling 4°C PBS. The cerebella were extracted and lysed with RIPA lysis buffer by using ultrasonic disintegration on ice. The protein concentrations were determined by using a BCA protein assay kit. Proteins were separated by electrophoresis in a gel using 80mV for 2 hours and transferred onto NC membranes at 220 mA for 1.5 hours. After blocking with 5% non-fat milk, the membranes were incubated with primary antibodies overnight at 4°C, followed by one hour with secondary antibodies at room temperature. The antibodies used in the experiment are listed below: Monoclonal Anti-Calbindin-D-28K antibody (C9848,1:20000, Millipore Sigma); Rabbit monoclonal to Syne-1(ab192234, 1:1000,abcam, Shanghai), Anti-GAPDH antibody (2118,1:2000, Cell Signaling Technology), Goat Anti-Mouse IgG H&L (HRP)(IH-0011, 1:2000, Dingguo), Goat Anti-Rabbit IgG H&L (HRP)(IH-0031, 1:2000, Dingguo). Images were captured using an ECL system within a gel imager. Data analysis included calculating the intensity of bands using Image J software.

### 2.5 Magnetic resonance imaging experiments

For MRI scans, the mice were fixed on an animal bed (Bruker, Ettlingen, Germany), with the head fixed using dental strips and dental pads. The mice were anesthetized by c1.25% tribromoethanol anesthesia intraperitoneal injection. The respiratory and heart rates as well as the body temperature of the mice were recorded to maintain a body temperature of 37.0 ± 0.5°C. Blood oxygenation was detected using a fiber-optic pulse oximetry sensor located on the foot of the mice. At the end of the MRI session, the mice were sacrificed under anesthesia by cervical dislocation.

### 2.6 Image acquisition

A Bruker Pharmascan 9.4 T imaging system (Bruker, Ettlingen,Germany) was used. In each animal, the following sequences were used: 1) two-dimensional Turbo Rapid Imaging with Refocused Echoes T2-weighted images (in three orthogonal directions) while positioning with the following scanning parameters: repetition time 2600 ms; echo time 33ms; field of view 18 × 15 mm^2^; pixel size 0.07 × 0.059 mm^2^) two-dimensional DTI scans using echo-planar imaging sequences with the scan parameters: repetition time 7,000 ms; echo time 33 ms; 22 coronal slices 0.7 mm; slice gap 0.07 mm; b-value 1,000 s/mm^2^; diffusion gradient pulse duration δ 4 ms. Left and right frequency codes were used to extend the diffusion sensitization gradient in 45 optimal directions with a flip angle of 90°. Fifteen b0 images (b = 0 s/mm^2^ and 5 b0 images per 20 diffusion-weighted images) were acquired. The total acquisition time was approximately 20 min.

### 2.7 Diffusion tensor imaging data analysis

The statistical parametric mapping-compatible DTI toolbox was used in the statistical process for data calibration and voxel-based analysis. The DTI Studio software1 was used to compensate for motion, and the diffusion-weighted image was linearly registered. DTI Studio calculated the Apparent Diffusion Coefficient (ADC) values. All normalized ADC images were cropped to 1.0 × 1.0 × 1.5 mm^3^ voxels (after zooming) for Gaussian smoothing (2.0 × 2.0 × 4.0 mm^3^ voxels [Gaussian kernel]), and a voxel-based analysis was performed. In the entire brain, 100 + voxel clusters with significant differences in ADC between groups (*p* < 0.005) were marked. The statistical analysis was performed using the SPM12 and spm rat IHEP toolboxes2. The data were spatially normalized to allow for voxel comparisons between animals. Data were compared between groups using an analysis of variance (ANOVA). The critical t-value was determined with 3.169 [t(10) = 3.169, *p* = 0.005].

### 2.8 RNA-seq

The Cerebellum from 6 mice aged 12week were isolated and harvested in RNA later. Cerebellum lysates from 3 mice in each group were collected for total RNA isolation, following the instructions of the manufacturers of the Trizol and RNeasy Lipid Tissue Mini Kit (QIAGEN, Hilden, Germany). Samples were eluted in 15μL of RNase-free H2O and quantified using a Nanodrop ND-1000 (Thermo Fisher Scientific, Inc., Waltham, MA, USA) spectrophotometer. Agilent 2100 Bioanalyzer and 2100 RNA nano 6000 assay kit (Agilent Technologies) were used to evaluate the integrity of RNA samples. All samples were adjusted to 100 ng/μL and only used when the RNA integrity number (RIN) was ≥9.0. Library preparation and sequencing were performed with NextSeq500 (Illumina, Inc., San Diego, CA, USA) at Capital Bio Technology (Beijing, China). After sequencing, the color-space data was demultiplexed and aligned against the Mus_musculus.GRCm39/mm10 mouse genome with LifeScope using the paired-end whole transcriptome module (Thermo Fisher Scientific, Waltham, MA, USA).

### 2.9 Gene expression analyses and gene expression verification

Raw data (raw reads) of fastq format were firstly processed through in-house perl scripts. In this step, clean data (clean reads) were obtained by removing reads containing adapter and trimming low quality base with Trimmomatic. At the same time, Q20, Q30 and GC content the clean data were calculated. All the downstream analyses were based on the clean data with high quality.

(For DESeq2 with biological replicates) Differential expression analysis of two conditions/groups (two biological replicates per condition) was performed using the DESeq2 R package (1.16.1). DESeq2 provide statistical routines for determining differential expression in digital gene expression data using a model based on the negative binomial distribution. The resulting P-values were adjusted using the Benjamini and Hochberg’s approach for controlling the false discovery rate. Genes with an adjusted *P*-value <0.05 found by DESeq2 were assigned as differentially expressed.

(For edgeR without biological replicates) Prior to differential gene expression analysis, for each sequenced library, the read counts were adjusted by edgeR program package through one scaling normalized factor. Differential expression analysis of two conditions was performed using the edgeR R package (3.18.1). The P values were adjusted using the Benjamini & Hochberg method. Corrected *P*-value of 0.05 and absolute foldchange of 2 were set as the threshold for significantly differential expression.

To confirm the results of sequencing, firstly, the simple random sampling method was used in selecting RNAs with random numbers generated in Microsoft EXCEL. Total RNAs of cerebellum in mice were extracted using TRIzol reagent (Invitrogen, USA), and reversely transcribed by reverse transcriptase according to the manufacturer’s protocol. Then, RT-qPCR was performed with SYBR Green qPCR Master Mix (Bio-Rad, USA) on CFX96 real-time PCR system. The used primers were listed in **Table 1**. The relative quantification of mRNAs were normalized to GAPDH with the 2^−ΔΔCT^ method.

**Table 1:** Primer sequences used for qPCR in this study.

Gene name	Forward primer 5’-3’	Reverse primer 5’-3’
syne1	ICAGCAGTCTGTGACGGTTC	ACGACTTGAGGGCAGACTTG
hs6st3	CAACAACCGCCAAGTCCGCA	TCCTCAGTGACAGTCCCTCTA
Tnfrsf18	CACTGCGGAAACCCTTGCT	AACACGGTGAGAAACCCAAACT
STXBP51	AGGGACTTGCCTTGTCGCTGAT	GAG ATTTAGGTGGGG ACGCTGC
Soga1	CTT GCC TTC TCC GAC CTC AC	GACCTTGACATCCTGCTCCA
Lin7a	GCAACAGCAAAGGCAACAGT	CTCTTTTGAGGCCTCCGTGT

### 2.10 Gene co-expression network analysis

The resulting counts tables were loaded into R (version 3.5.1). Counts data were transformed using DESeq2’s rlog function. Surrogate variable analysis (sva, v3.28.0) was used to remove unwanted variation based on the study design and the detected surrogate variables were regressed out of the normalized count matrix. The normalized, SVA-corrected data were used in WGCNA analysis using the block wise Modules function. For each gene, we report the kME (correlation of a gene with the eigengene of the module) across all modules, the p-value across all modules, the module assigned by WGCNA, and whether the WGCNA-assigned module had the best p-value or kME for that module.

### 2.11 Statistical analysis

Data are expressed as mean ± Standard Error of the Mean (SEM) and analyzed using GraphPad Prism 8 software (La Jolla, California, USA). Data followed normal distribution as evidenced by the Shapiro–Wilks normality test with the hypothesis for normality rejected at a p value less or equal to 0.05. and for homogeneity of variance using the Levene’s test. Data were analyzed using Student’s t test. For all analyses Statistical significance was set at *p < 0.05, **p < 0.01, ***p < 0.005.

## 3 Results

### 3.1 Trio deletion in PCs leads to abnormal spontaneous activity and motor coordination

**Figure 1:**
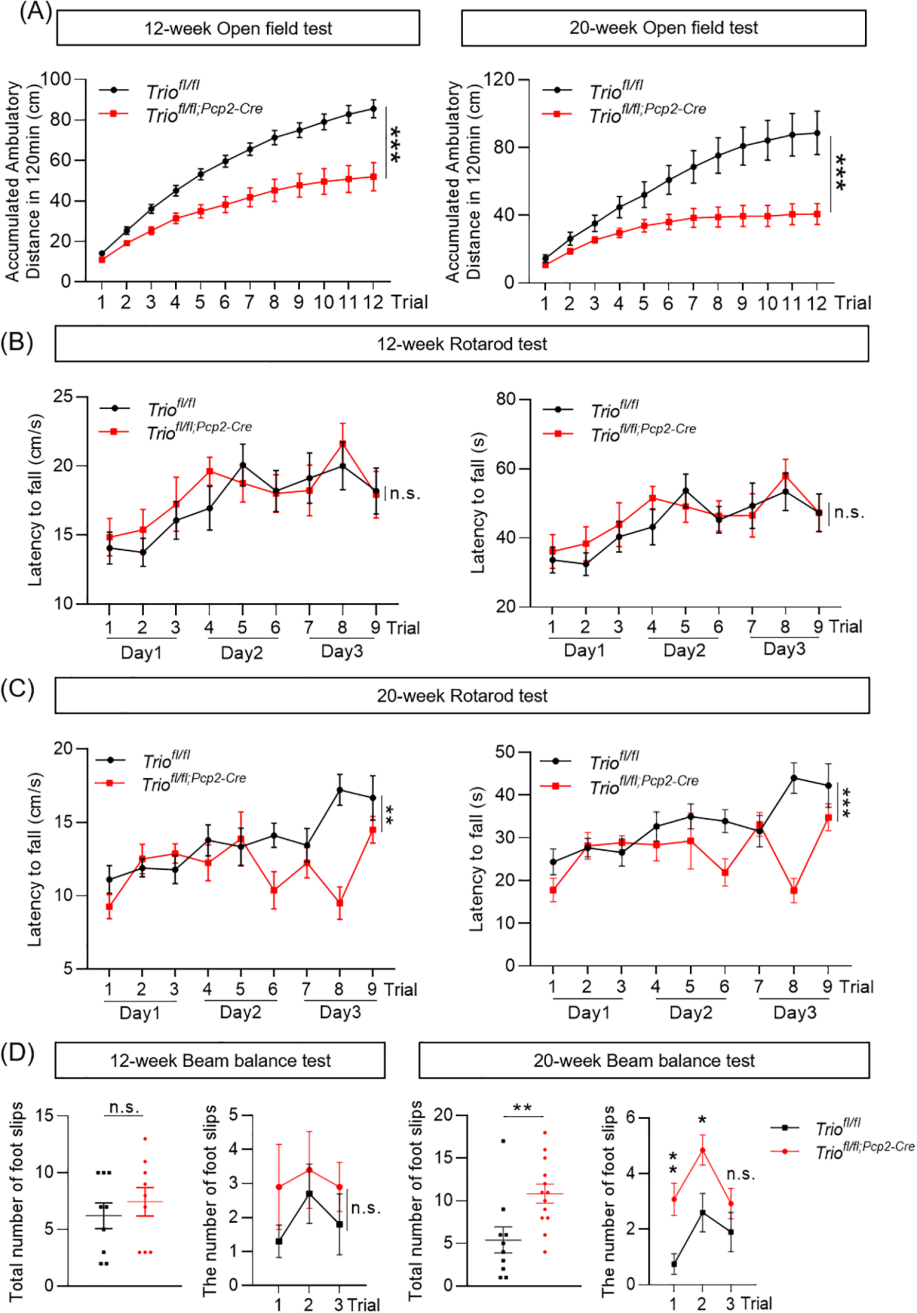
Trio deletion in PCs lead*s* to abnormal spontaneous activity and motor coordination. The ambulatory distance decreased in both 12-week-old and 20-week-old cKO mice. **(A)** The ambulatory distance in the 12-week-old and 20-week-old mice in open field test. (12 week: WT n =10, cKO n =10; 20 week: WT n =10, cKO n =10). The data were expressed as mean ± SEM; Unpaired t-test, *p < 0.05, **p < 0.01, ***p < 0.001. **(B)** The fall latency and speed in the 12-week-old mice in rotarod test (WT n =10, cKO n =13). **(C)** The fall latency and speed in the 20-week-old mice in rotarod test (WT n =10, cKO n =13). **(D)** The number of foot slips in beam balance test in 12 and 20-week-old mice (WT n =16, cKO n =13). The data were expressed as mean ± SEM; Unpaired t-test, *p < 0.05, **p < 0.01, ***p < 0.001. ns, not significant.

The spontaneous locomotion activity of *Trio^fl/fl;Pcp2-Cre^* (cKO) mice was assessed by using the open field test at both 12-week-old and 20-week-old, with *Trio^fl/fl^* mice as the control group. Compared to their counterparts, the cKO mice exhibited a significant decrease in total distance traveled over a 2-hour period (**Figure 1A**), at both ages evaluated. For evaluating the motor coordination, the rotarod test was employed. No significant differences in latency to fall or falling speed were observed between the cKO and control groups at the age of 12 weeks (**Figure 1B**). However, at 20 weeks of age, the cKO mice demonstrated a significant reduction in latency to fall and falling speed on Day3 (**Figure 1C**). The balance beam test was performed to evaluate the balance ability and fine motor coordination. Slight increases, but statistically insignificant, was observed in the cKO mice at the age of 12 weeks compared to control, with no substantial increase in the number of foot slips (**Figure 1D**). In contrast, a significant increase in the number of foot slips was noted in the 20-week-old cKO mice (**Figure 1D**), suggesting impaired motor function in the cKO mice at this age. These results indicated that the mice with *Trio* deletion in PCs displayed progressive motor impairments.

### 3.2 Trio deletion in PCs resulted in gait abnormalities

**Figure 2:**
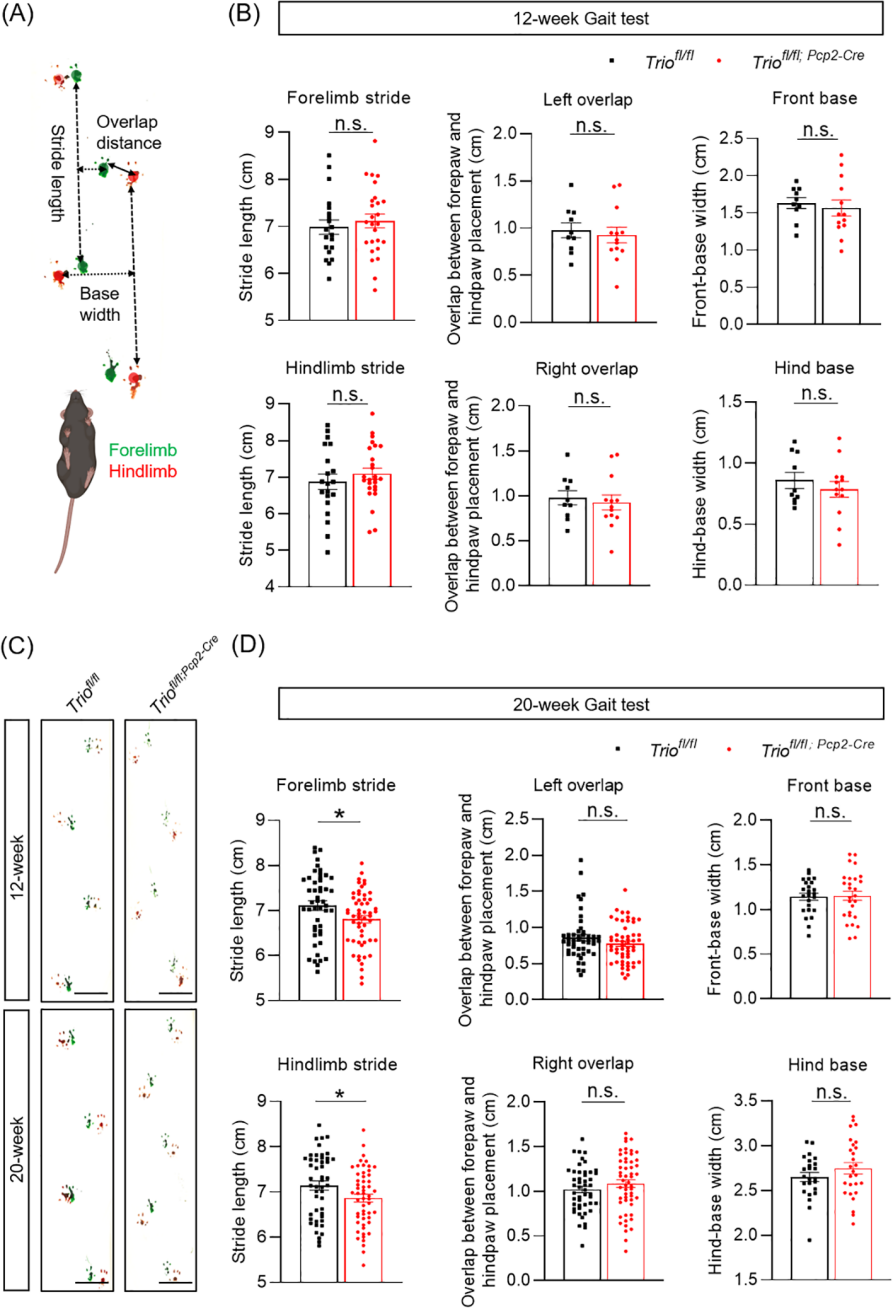
Trio deletion in PCs resulted in gait abnormalities. Gait analysis in 12 weeks age and 20 weeks age in two group. **(A)** Gait test Schematic diagram. Gait was analyzed during spontaneous walk. Width length and overlap between forepaw and hind paw placement (mm) in left and right. **(B)** Gait analysis in 12-week-old and 20-week-old mice. **(C)** Width length and overlap between fore paw and hind paw placement(cm) in left and right in the 12-week-old mice in rotarod test (WT n =10, cKO n =13). **(D)** Width length and overlap between forepaw and hind paw placement (mm) in left and right 20-week-old mice (WT n =16, cKO n =13). All data are expressed as means ± SEM. Unpaired t-test analysis was performed, *p < 0.05. ns, not significant.

We performed gait test analysis to evaluate the gait characteristics of cKO mice (**Figure 2A**). The results showed that the decline of stride length of both the front and rear limbs were statistically significant between 20-week old WT and cKO mice (*p < 0.05*) (**Figure 2B**). The front and rear limb distances and the overlap of footprints did not show significant differences between the two groups (**Figure 2C**). However, no significant differences were observed between the two genotypes in the 12-week-old mice (**Figure 2D**).

### 3.3 Abnormal morphology of cerebellum in mice with Trio deletion in PCs

**Figure 3:**
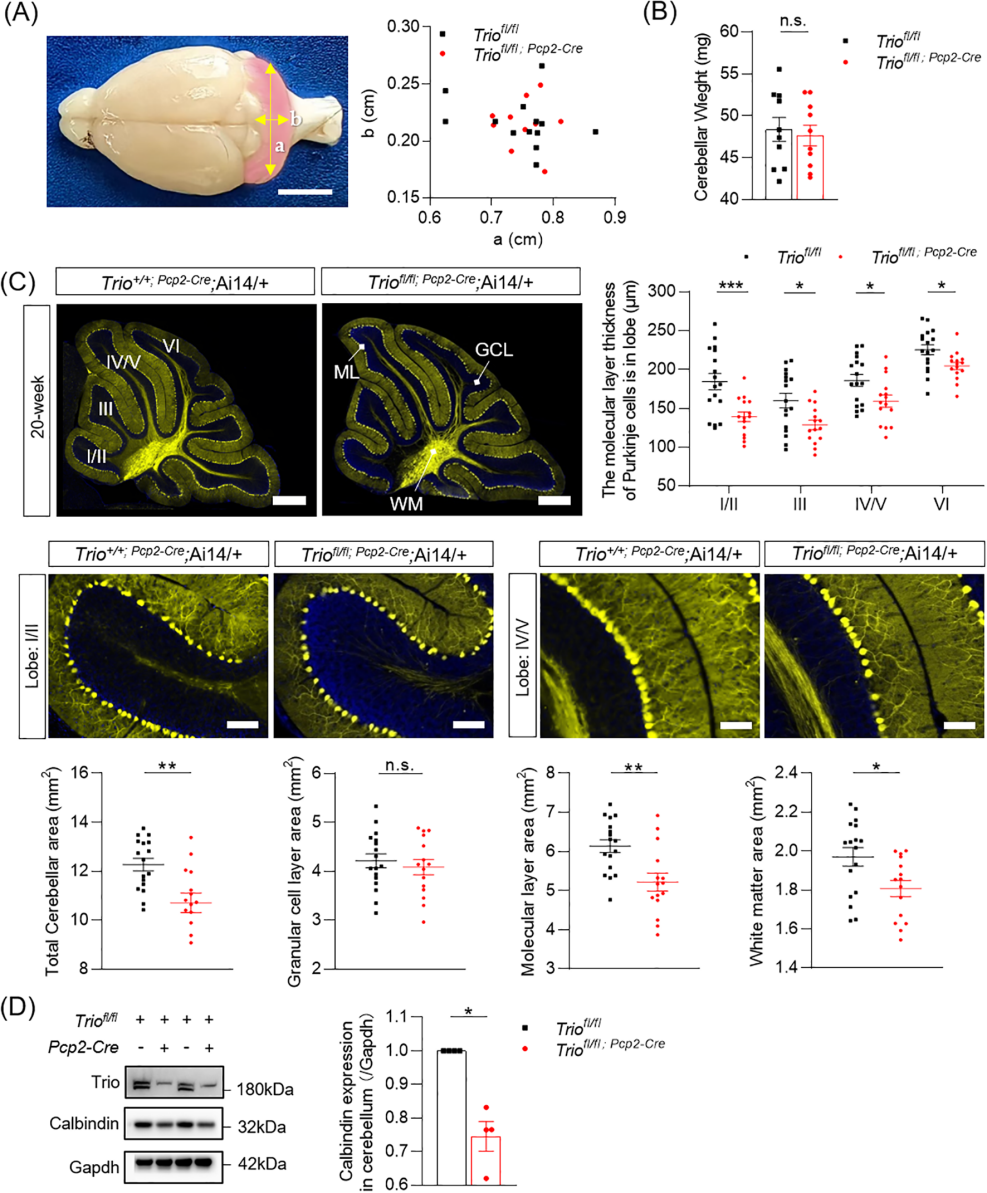
Abnormal morphology of cerebellum in 12-week-old Mice with *Trio* deletion in PCs. **(A)** the size of the 12-week-old cerebellum in the direction of the two axes **(B)** 12-week-old cerebellar weight (WT n = 10, cKO n = 10); **(C)** Cerebellar molecular layer (lobe I/II,III,IV/V,VI) thickness(μm) and total cerebellar aera(mm^2^), molecular layer area(mm^2^), granlule cell layer area(mm^2^) and white matter area (mm^2^) (WT n = 16, cKO n = 17). (All data are expressed as means ± SEM; Unpaired t-test, *p < 0.05, *p < 0.01). **(D)** Calbindin expression levels in 12-week-old mice of two group. (WT n = 4, cKO n = 4). The data were expressed as mean ± SEM; One simple test and Wilcoxon, *p < 0.05, **p < 0.01, ***p < 0.001.. ns, not significant.

We first determined the size of cerebellum in cKO mice and their control littermates in two principal orthogonal axes. There were no significant differences between the two genotypes in the 12-week-old mice (**Figure 3A**). Meanwhile, the weight of cerebellum in two group is not significant (**Figure 3B**). We measured the total area of the mid-sagittal cerebellum and found a significant decrease in cKO mice compared to the controls (**Figure 3C**, *p <* 0.05) in 12-week-old mice. Further analysis revealed that the reduction of the molecular layer (ML) and white matter (WM) area in cKO mice, with statistically significant differences (**Figure 3C**, *p <* 0.05). We also measured the averaged ML thickness in the mid-sagittal sections of cerebellar vermis lobes I/II, III, IV/V, and VI and found varying degrees of reduction in different lobes in the 12-week-old mice. Lobes I/II (*p <* 0.001*)* and IV/V (*p <* 0.001) showed statistically significant differences. Lobe III and VI showed a trend of decreased thickness, but the differences were not statistically significant in the 12-week-old mice. Additionally, these findings indicate that significant alterations in the cerebellum occur at 12 weeks of age, affecting major lobes related to motor function.

**Figure 4:**
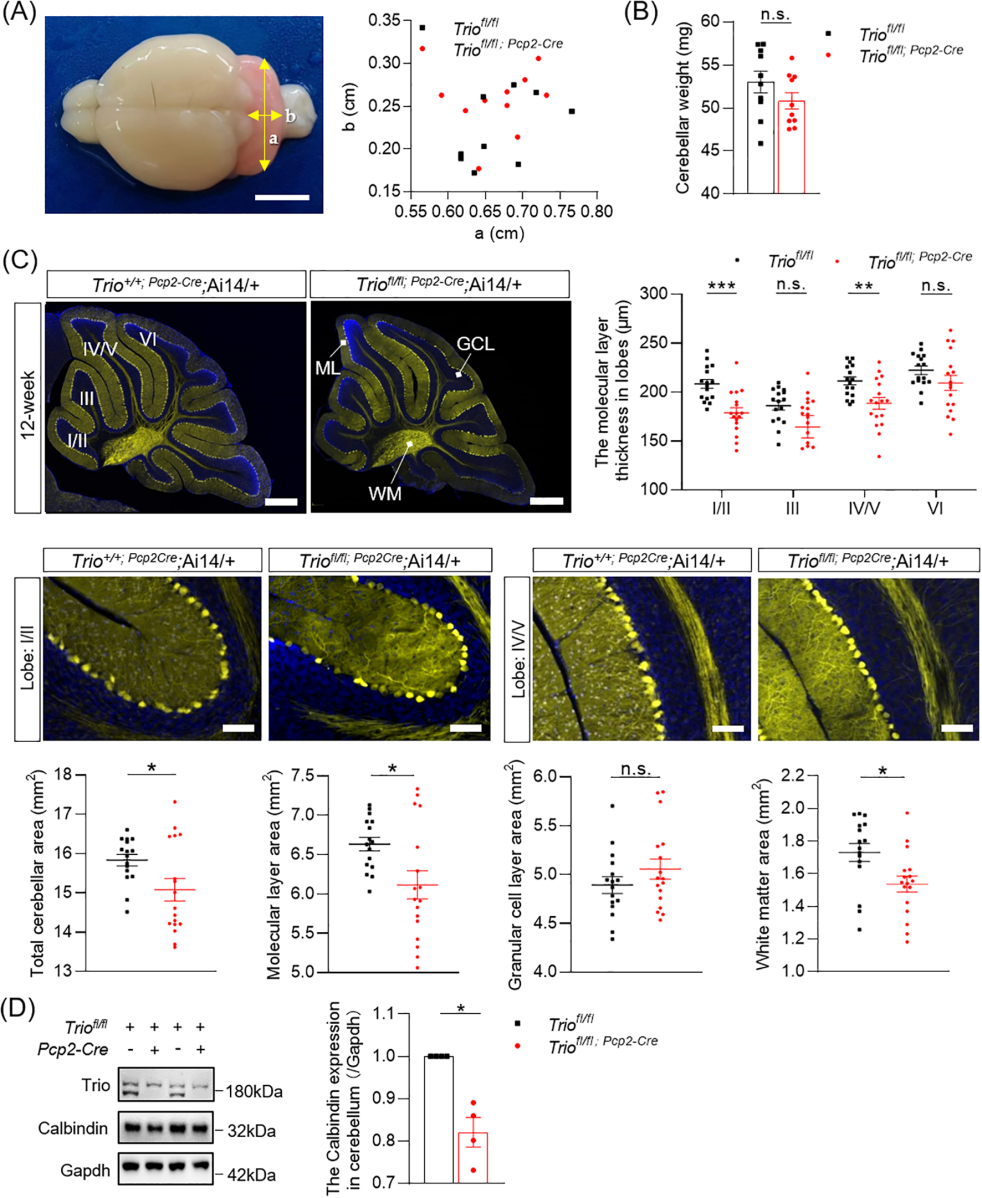
Abnormal morphology of cerebellum in 20-week-old Mice with Trio deletion in PCs. **(A)** the size of the 20-week-old cerebellum in the direction of the two axes. **(B)** 20-week-old cerebellar weight (WT n = 10, cKO n = 10); **(C)** Cerebellar molecular layer (lobe I/II,III,IV/V,VI) thickness (μm) and total cerebellar aera (mm^2^), molecular layer area(mm^2^), granular cell layer area (mm^2^) and white matter area (mm^2^) (WT n = 17, cKO n = 15). The data are expressed as means ± SEM; Unpaired t-test. **(D)** Calbindin expression levels in 20-week-old mice of two group (WT n = 4, cKO n = 4). The data were expressed as mean ± SEM; One simple test and Wilcoxon, *p < 0.05, **p < 0.01, ***p < 0.001. ns, not significant.

Then we determined the morphological change of cerebellum in 20-week-old mice with *Trio* deletion in PCs. We measured the length of cerebellum in cKO mice and their control littermates in two principal orthogonal axes. There were no significant differences between cKO mice and controls in 20 weeks (**Figure 4A**). The weight of cerebellum also showed no significant difference between the two genotypes (**Figure 4B**). The total area of the mid-sagittal cerebellum show a significant decrease in cKO mice compared to controls (**Figure 4C**, *p <* 0.05) in the 20-week-old mice. Further analysis revealed that the reduction in the ML and WM area, with statistically significant differences (**Figure 4C**, *p <* 0.05) in the 20-week-old mice. We also measured the average ML thickness in the mid-sagittal sections of cerebellar vermis lobes I/II (*p <* 0.001), III (*p <* 0.05), IV/V (*p <* 0.05), and VI (*p <* 0.05) and found varying degrees of reduction in different lobes in the 20-week-old mice, with statistically significant differences. In mice aged 20 weeks, there was an increased number of affected lobules compared to 12-week-old mice, indicating that the *Trio* deletion-induced impairment was more severe in the 20-week-old mice.

Furthermore, the expression levels of the PCs’ marker Calbindin was persistently decreased in both 12-week-old and 20-week-old mice (**Figures 3D**, **4D**), with statistically significant differences (*p <* 0.05).

### 3.4 Abnormal ADC values in the cerebellum of 12-week-old mice with Trio deletion in PCs

**Figure 5:**
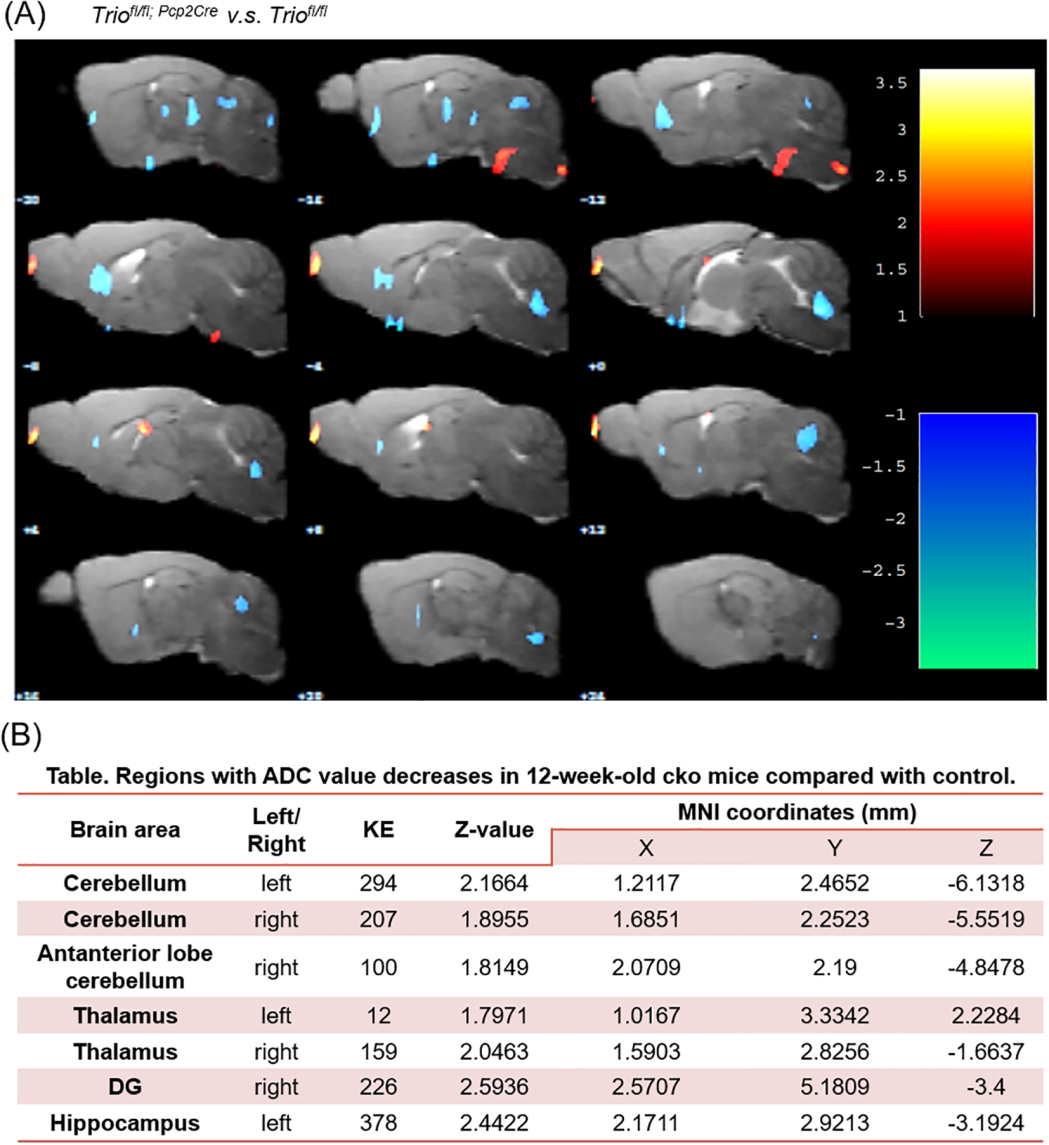
A significant decreasing of ADC value in cerebellum region of 12-week old *Trio^fl/fl; Pcp2-Cre^* mice compared with control. **(A)** The comparison between two group in ADC value in Voxel-based statistical analysis, **(B)** Regions with ADC value decreases in 12-week-old *Trio^fl/fl; Pcp2-Cre^* mice compared with control. The significant main effects of the phenotype are shown for the mean diffusivity. Results are displayed on an anatomical template with a visualization threshold of false discovery rate with *p* < 0.005 and a minimum cluster size of 100 voxels. The color scale indicates f-values, with yellow indicating a larger difference between the conditions investigated. MNI, Montreal Institute of Neurology (X, Y, and Z coordinates locate anatomical structures). KE, number of continuum elements; Z-value, activation cluster peak value. Data are expressed as the mean ± SEM (n = 6/group). ADC, Apparent Diffusion Coefficient.

To further investigate the white matter of the brain changes in the cerebellum, we performed DTI-MRI measurements. T2 and DTI sequences were acquired. The VBA algorithm revealed a significant decrease in apparent ADC (**Figures 5A, B**) values in the first cerebellar lobule of 12-week-old cKO mice compared to the control group (*p <* 0.05).

### 3.5 Gene expression changes in 12-week old Trio^fl/fl; Pcp2-Cre^ mice in RNA-seq analysis and gene expression verification

**Figure 6:**
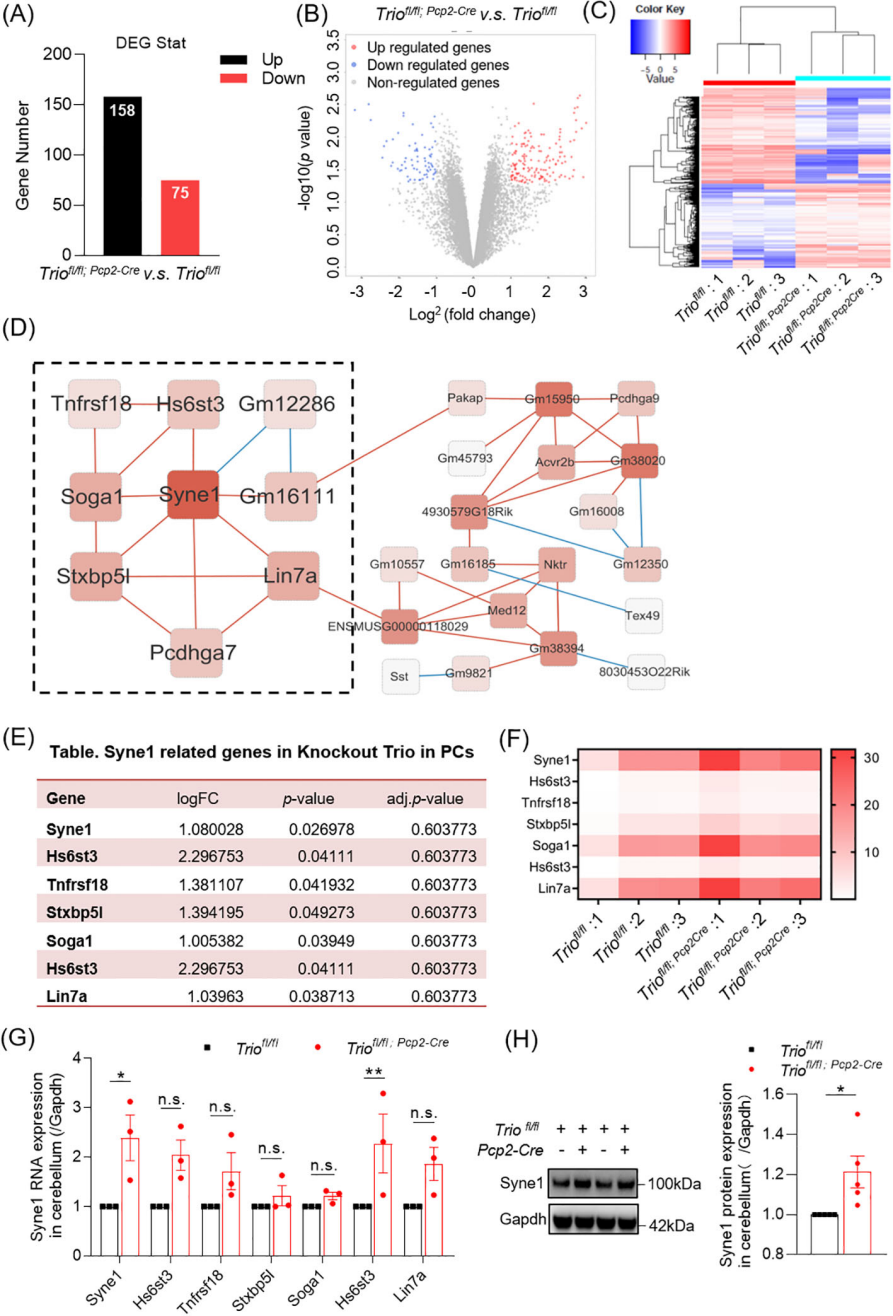
The Gene Expression Changes in cerebellum with *Trio* deletion in PCs. **(A)** Overview of Gene Expression Changes. Number of upregulated and downregulated differentially expressed genes (DEGs) in cerebellum. **(B)** Differential gene expression detected with a false discovery rate (FDR)-corrected *p* < 0.05 (n = 3/group). **(C)** Heat plot in Cluster analysis displaying expression upregulated and downregulated cerebral DEGs in wild-type and cKO samples determined by *log*FC values with adjusted *p* < 0.05. **(D)** The cluster resulting from WGCNA (Weighted correlation network analysis) analysis shown on the basis of Syne1 function. **(E)** Syne1 correlated network genes. **(F)** Heatmap in RNA expression levels of Syne1 correlated network genes. **(G)** The RNA expression of Syne1 and its related gene in cerebellum. (WT n=3; cKO n=3). **(H)** The protein expression of Syne1 in cerebellum. (WT n=5; cKO n=5). The data were expressed as mean ± SEM; One simple test and Wilcoxon, *p < 0.05, **p < 0.01. ns, not significant.

To gain further insights into the difference of downstream gene expression levels in the cerebellum by *Trio* deletion in PCs, we performed RNA sequencing. We identified 158 upregulated genes and 75 downregulated genes in cKO group compared to the control group (**Figure 6A**). Differentially expressed gene clustering analysis revealed the involvement of multiple pathways, including cell cytoskeleton, cell apoptosis, and neurodegenerative diseases (**Figure 6B**). Among the 158 upregulated genes, we found that Syne1 was associated with cerebellar ataxia (**Figure 6C**). Co-expression analysis of Syne1 revealed co-expressed genes, including Hs6st3, Tnfrsf18, Stxbp5l, Soga1, Hs6st3, and Lin7a, with statistically significant differences (**Figures 6D–F**). We performed verification of mRNA expression profiles with RT–qPCR. The expression level of mRNA and protein in Syne1 was decreased in 12-week old *Trio^fl/fl;Pcp2-Cre^* mice compared with controls, with statistically significant differences (**Figures 6G, H**).

## 4 Discussion

Here we found that mice with Trio deletion in PCs exhibited delayed-onset motor dysfunctions, including gait abnormalities and deficits in balance function, which are also observable at 20 weeks of age and highly associated with cerebellar anomalies. MRI-DTI data indicated chronic injury in the cerebellum. RNA-sequencing also demonstrated that Trio deficiency induces expression alteration in genes related to cerebellar ataxia. Collectively, our findings suggest impairments of PCs in the cerebellum that plays a significant role in the delayed motor abnormalities in the *Trio* deleted ASD mouse.

Motor dysfunctions have long been reported to represent a spectrum of non-diagnostic symptoms in ASD, with an incidence 21-100% rate (20). For example, gait irregularities in ASD patients have been well-documented previously (21). Patients with ASD exhibit various motor symptoms, which include reduced movement activity, repetitive and rhythmic motions, low muscle tone, and difficulties in postural adjustment (22). Given the crucial role of the cerebellar network in motor control, increasing evidence shows that impaired motor functions in autistic patients are highly correlated with cerebellar abnormalities (23, 24). Post-mortem and functional brain imaging studies have also highlighted the role of the cerebellum in the motor deficits of ASD (23).

However, there is a lack of understanding of ASD-related motor impairments. Here we conducted a series of behavioral tests to evaluate the motor dysfunctions in mice with *Trio* deletion in PCs. We found that cKO mice at 12 and 20 weeks of age showed reduced spontaneous locomotion in the open field test. The 20-week-old mice presented with subtle motor dysfunctions, including gait abnormalities, balance, and motor learning deficits. Reports suggest that children with ASD have a shorter stride length (25). Furthermore, human gait analysis encompasses kinematics and dynamics, in rodent models, we analyzed gait changes related only to footprints, which is a limitation of our study.

Consistent with these findings, the cerebellum of Trio-cKO mice had normal size and all lobules were present, quantitative analyses revealed a reduction in cerebellar white matter area in Trio-cko mice. Specifically, reductions in the ML and PCL areas were noted across two distinct developmental stages in 12 week and 20 week. Notably, at the 20-week, there was a discernible decrease in the molecular layer’s thickness, affecting an increased number of lobules, in stark contrast to observations in 12-week-old mice. Simultaneously, we have also observed a reduction in the expression of the characteristic PC marker protein, Calbindin (CB), occurring at both time points. Similar changes have been observed in several other animal models and in clinical patients (9, 11, 26). This phenomenon aligns with age-related changes in motor and balance functions. Therefore, only subtle deviations in spontaneous activity were recorded at 12 weeks, as evidenced by the open field test, by the time the mice reached 20 weeks of age, significant alterations in both balance function and spontaneous activities were showed. These findings suggest that the ramifications of gene knockout magnify with aging. Although Trio plays a critical role during developmental phases, it also could be inferred that it plays a significant role in the degenerative mechanisms unfolding with age-related procession.

Imaging abnormalities are common in patients with ASD. We conducted structural imaging examinations using DTI sequences and T2 scans with VBA comparisons, finding significant ADC value changes in the cerebellar lobules of mice, suggesting long-term chronic damage in the cerebellum. Post-mortem and functional brain imaging studies have clarified the cerebellum’s role in ASD motor deficits (23). Imaging evidence has also indicated functional connectivity abnormalities in the cerebellum of patients with ASD (27).

Through RNA-seq analysis, after Trio knockout in PCs, a significant increase in the expression of Syne1, a gene closely related to balance function, was observed at the RNA level, and co-expressed genes affected motor and coordination abilities. Studies showed that Syene1 mutations cause autosomal recessive cerebellar ataxia (28, 29). Research suggests that Syne1 encodes a spectrum repeat-containing protein, which is expressed in proteins in skeletal muscle, smooth muscle, and peripheral blood lymphocytes, and is localized to the nuclear membrane. Mutations in this gene are associated with autosomal recessive spastic ataxia of Charlevoix-Saguenay, also known as autosomal recessive cerebellar ataxia type 1 or Beauce ataxia. Hence, at the epigenetic level, the knockout of Trio has to some extent affected the expression of genes related to fine motion or balance, potentially partially impacting the motion of mice.

In summary, conditional deletion of Trio in PCs triggered motor dysfunctions in mice at both 12 and 20 weeks of age. Our previous study showed that Trio knockout (*Trio^+/K1431M^* mice) led to core symptoms of ASD, social abnormalities and repetitive behaviors (30, 31). While conditional knockout of *Trio* established a causative link between PCs dysfunction and gait alterations, suggesting that ASD impact extends beyond development. We believe that research focused on motor dysfunctions will contribute to understanding the delayed-onset neurodegeneration in ASD.

## Data Availability

The data presented in the study are deposited in the CNCB repository, accession number CRA023021 (https://bigd.big.ac.cn/gsa/browse/CRA023021).
