# Correction: Deficiency of autism susceptibility gene Trio in cerebellar Purkinje cells leads to delayed motor impairments

**DOI:** 10.3389/fpsyt.2025.1760839

**Published:** 2025-12-26

**Authors:** Jinxin Wang, Yimeng Li, Dai Zhang, Wenzhi Sun, Jun Li

**Affiliations:** 1 State Key Laboratory of Cognitive Neuroscience and Learning and IDG/McGovern Institute for Brain Research, Beijing Normal University, Beijing, China; 2 Chinese Institute for Brain Research, Beijing, China; 3 NHC Key Laboratory of Mental Health, National Clinical Research Center for Mental Disorders, Peking University Sixth Hospital, Peking University Institute of Mental Health, Peking University, Beijing, China

**Keywords:** autism spectrum disorder, trio, motor dysfunctions, cerebellum, Purkinje cells

An incorrect number was provided for the “Non-profit Central Research Institute Fund of Chinese Academy of Medical Sciences”. The correct number is “2023-PT320-08”.

The funder “National Natural Science Foundation of China”, “82471566” to “Jun Li” was erroneously omitted.

The revised version is as follows.

“The author(s) declare financial support was received for the research, authorship, and/or publication of this article. This work was supported by the Key Realm R&D Program of Guangdong Province (2019B030335001), the National Natural Science Foundation of China (82471566, 82271576 and 82071541), and the Nonprofit Central Research Institute Fund of Chinese Academy of Medical Sciences (2023-PT320-08).”

The original article has been updated.

