# Supplementary figures and images for: Deficiency of autism susceptibility gene Trio in cerebellar Purkinje cells leads to delayed motor impairments
Source: Front Psychiatry. 2025 Apr 10;15:1396716. doi: 10.3389/fpsyt.2024.1396716 (PMC12018246; doi:10.3389/fpsyt.2024.1396716)

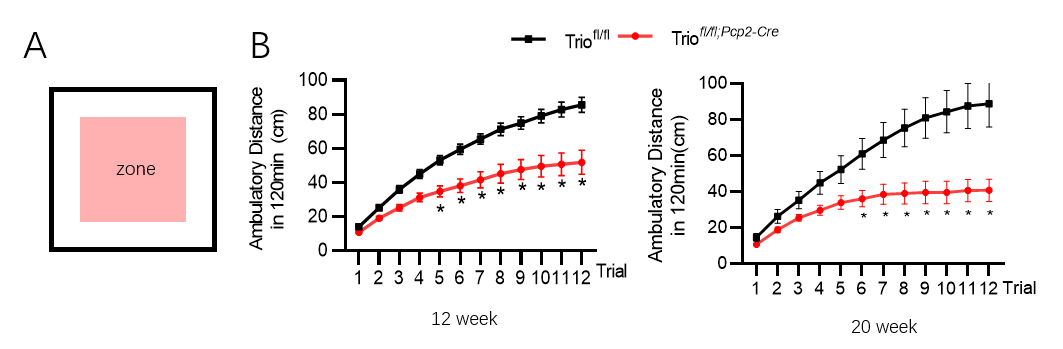

Supplement: Supplementary file 1 [file DataSheet1.zip › fig1/fig1.tif]

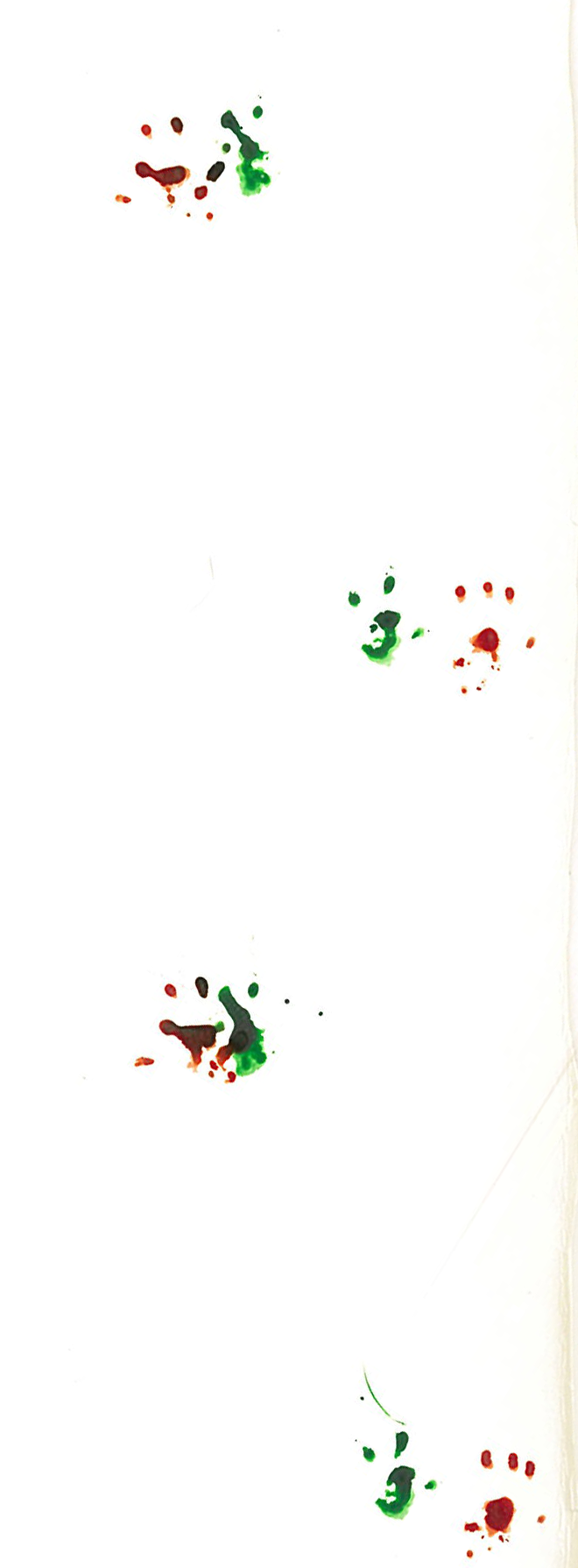

Supplement: Supplementary file 2 [file DataSheet2.zip › fig2/GAIT TEST/12 week ko.tif]

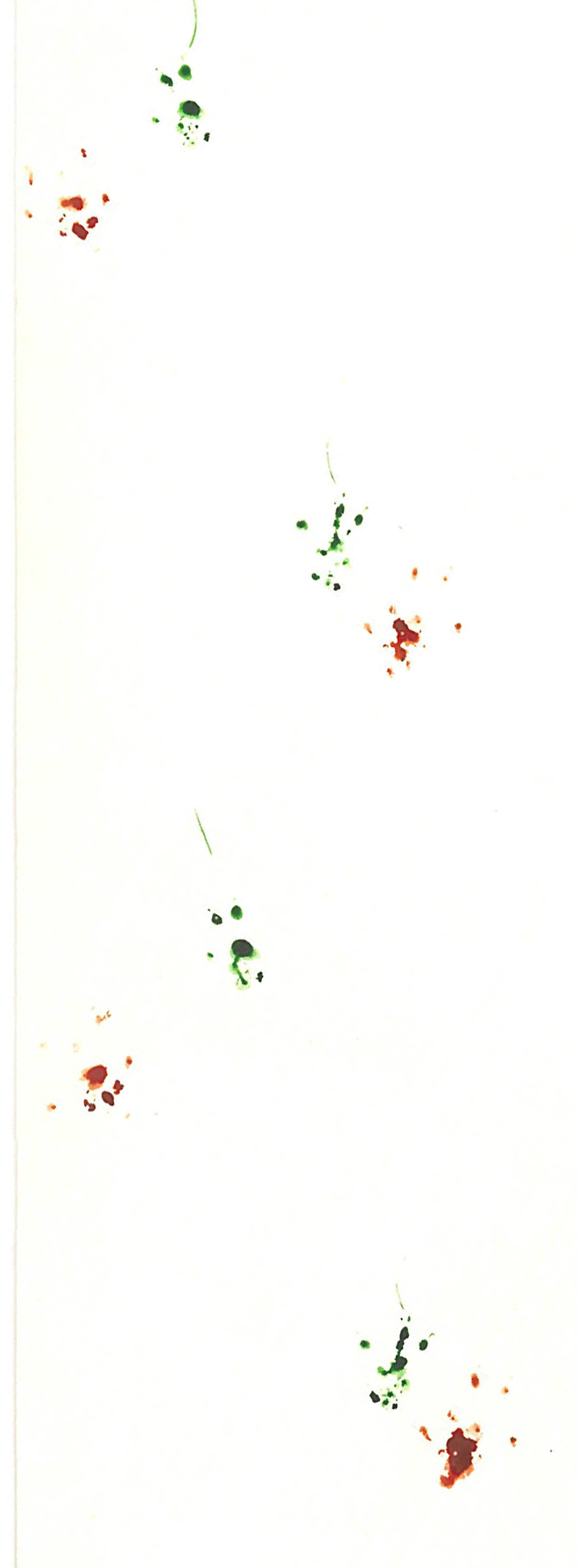

Supplement: Supplementary file 2 [file DataSheet2.zip › fig2/GAIT TEST/12week wt.tif]

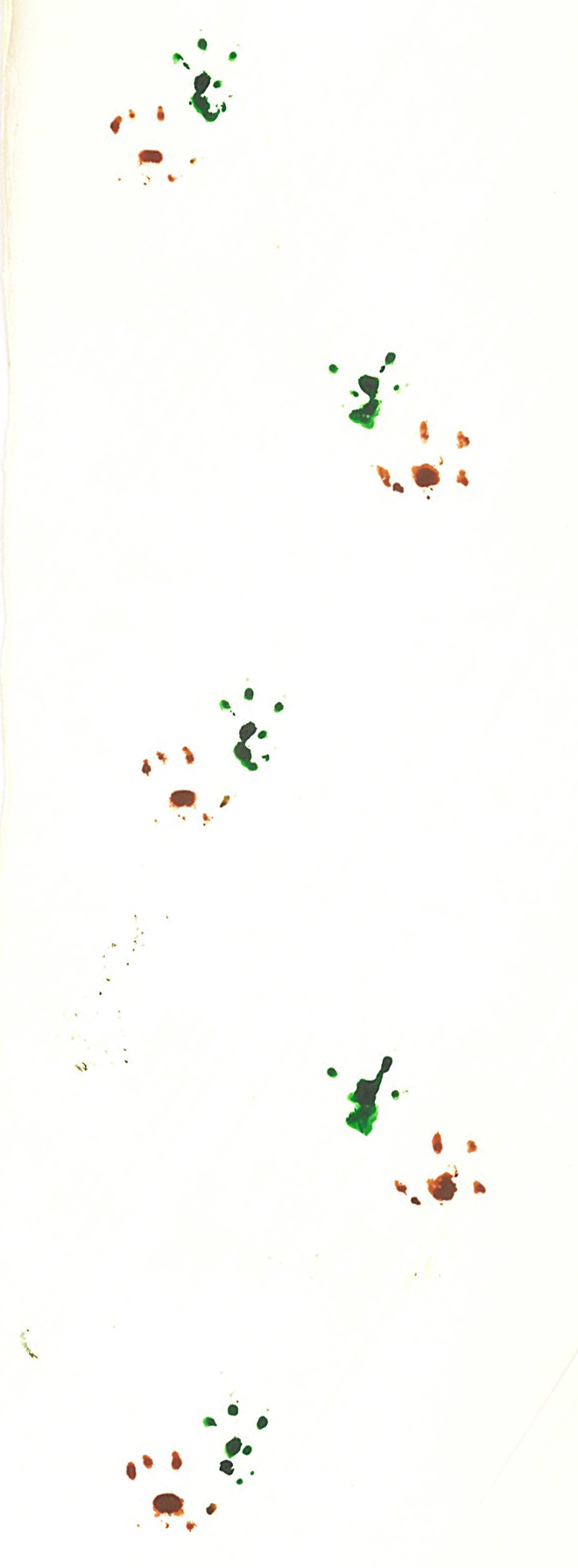

Supplement: Supplementary file 2 [file DataSheet2.zip › fig2/GAIT TEST/20week ko.tif]

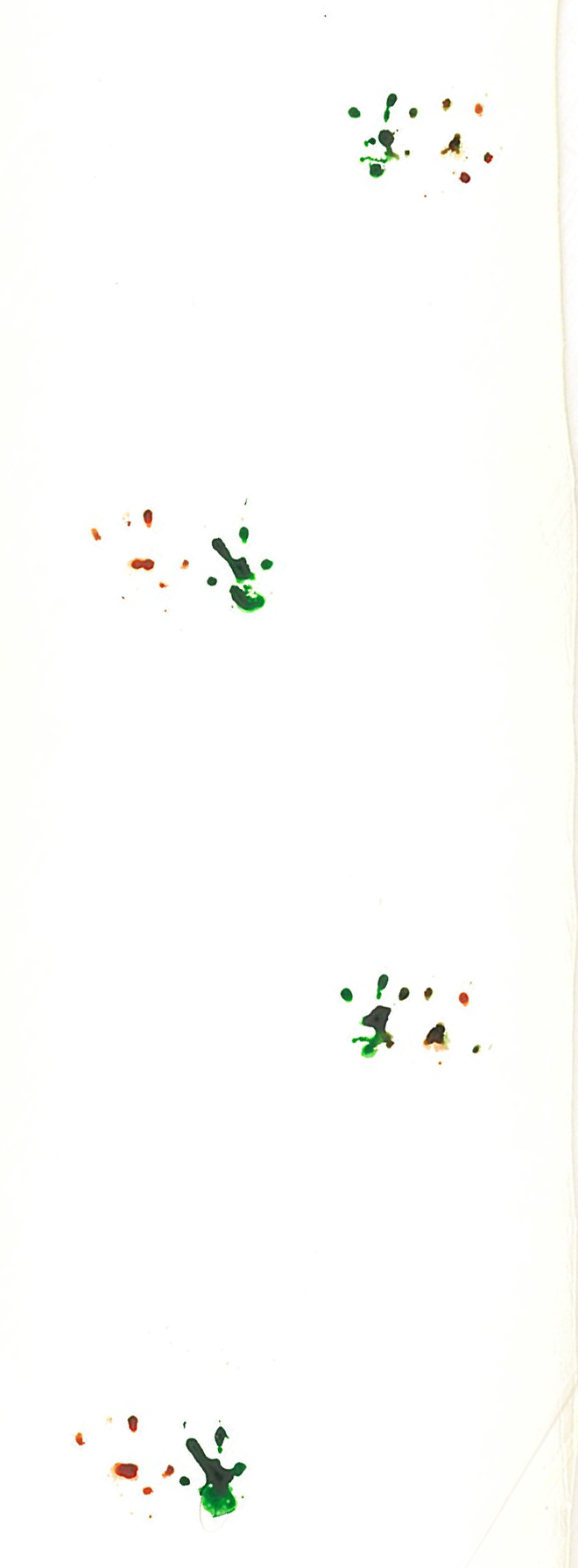

Supplement: Supplementary file 2 [file DataSheet2.zip › fig2/GAIT TEST/20week wt.tif]

112

143

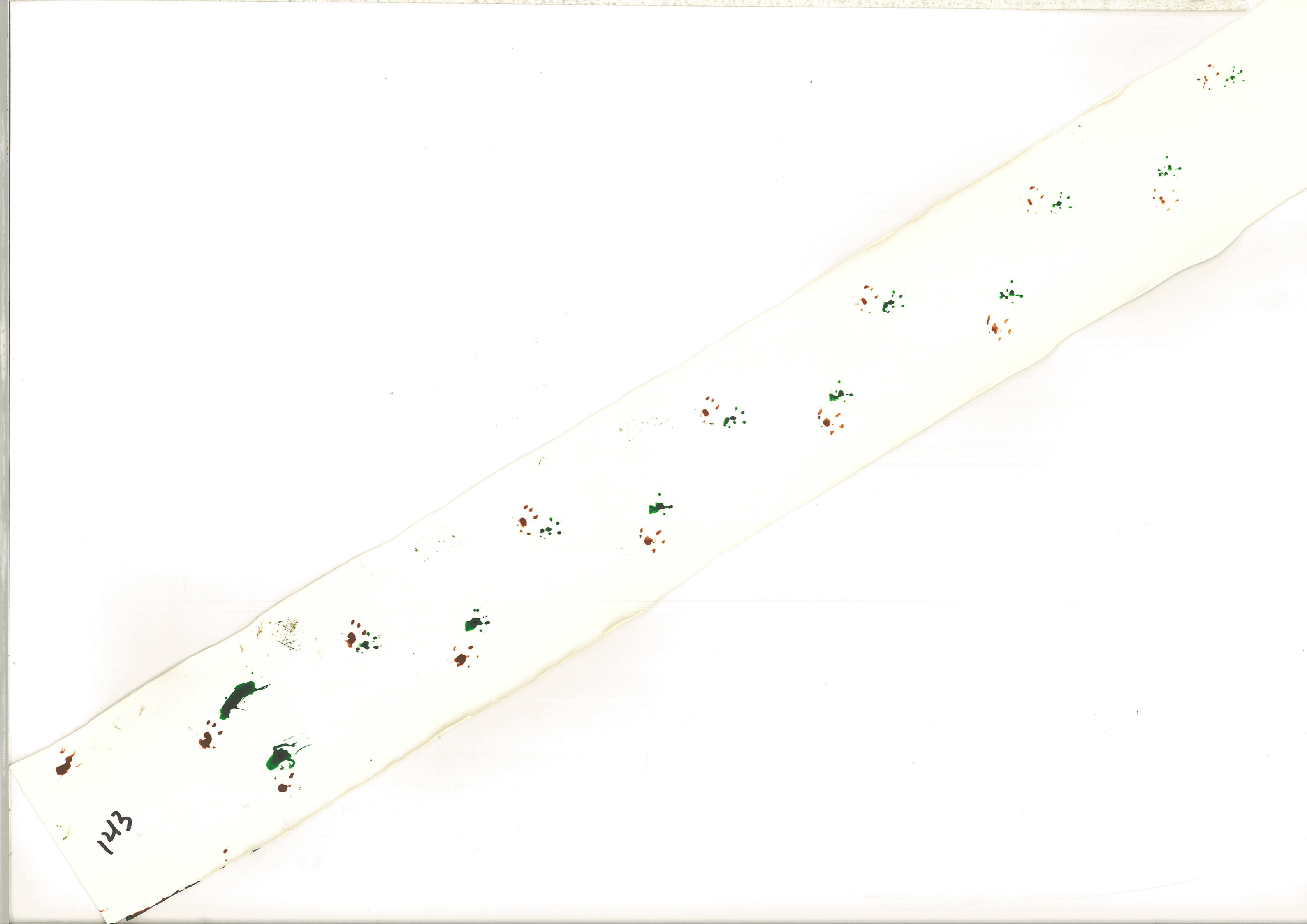

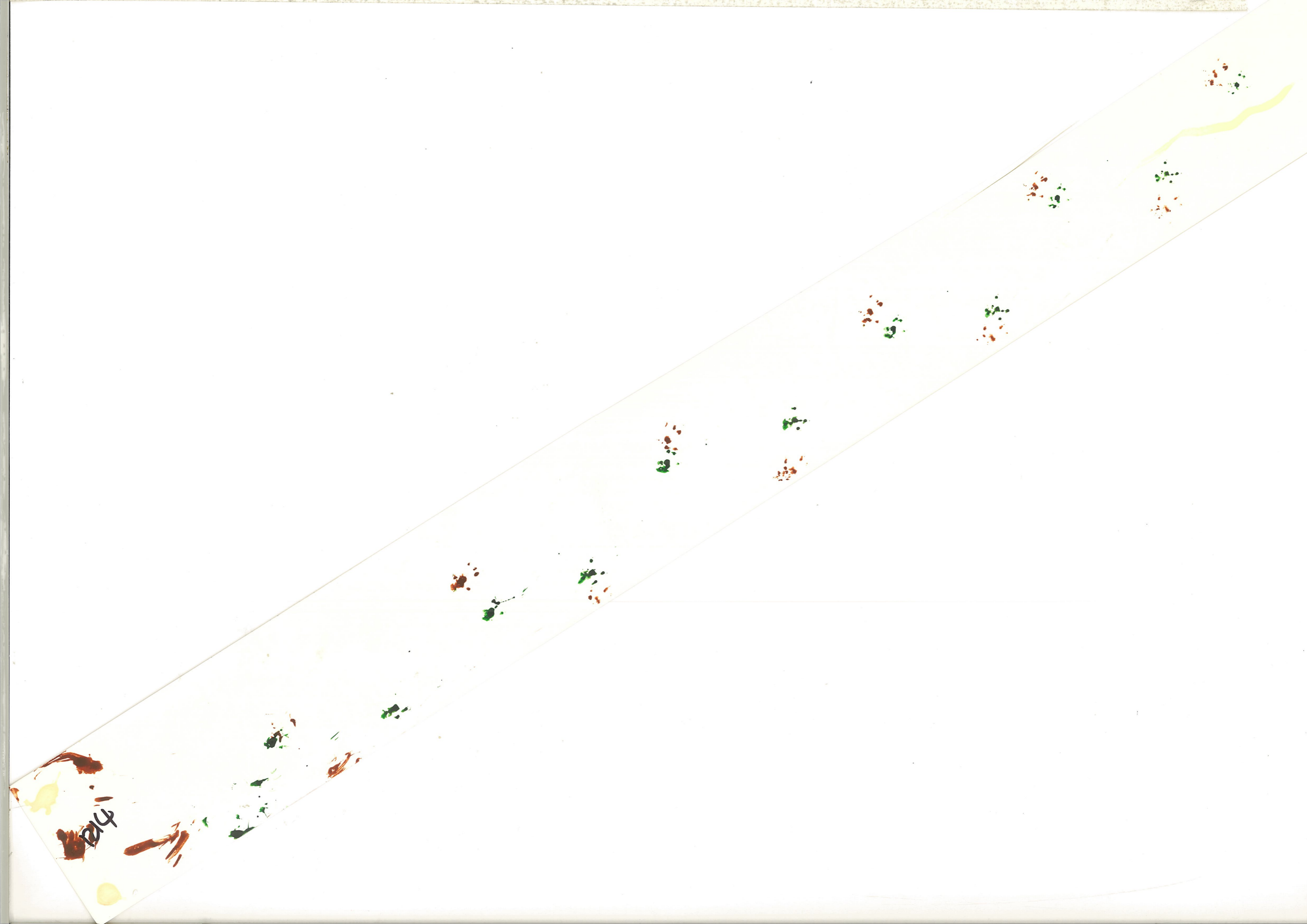

145-571

1331

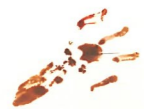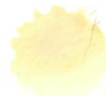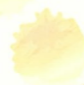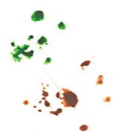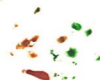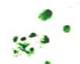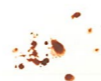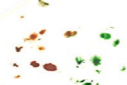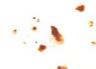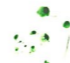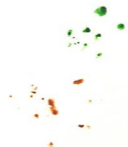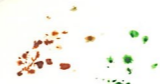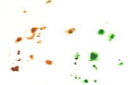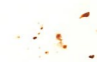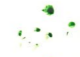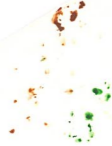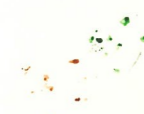

4832

1739

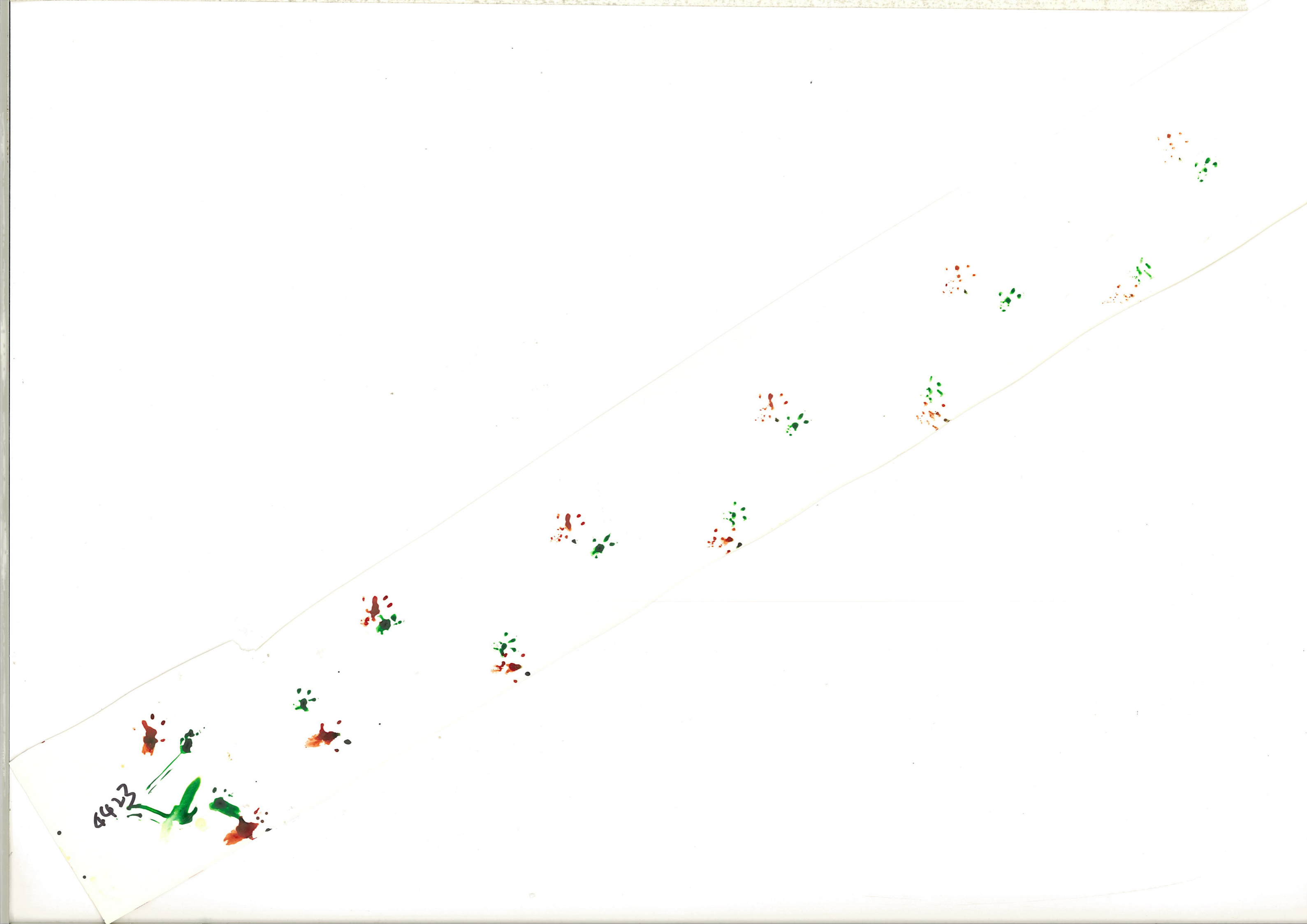

4423

4414

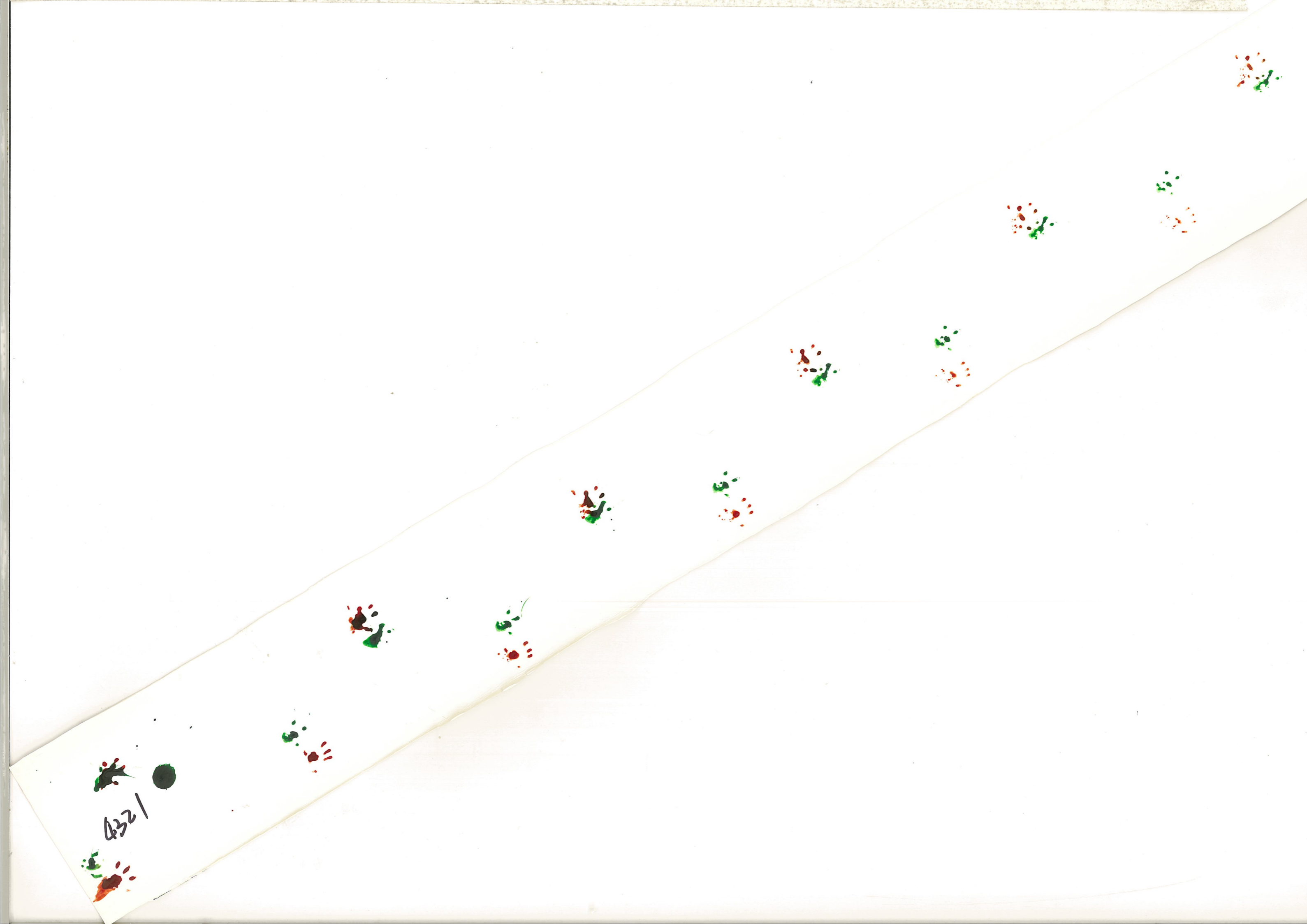

4321

6434

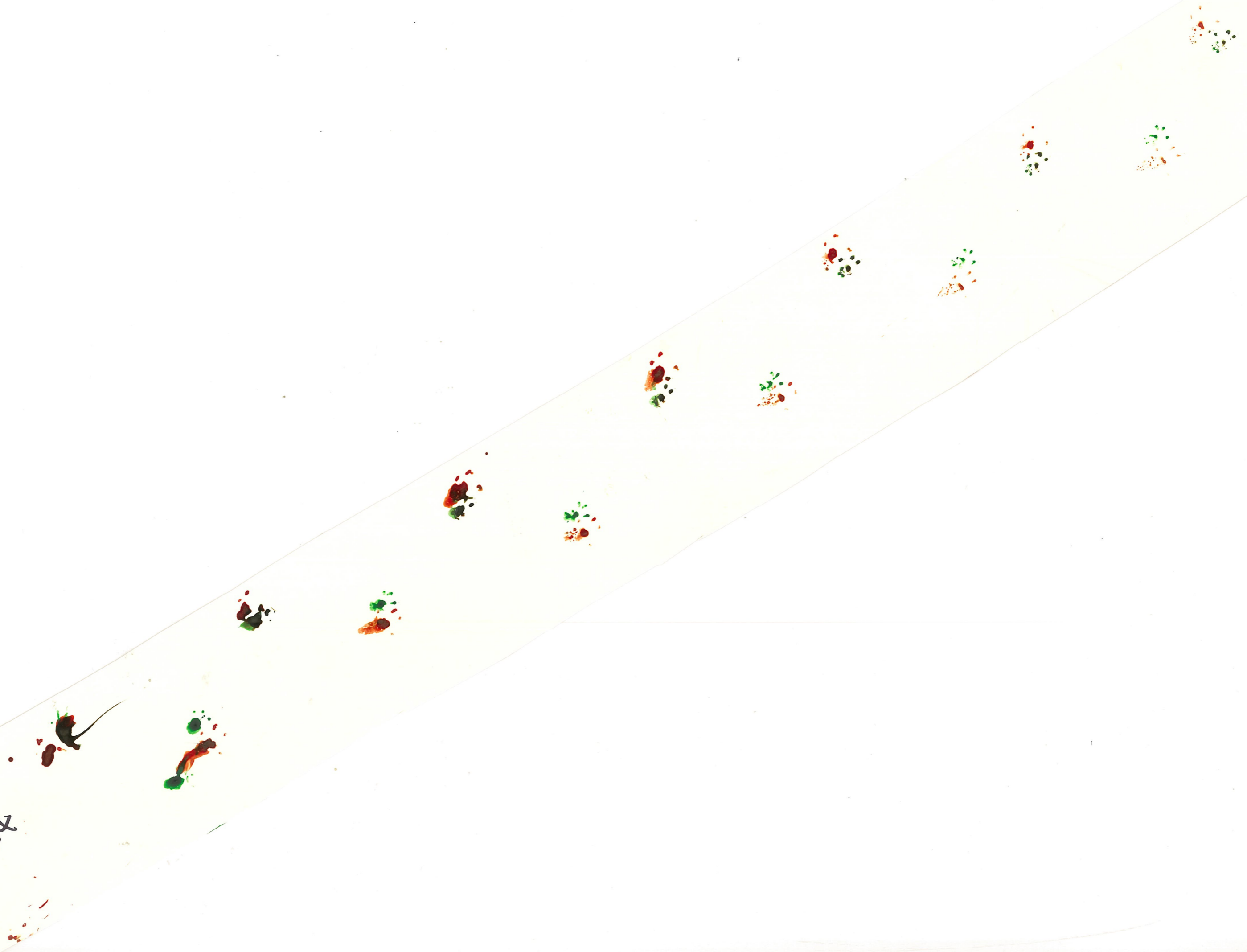

Feb 25

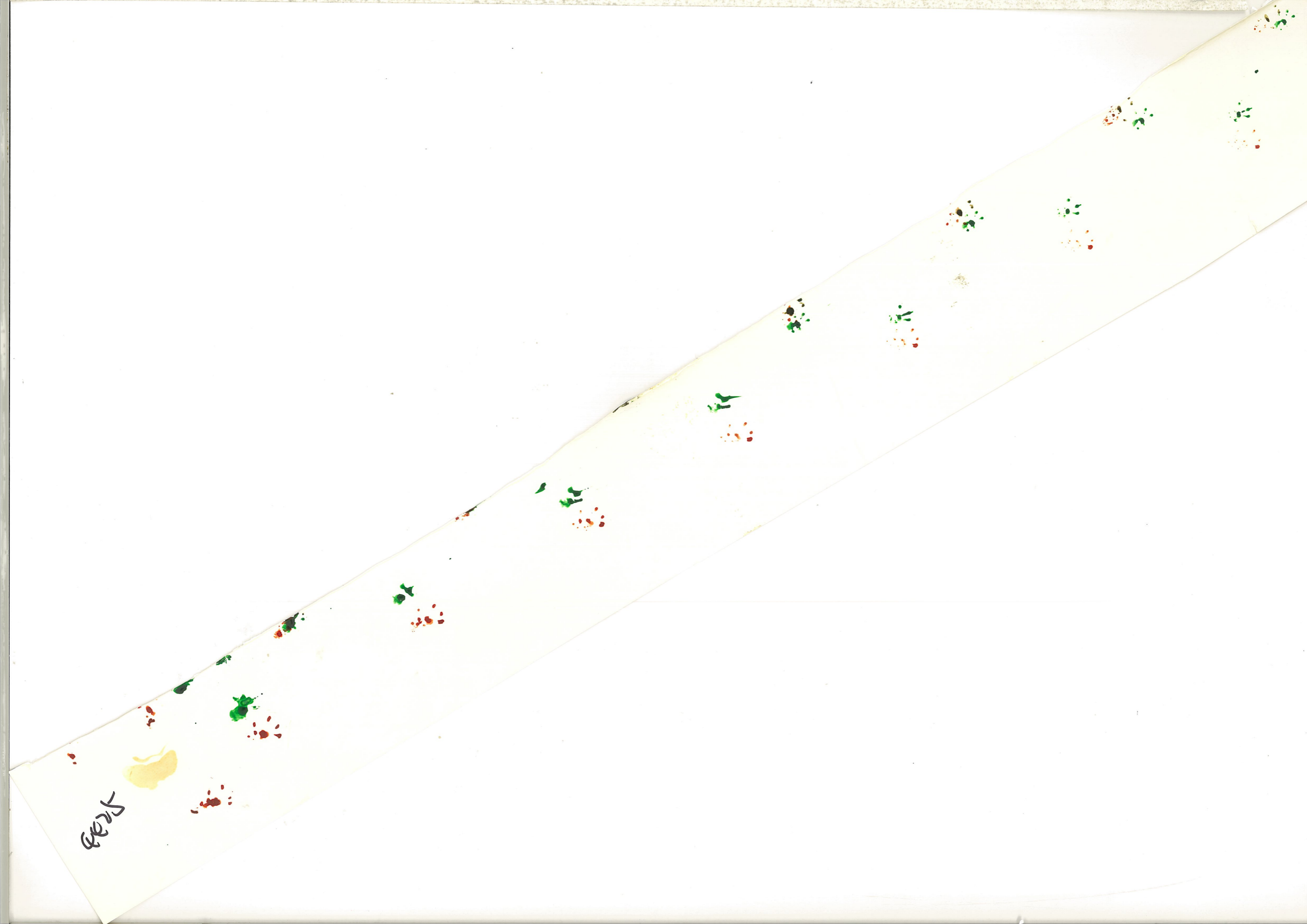

1333

4424

4413

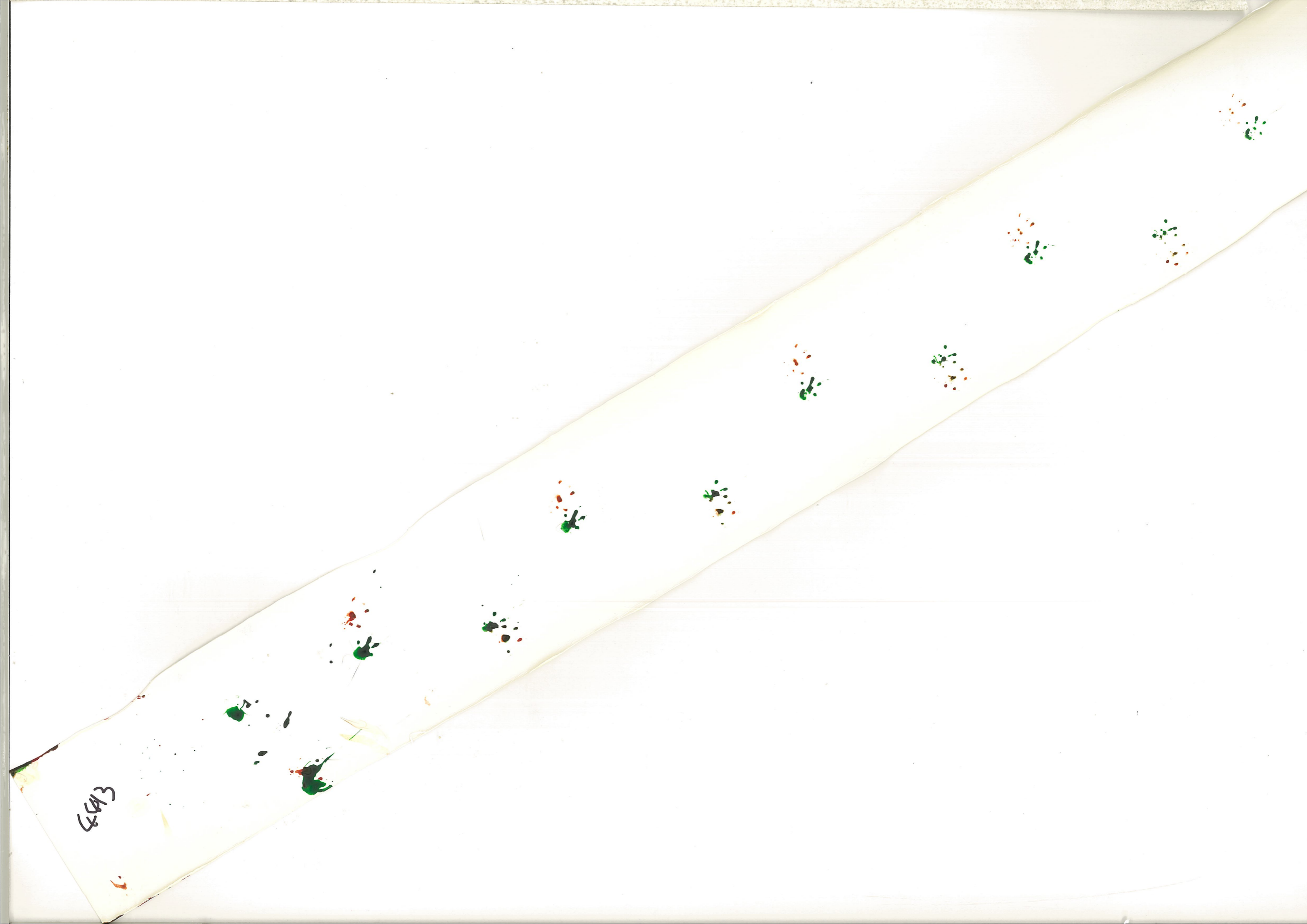

4422

4412

Supplement: Supplementary file 2 [file DataSheet2.zip › fig2/gait data.pdf]

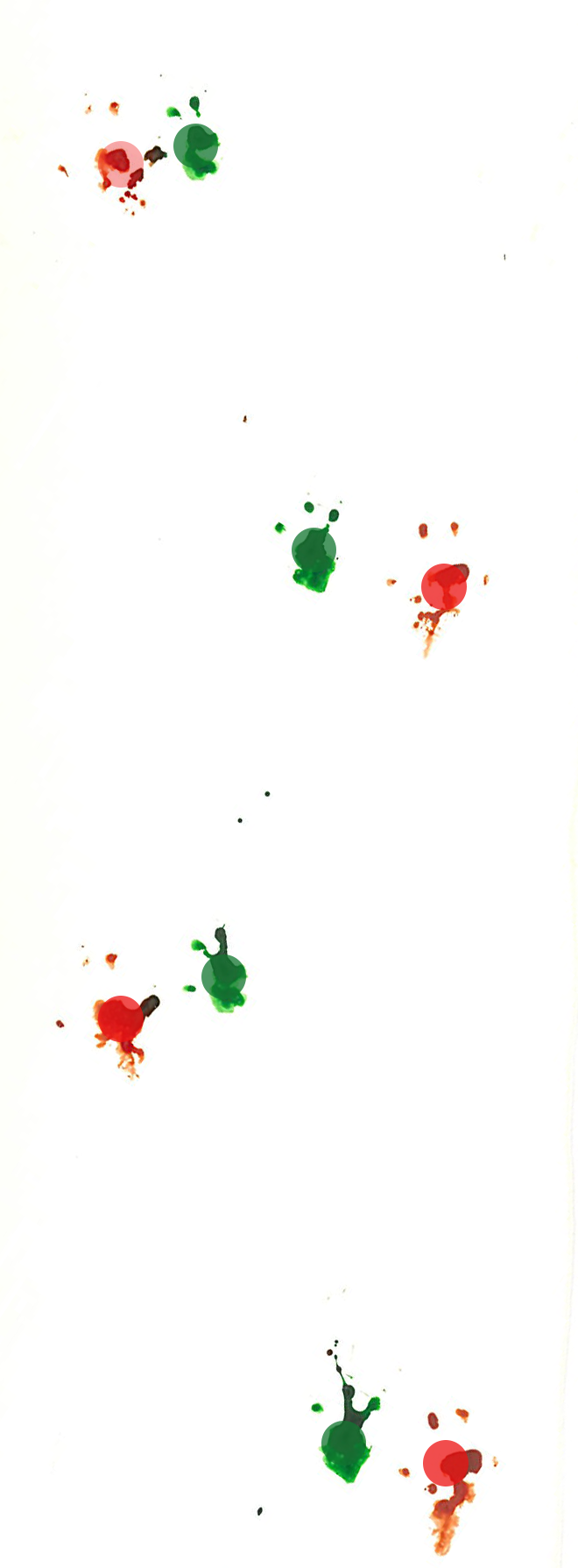

Supplement: Supplementary file 2 [file DataSheet2.zip › fig2/6.tif]

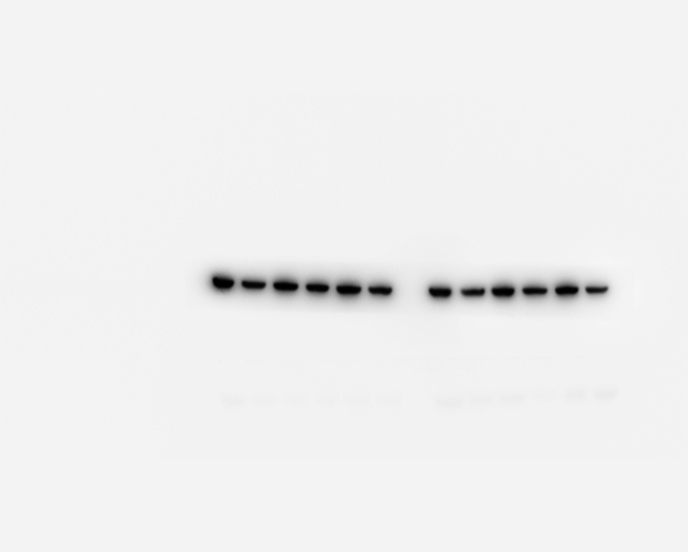

Supplement: Supplementary file 3 [file DataSheet3.zip › fig4/Western blot/CB protein expression in 12-week age mice.tif]

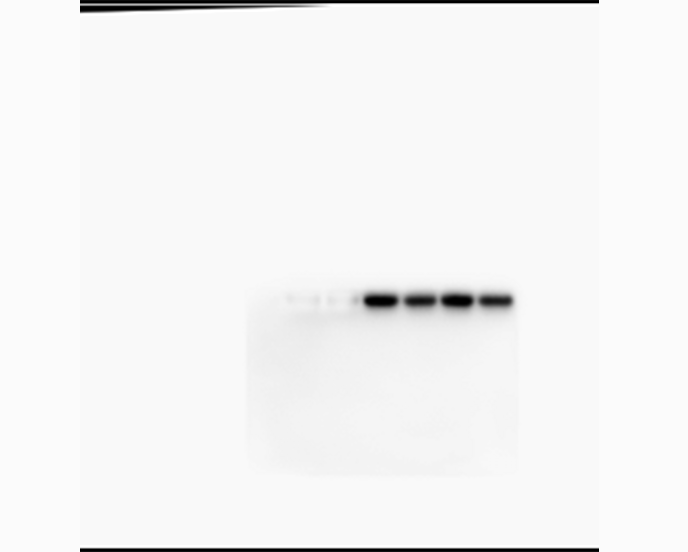

Supplement: Supplementary file 3 [file DataSheet3.zip › fig4/Western blot/CB protein expression in 20-week age mice.tif]

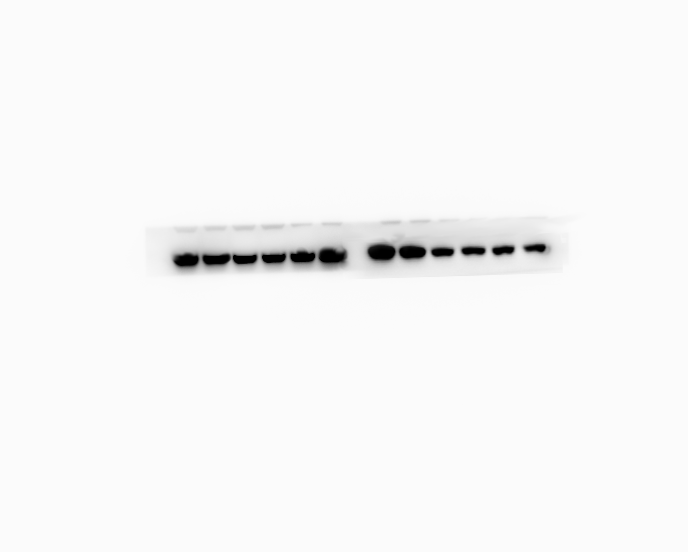

Supplement: Supplementary file 3 [file DataSheet3.zip › fig4/Western blot/GAPDH protein expression in 12-week age mice.tif]

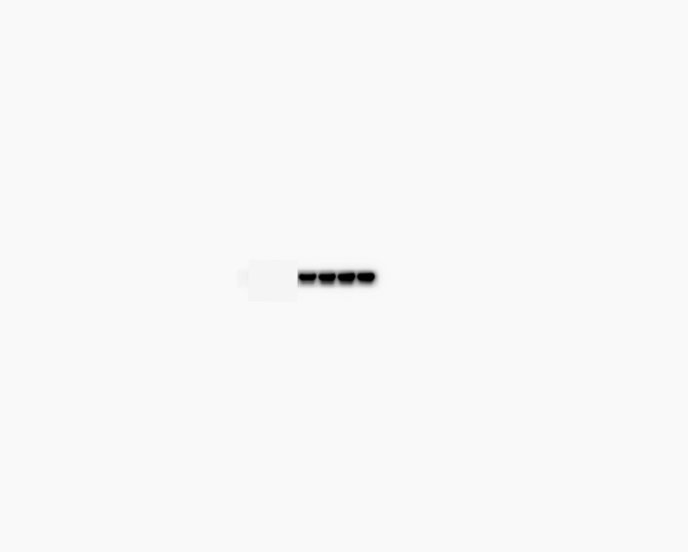

Supplement: Supplementary file 3 [file DataSheet3.zip › fig4/Western blot/GAPDH protein expression in 20-week age mice.tif]

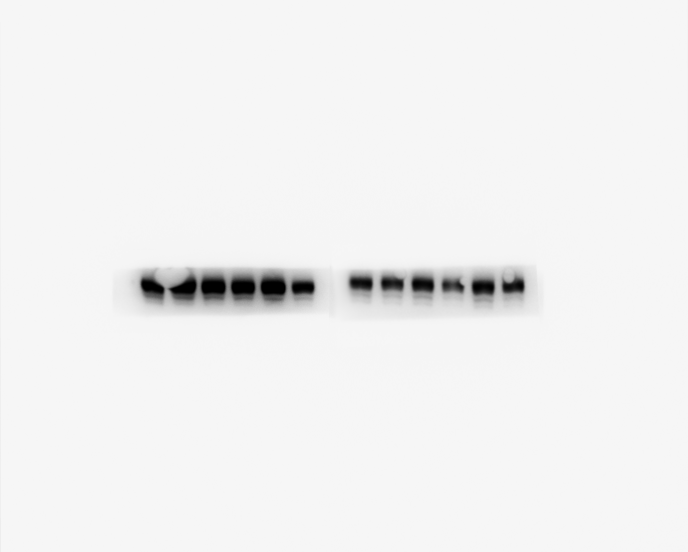

Supplement: Supplementary file 3 [file DataSheet3.zip › fig4/Western blot/PSD95 protein expression in 12-week age mice.tif]

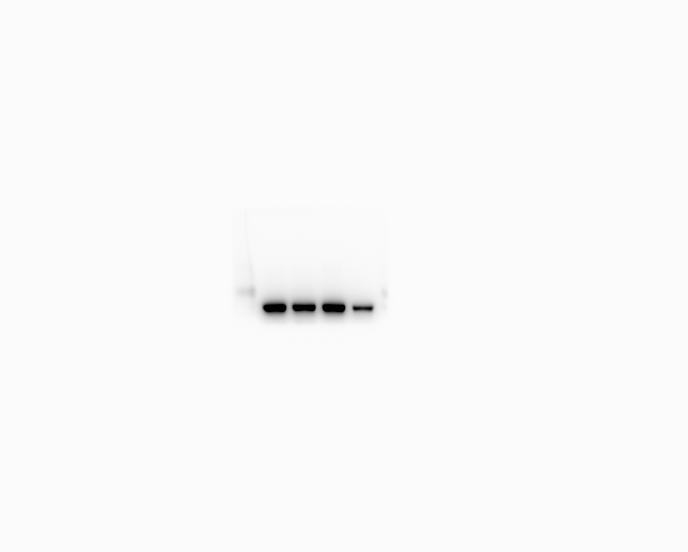

Supplement: Supplementary file 3 [file DataSheet3.zip › fig4/Western blot/PSD95 protein expression in 20 week age mice.Tif]

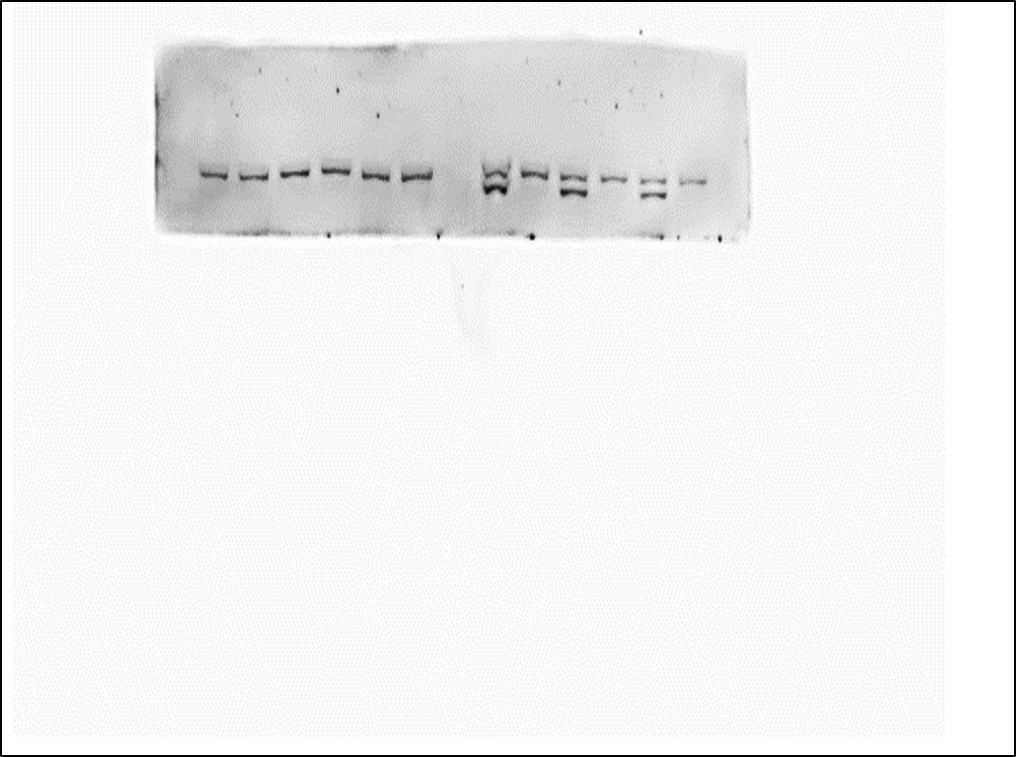

Supplement: Supplementary file 3 [file DataSheet3.zip › fig4/Western blot/Trio protein expression in 12 week age mice.tif]

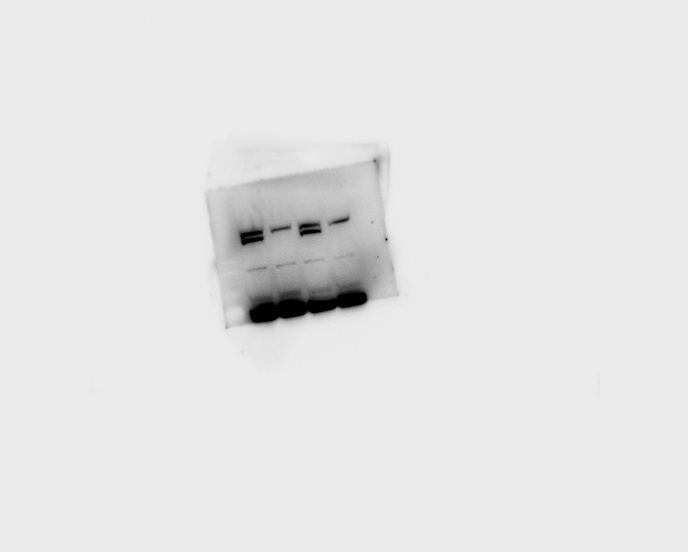

Supplement: Supplementary file 3 [file DataSheet3.zip › fig4/Western blot/Trio protein expression in 20 week age mice.Tif]

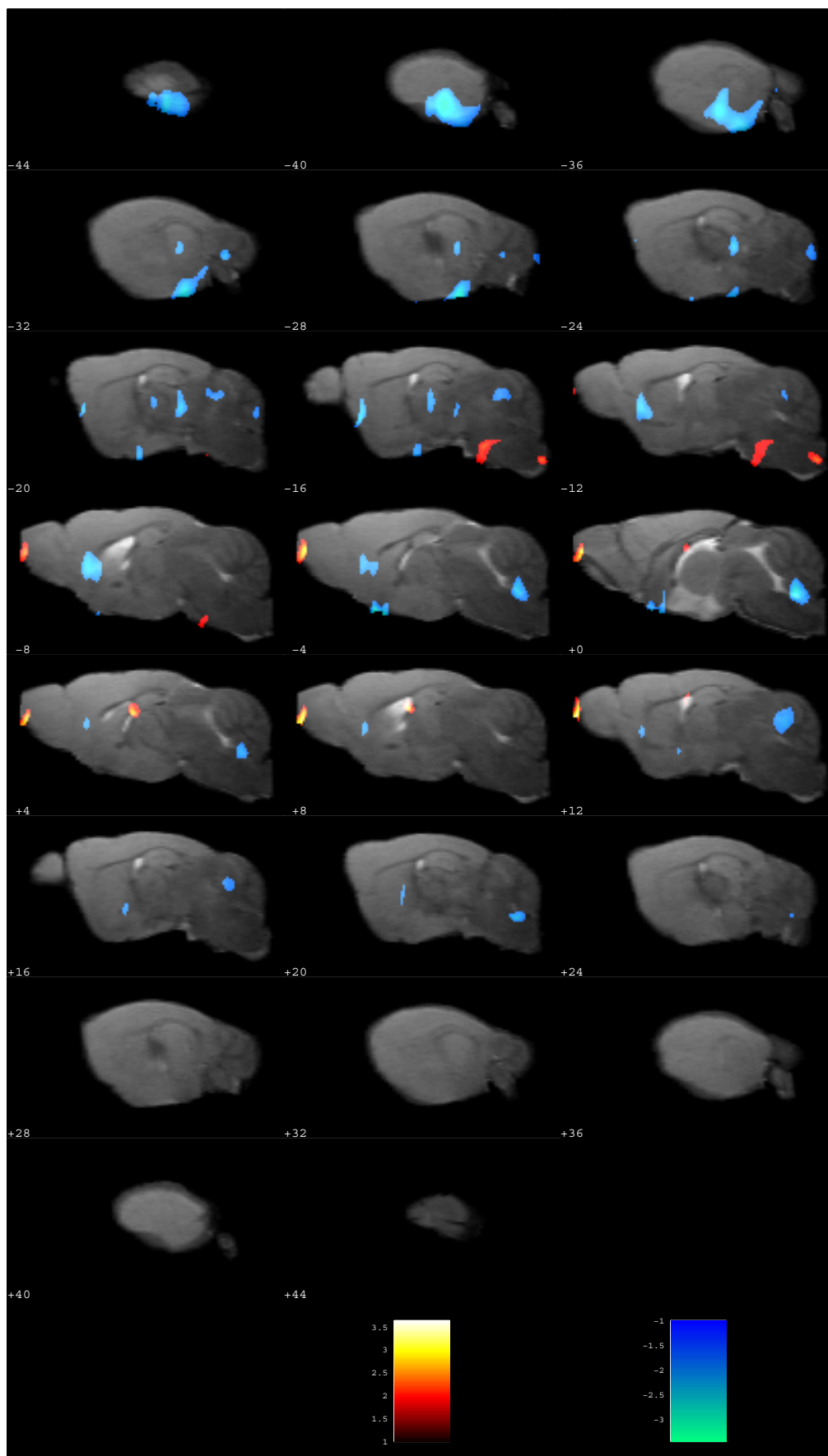

Supplement: Supplementary file 4 [file DataSheet4.zip › fig5/1_KO-WT_0.05-100_sagittal_big.pdf]

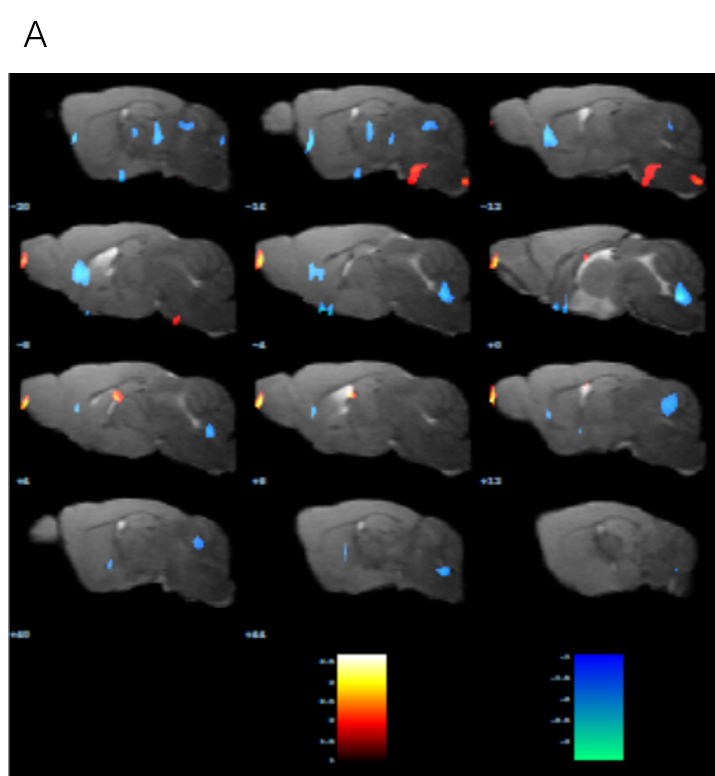

Supplement: Supplementary file 4 [file DataSheet4.zip › fig5/fig6.tif]

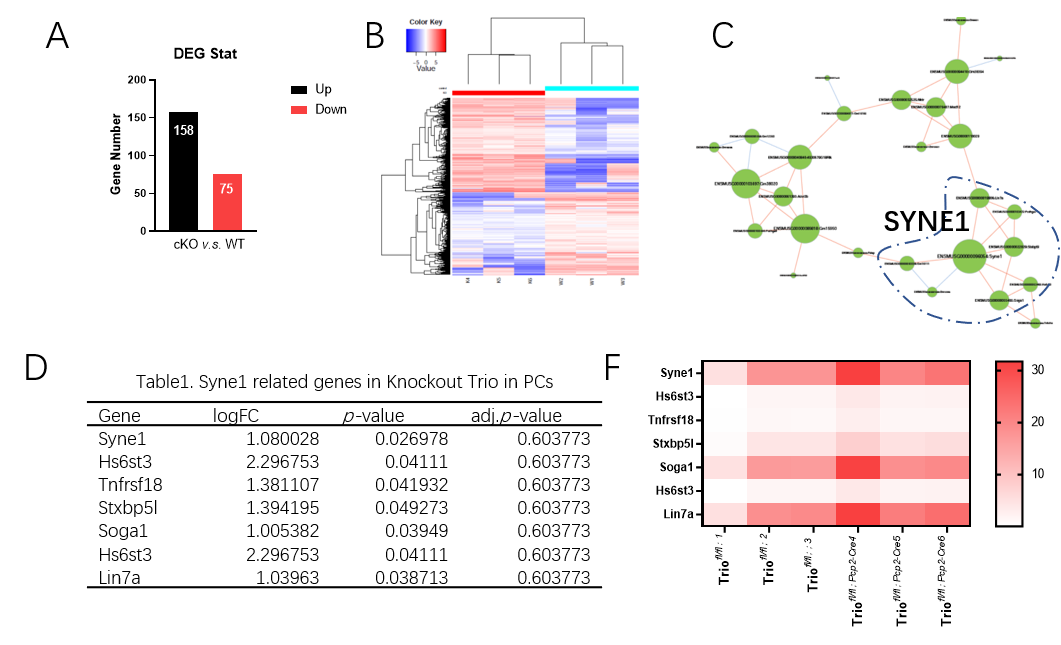

Supplement: Supplementary file 5 [file DataSheet5.zip › fig7/fig7.tif]
