# Supplementary material for: Deficiency of autism susceptibility gene Trio in cerebellar Purkinje cells leads to delayed motor impairments
Source: Front Psychiatry. 2025 Apr 10;15:1396716. doi: 10.3389/fpsyt.2024.1396716 (PMC12018246; doi:10.3389/fpsyt.2024.1396716)
Supplement: Supplementary file 4 [file DataSheet4.zip › fig5/1_result_KO-WT.pdf]

KO-WT

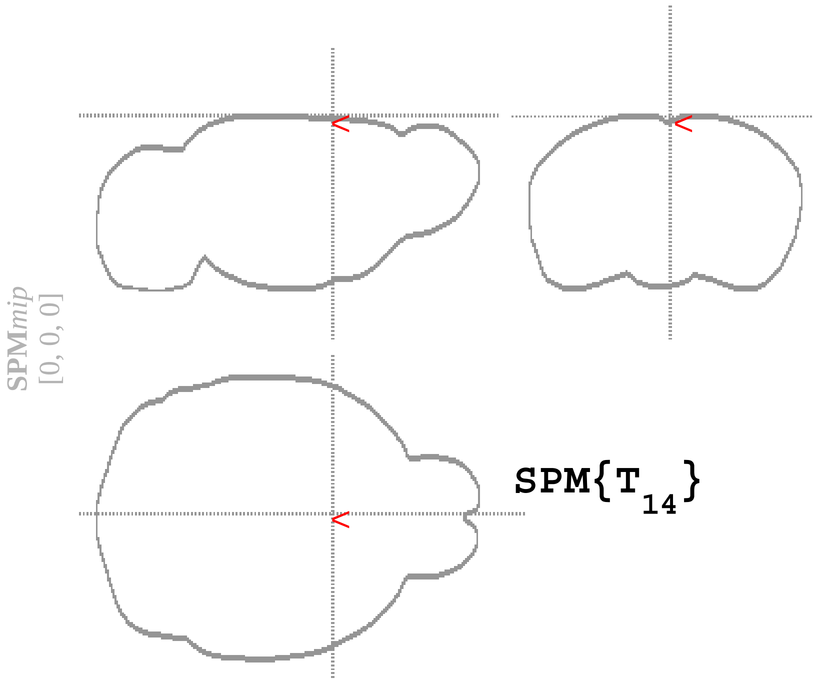

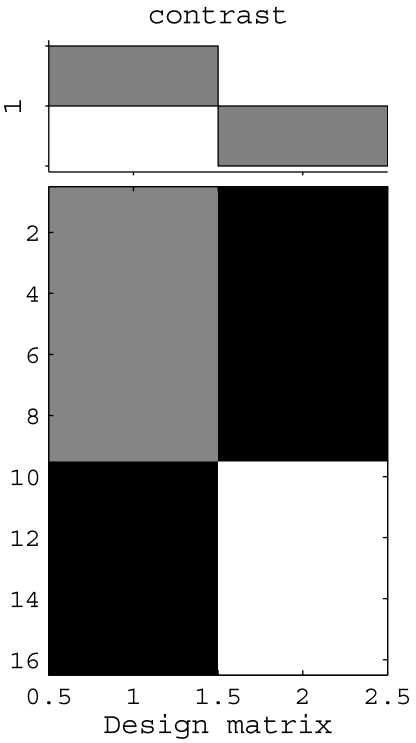

SPMresults: \Gray\result_KO-WT
Height threshold T = 3.787390 {p<0.001 (unc.)}
Extent threshold k = 100 voxels

Statistics: ***p-values adjusted for search volume***

set-level		cluster-level				peak-level					mm mm mm
p	c	$p_{FWE-corr}$	$q_{FDR-corr}$	k_E	p_{uncorr}	$p_{FWE-corr}$	$q_{FDR-corr}$	T	$(Z_\equiv)$	p_{uncorr}	

no suprathreshold clusters

table shows 3 local maxima more than 8.0mm apart

Height threshold: T = 3.79, p = 0.001 (0.827)
Extent threshold: k = 100 voxels, p = 0.566 (0.630)
Expected voxels per cluster, <k> = 309.612
Expected number of clusters, <c> = 0.99
FWEp: 6.527, FDRp: Inf, FWEc: Inf, FDRc: Inf

Degrees of freedom = [1.0, 14.0]
FWHM = 20.5 12.5 17.8 mm mm mm; 20.5 12.5 17.8 {vo
Volume: 377492 = 377492 voxels = 78.1 resels
Voxel size: 1.0 1.0 1.0 mm mm mm; (resel = 4551.86

WT-KO

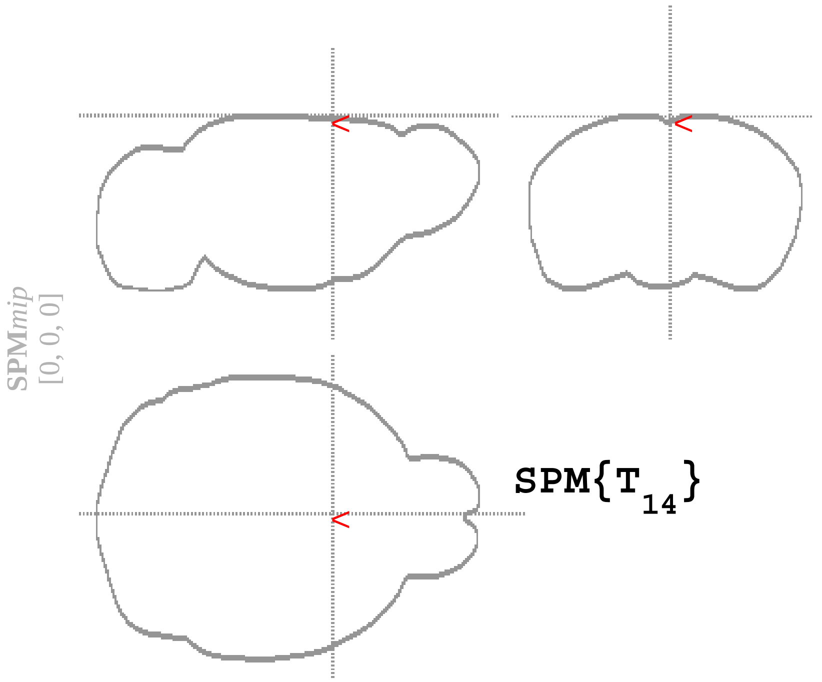

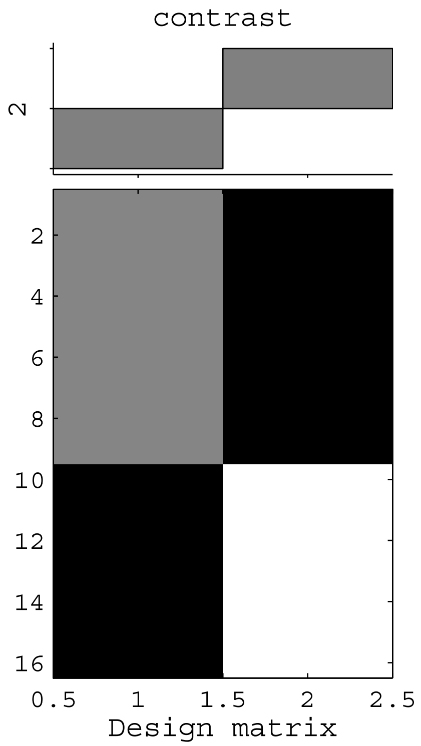

SPMresults: \Gray\result_KO-WT
Height threshold T = 3.787390 {p<0.001 (unc.)}
Extent threshold k = 100 voxels

Statistics: ***p-values adjusted for search volume***

set-level		cluster-level				peak-level					mm mm mm
p	*c*	$p_{FWE-corr}$	$q_{FDR-corr}$	k_E	p_{uncorr}	$p_{FWE-corr}$	$q_{FDR-corr}$	*T*	$(Z_{\equiv})$	p_{uncorr}	

no suprathreshold clusters

table shows 3 local maxima more than 8.0mm apart

Height threshold: T = 3.79, p = 0.001 (0.827)
Extent threshold: k = 100 voxels, p = 0.566 (0.630)
Expected voxels per cluster, <k> = 309.612
Expected number of clusters, <c> = 0.99
FWEp: 6.527, FDRp: Inf, FWEc: Inf, FDRc: Inf

Degrees of freedom = [1.0, 14.0]
FWHM = 20.5 12.5 17.8 mm mm mm; 20.5 12.5 17.8 {vo[illegible]
Volume: 377492 = 377492 voxels = 78.1 resels
Voxel size: 1.0 1.0 1.0 mm mm mm; (resel = 4551.86

KO-WT

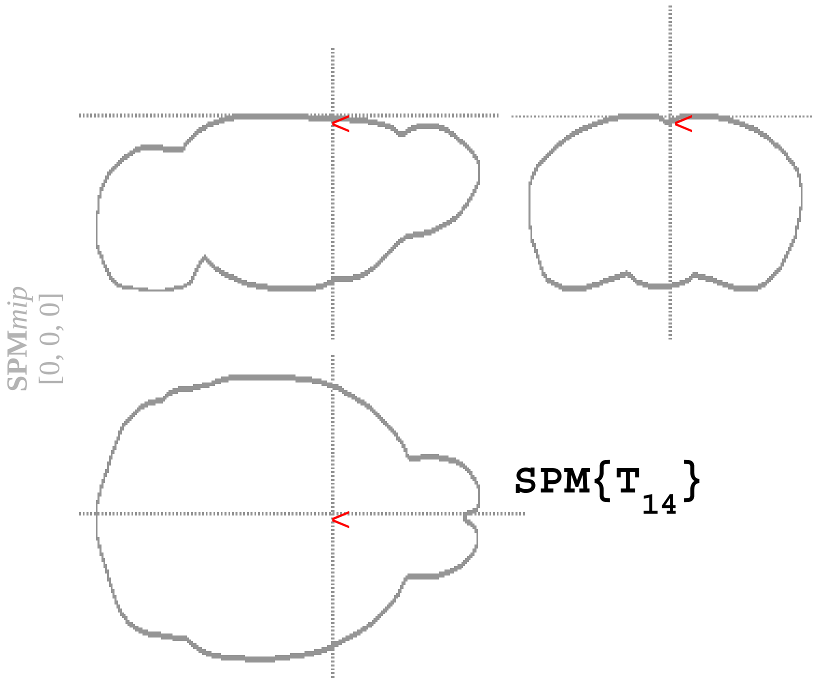

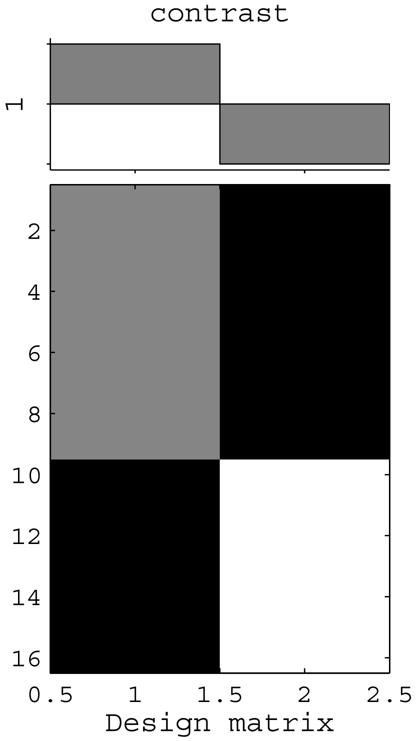

SPMresults: \Gray\result_KO-WT
Height threshold T = 3.787390 {p<0.001 (unc.)}
Extent threshold k = 50 voxels

Statistics: ***p-values adjusted for search volume***

set-level		cluster-level				peak-level					mm mm mm
p	c	$p_{FWE-corr}$	$q_{FDR-corr}$	k_E	p_{uncorr}	$p_{FWE-corr}$	$q_{FDR-corr}$	T	$(Z_{\equiv})$	p_{uncorr}	

no suprathreshold clusters

table shows 3 local maxima more than 8.0mm apart

Height threshold: T = 3.79, p = 0.001 (0.827)
Extent threshold: k = 50 voxels, p = 0.699 (0.707)
Expected voxels per cluster, <k> = 309.612
Expected number of clusters, <c> = 1.23
FWEp: 6.527, FDRp: Inf, FWEc: Inf, FDRc: Inf

Degrees of freedom = [1.0, 14.0]
FWHM = 20.5 12.5 17.8 mm mm mm; 20.5 12.5 17.8 {vo
Volume: 377492 = 377492 voxels = 78.1 resels
Voxel size: 1.0 1.0 1.0 mm mm mm; (resel = 4551.86

WT-KO

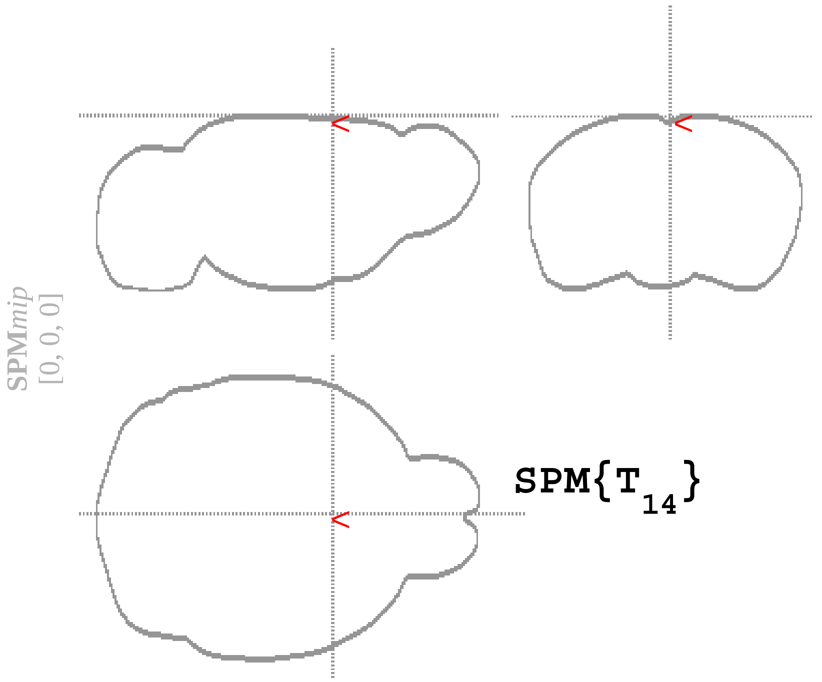

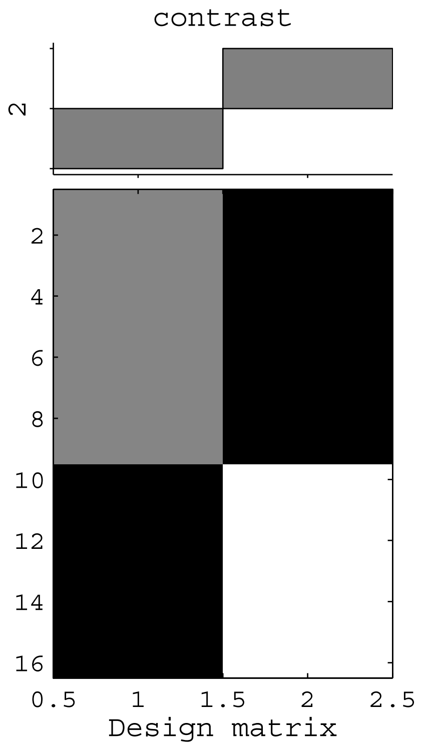

SPMresults: \Gray\result_KO-WT
Height threshold T = 3.787390 {p<0.001 (unc.)}
Extent threshold k = 50 voxels

Statistics: ***p-values adjusted for search volume***

set-level		cluster-level				peak-level					mm mm mm
p	c	$p_{FWE-corr}$	$q_{FDR-corr}$	k_E	p_{uncorr}	$p_{FWE-corr}$	$q_{FDR-corr}$	T	$(Z_{\equiv})$	p_{uncorr}	

no suprathreshold clusters

table shows 3 local maxima more than 8.0mm apart

Height threshold: T = 3.79, p = 0.001 (0.827) Degrees of freedom = [1.0, 14.0]
Extent threshold: k = 50 voxels, p = 0.699 (0.707) FWHM = 20.5 12.5 17.8 mm mm mm; 20.5 12.5 17.8 {vo
Expected voxels per cluster, <k> = 309.612 Volume: 377492 = 377492 voxels = 78.1 resels
Expected number of clusters, <c> = 1.23 Voxel size: 1.0 1.0 1.0 mm mm mm; (resel = 4551.86
FWEp: 6.527, FDRp: Inf, FWEc: Inf, FDRc: Inf

KO-WT

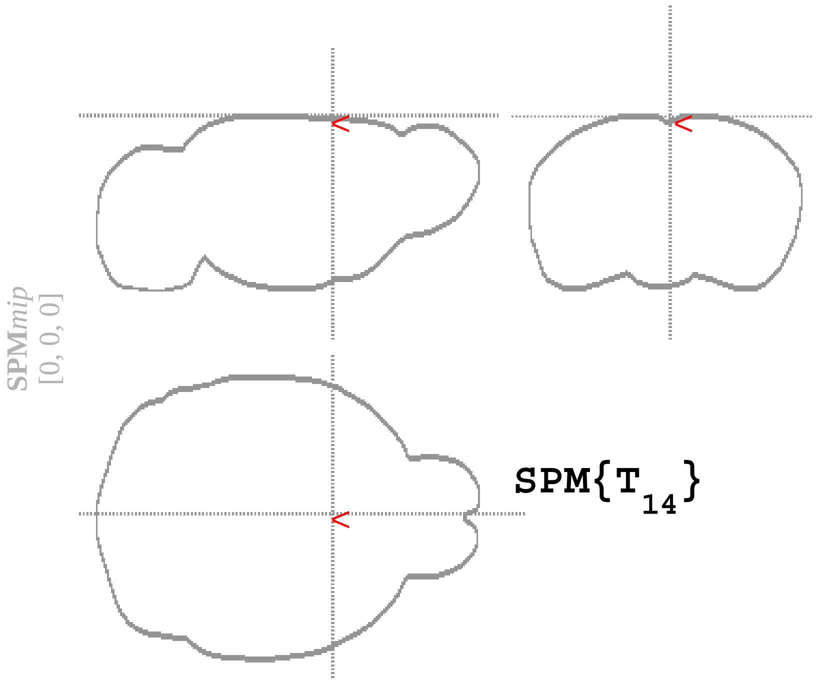

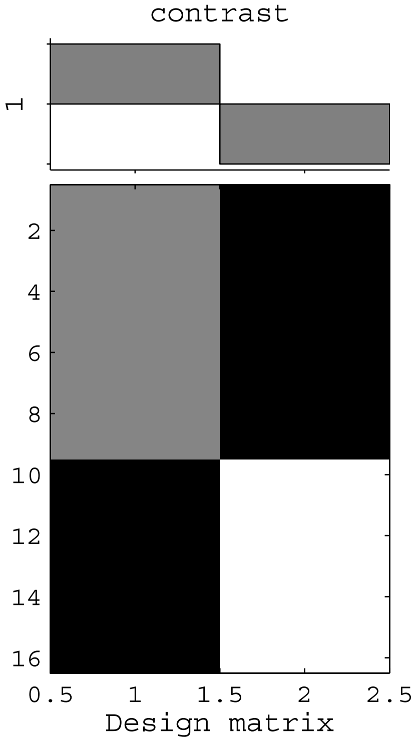

SPMresults: \Gray\result_KO-WT
Height threshold T = 3.787390 {p<0.001 (unc.)}
Extent threshold k = 20 voxels

Statistics: ***p-values adjusted for search volume***

set-level		cluster-level				peak-level					mm mm mm
p	c	$p_{FWE-corr}$	$q_{FDR-corr}$	k_E	p_{uncorr}	$p_{FWE-corr}$	$q_{FDR-corr}$	T	$(Z_{\equiv})$	p_{uncorr}	

no suprathreshold clusters

table shows 3 local maxima more than 8.0mm apart

Height threshold: T = 3.79, p = 0.001 (0.827) Degrees of freedom = [1.0, 14.0]
Extent threshold: k = 20 voxels, p = 0.823 (0.765) FWHM = 20.5 12.5 17.8 mm mm mm; 20.5 12.5 17.8 {vo
Expected voxels per cluster, <k> = 309.612 Volume: 377492 = 377492 voxels = 78.1 resels
Expected number of clusters, <c> = 1.45 Voxel size: 1.0 1.0 1.0 mm mm mm; (resel = 4551.86
FWEp: 6.527, FDRp: Inf, FWEc: Inf, FDRc: Inf

WT-KO

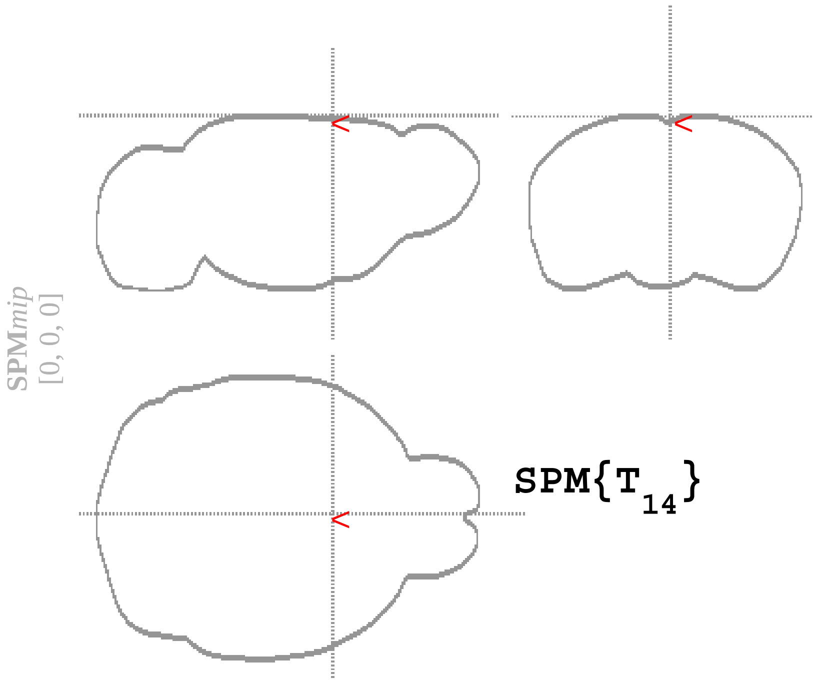

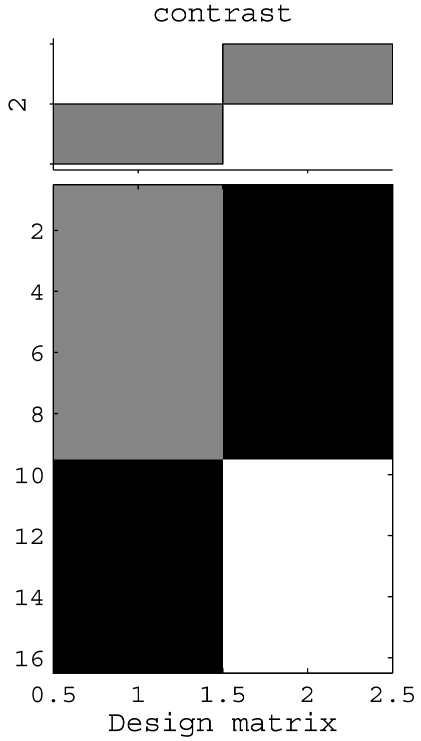

SPMresults: \Gray\result_KO-WT
Height threshold T = 3.787390 {p<0.001 (unc.)}
Extent threshold k = 20 voxels

Statistics: ***p-values adjusted for search volume***

set-level		cluster-level				peak-level					mm mm mm
p	c	$p_{FWE-corr}$	$q_{FDR-corr}$	k_E	p_{uncorr}	$p_{FWE-corr}$	$q_{FDR-corr}$	T	$(Z_{\equiv})$	p_{uncorr}	

no suprathreshold clusters

table shows 3 local maxima more than 8.0mm apart

Height threshold: T = 3.79, p = 0.001 (0.827)
Extent threshold: k = 20 voxels, p = 0.823 (0.765)
Expected voxels per cluster, <k> = 309.612
Expected number of clusters, <c> = 1.45
FWEp: 6.527, FDRp: Inf, FWEc: Inf, FDRc: Inf

Degrees of freedom = [1.0, 14.0]
FWHM = 20.5 12.5 17.8 mm mm mm; 20.5 12.5 17.8 {vo
Volume: 377492 = 377492 voxels = 78.1 resels
Voxel size: 1.0 1.0 1.0 mm mm mm; (resel = 4551.86

KO-WT

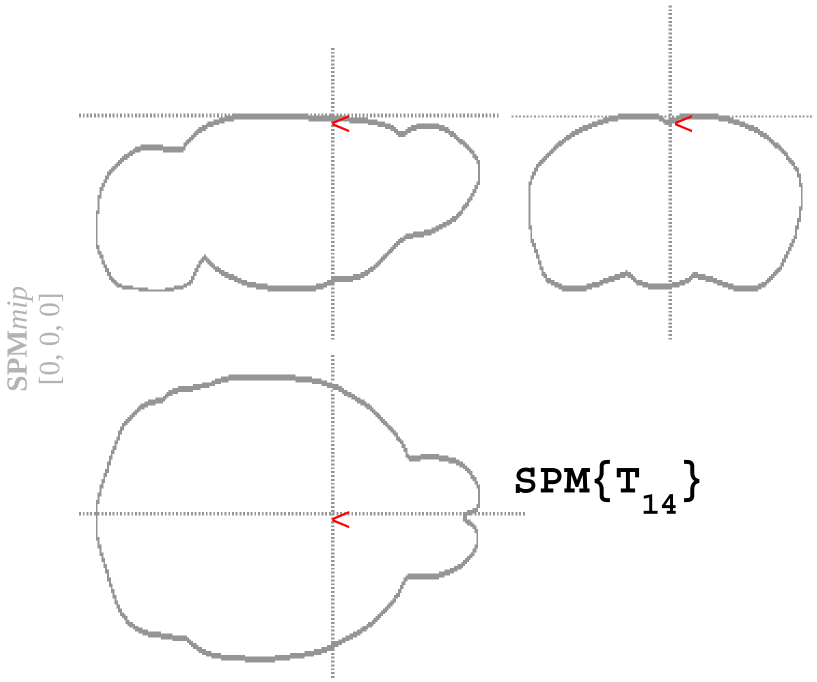

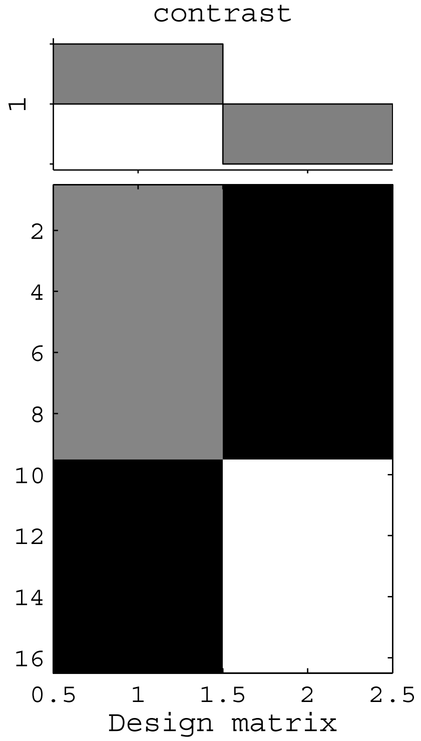

SPMresults: \Gray\result_KO-WT
Height threshold T = 2.976843 {p<0.005 (unc.)}
Extent threshold k = 100 voxels

Statistics: ***p-values adjusted for search volume***

set-level		cluster-level				peak-level					mm mm mm
p	c	$p_{FWE-corr}$	$q_{FDR-corr}$	k_E	p_{uncorr}	$p_{FWE-corr}$	$q_{FDR-corr}$	T	$(Z_{\equiv})$	p_{uncorr}	

no suprathreshold clusters

table shows 3 local maxima more than 8.0mm apart

Height threshold: T = 2.98, p = 0.005 (0.991) Degrees of freedom = [1.0, 14.0]
Extent threshold: k = 100 voxels, p = 0.707 (0.964) FWHM = 20.5 12.5 17.8 mm mm mm; 20.5 12.5 17.8 {vo[illegible]
Expected voxels per cluster, <k> = 652.372 Volume: 377492 = 377492 voxels = 78.1 resels
Expected number of clusters, <c> = 3.33 Voxel size: 1.0 1.0 1.0 mm mm mm; (resel = 4551.86
FWEp: 6.527, FDRp: Inf, FWEc: Inf, FDRc: Inf

WT-KO

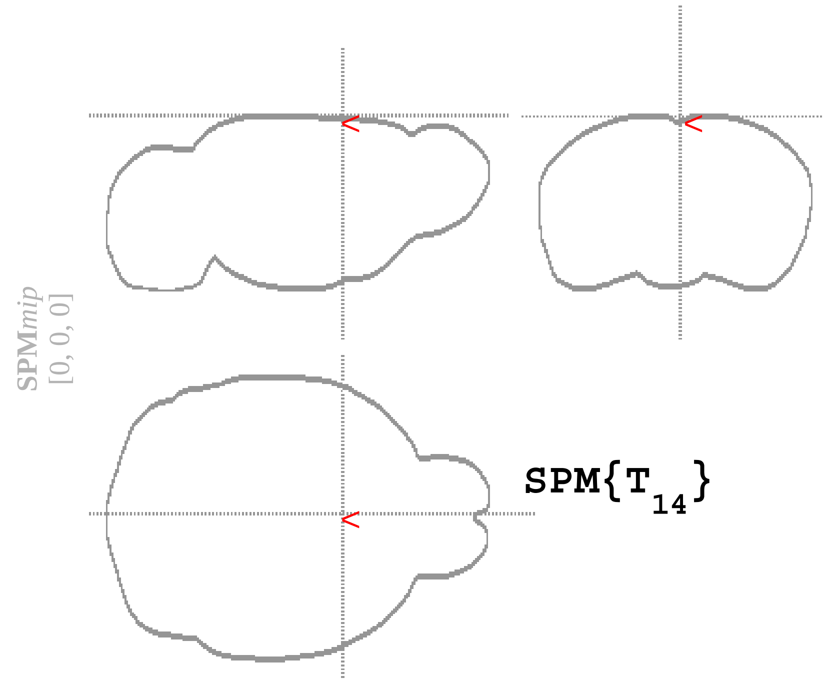

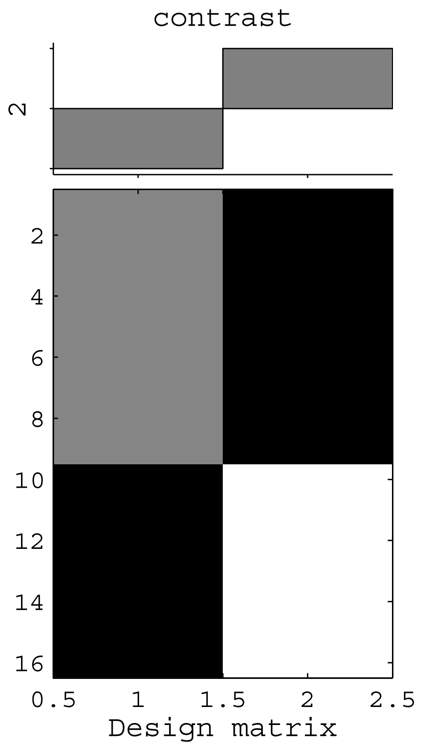

SPMresults: \Gray\result_KO-WT
Height threshold T = 2.976843 {p<0.005 (unc.)}
Extent threshold k = 100 voxels

Statistics: ***p-values adjusted for search volume***

set-level		cluster-level				peak-level					mm mm mm
p	c	$p_{FWE-corr}$	$q_{FDR-corr}$	k_E	p_{uncorr}	$p_{FWE-corr}$	$q_{FDR-corr}$	T	$(Z_{\equiv})$	p_{uncorr}	

no suprathreshold clusters

table shows 3 local maxima more than 8.0mm apart

Height threshold: T = 2.98, p = 0.005 (0.991)
Extent threshold: k = 100 voxels, p = 0.707 (0.964)
Expected voxels per cluster, <k> = 652.372
Expected number of clusters, <c> = 3.33
FWEp: 6.527, FDRp: Inf, FWEc: Inf, FDRc: Inf

Degrees of freedom = [1.0, 14.0]
FWHM = 20.5 12.5 17.8 mm mm mm; 20.5 12.5 17.8 {vo
Volume: 377492 = 377492 voxels = 78.1 resels
Voxel size: 1.0 1.0 1.0 mm mm mm; (resel = 4551.86

KO-WT

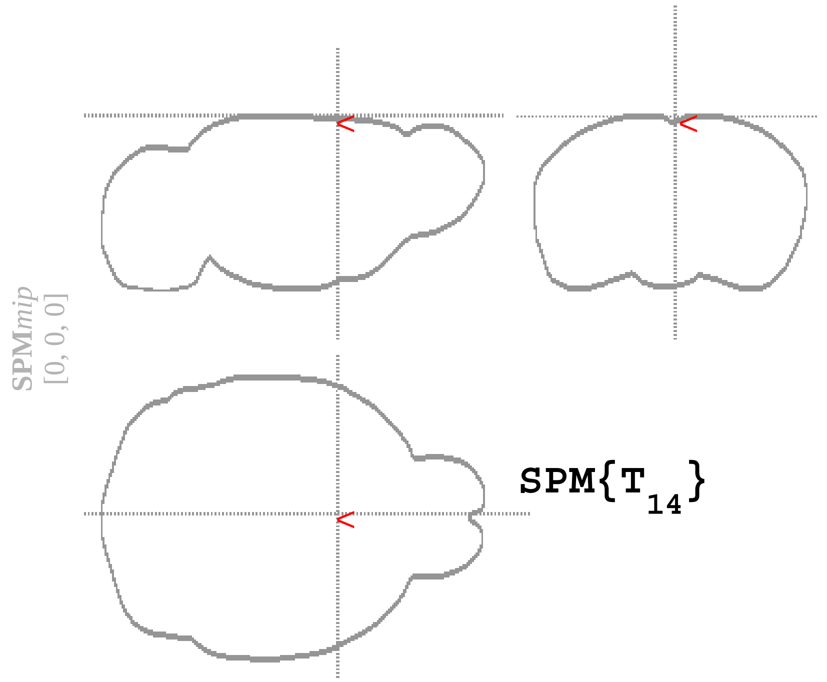

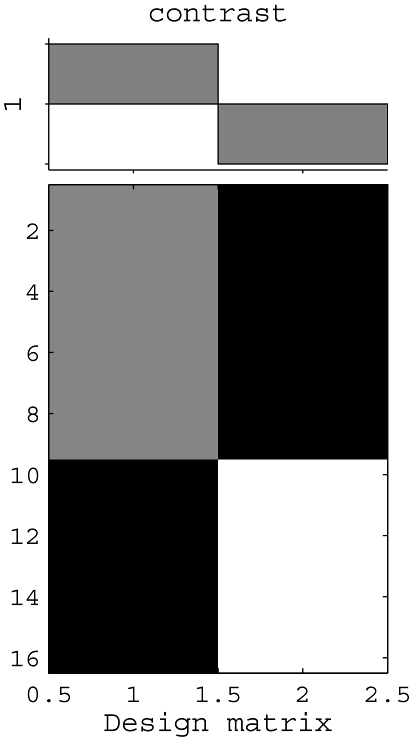

SPMresults: \Gray\result_KO-WT
Height threshold T = 2.976843 {p<0.005 (unc.)}
Extent threshold k = 50 voxels

Statistics: ***p-values adjusted for search volume***

set-level		cluster-level				peak-level					
p	c	$p_{FWE-corr}$	$q_{FDR-corr}$	k_E	p_{uncorr}	$p_{FWE-corr}$	$q_{FDR-corr}$	T	$(Z_{\equiv})$	p_{uncorr}	mm mm mm

no suprathreshold clusters

table shows 3 local maxima more than 8.0mm apart

Height threshold: T = 2.98, p = 0.005 (0.991) Degrees of freedom = [1.0, 14.0]
Extent threshold: k = 50 voxels, p = 0.804 (0.977) FWHM = 20.5 12.5 17.8 mm mm mm; 20.5 12.5 17.8 {vo
Expected voxels per cluster, <k> = 652.372 Volume: 377492 = 377492 voxels = 78.1 resels
Expected number of clusters, <c> = 3.79 Voxel size: 1.0 1.0 1.0 mm mm mm; (resel = 4551.86
FWEp: 6.527, FDRp: Inf, FWEc: Inf, FDRc: Inf

WT-KO

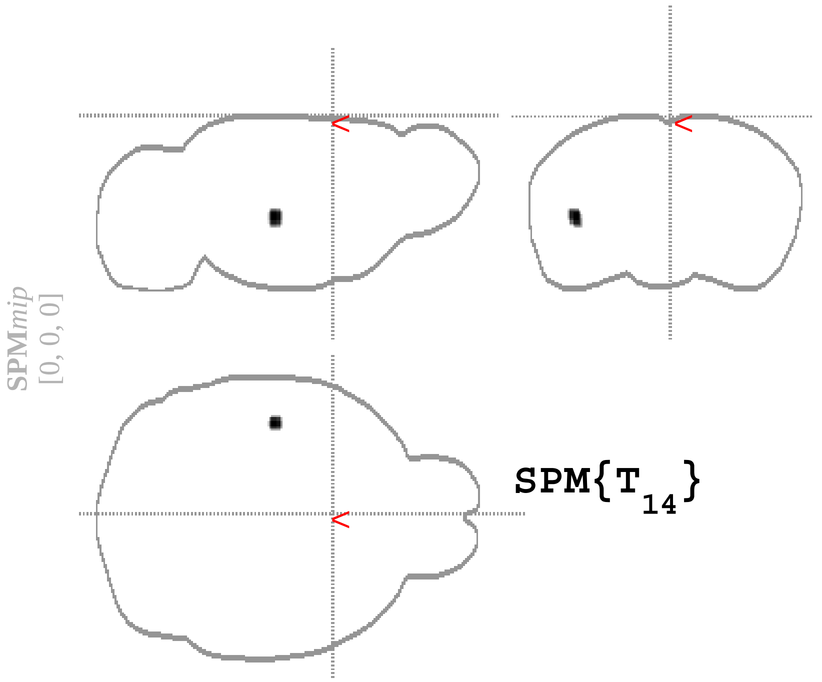

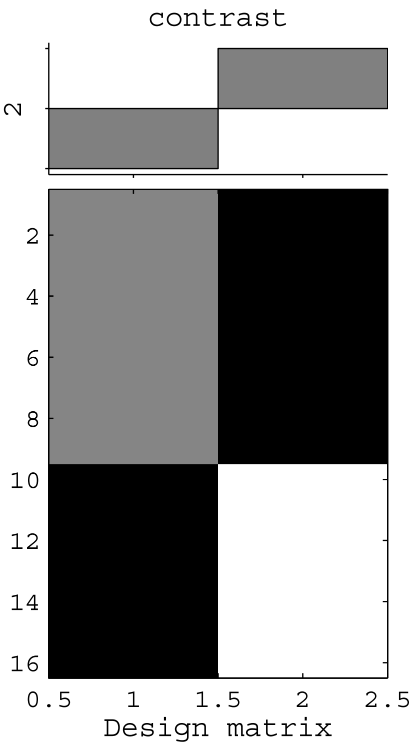

SPMresults: \Gray\result_KO-WT
Height threshold T = 2.976843 {p<0.005 (unc.)}
Extent threshold k = 50 voxels

Statistics: ***p-values adjusted for search volume***

cluster-level				peak-level							
$p_{FWE-corr}$	$q_{FDR-corr}$	k_E	p_{uncorr}	$p_{FWE-corr}$	$q_{FDR-corr}$	T	$(Z_{\equiv})$	p_{uncorr}	mm	mm	mm
0.968	0.729	87	0.729	0.934	0.576	3.44	2.88	0.002	4	4	-2

table shows 3 local maxima more than 8.0mm apart

Height threshold: T = 2.98, p = 0.005 (0.991)
Extent threshold: k = 50 voxels, p = 0.804 (0.977)
Expected voxels per cluster, <k> = 652.372
Expected number of clusters, <c> = 3.79
FWEp: 6.527, FDRp: Inf, FWEc: Inf, FDRc: Inf

Degrees of freedom = [1.0, 14.0]
FWHM = 20.5 12.5 17.8 mm mm mm; 20.5 12.5 17.8 {vo
Volume: 377492 = 377492 voxels = 78.1 resels
Voxel size: 1.0 1.0 1.0 mm mm mm; (resel = 4551.86

KO-WT

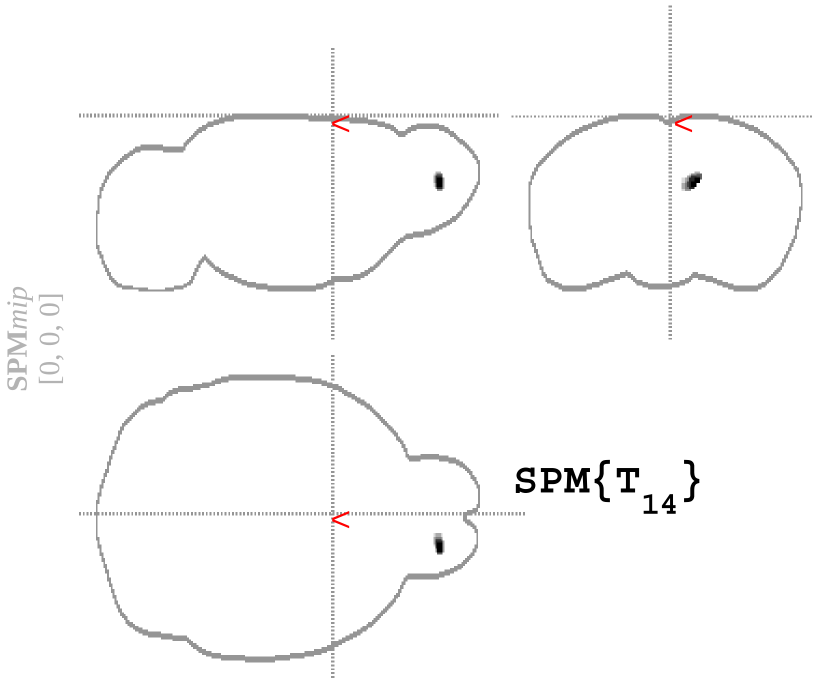

SPMresults: \Gray\result_KO-WT
Height threshold T = 2.976843 {p<0.005 (unc.)}
Extent threshold k = 20 voxels

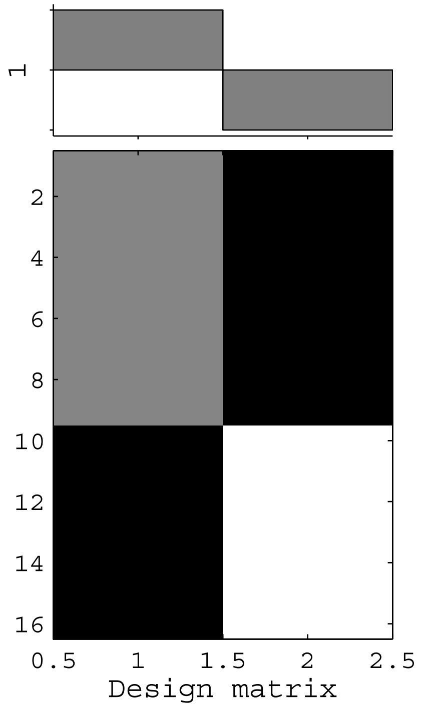

Statistics: ***p-values adjusted for search volume***

	cluster-level				peak-level							
	$p_{FWE-corr}$	$q_{FDR-corr}$	k_E	p_{uncorr}	$p_{FWE-corr}$	$q_{FDR-corr}$	T	$(Z_{\equiv})$	p_{uncorr}	mm	mm	mm
	0.979	0.823	42	0.823	0.878	0.529	3.65	3.01	0.001	-1	3	6

table shows 3 local maxima more than 8.0mm apart

Height threshold: T = 2.98, p = 0.005 (0.991) Degrees of freedom = [1.0, 14.0]
Extent threshold: k = 20 voxels, p = 0.888 (0.985) FWHM = 20.5 12.5 17.8 mm mm mm; 20.5 12.5 17.8 {vo
Expected voxels per cluster, <k> = 652.372 Volume: 377492 = 377492 voxels = 78.1 resels
Expected number of clusters, <c> = 4.19 Voxel size: 1.0 1.0 1.0 mm mm mm; (resel = 4551.86
FWEp: 6.527, FDRp: Inf, FWEc: Inf, FDRc: Inf

WT-KO

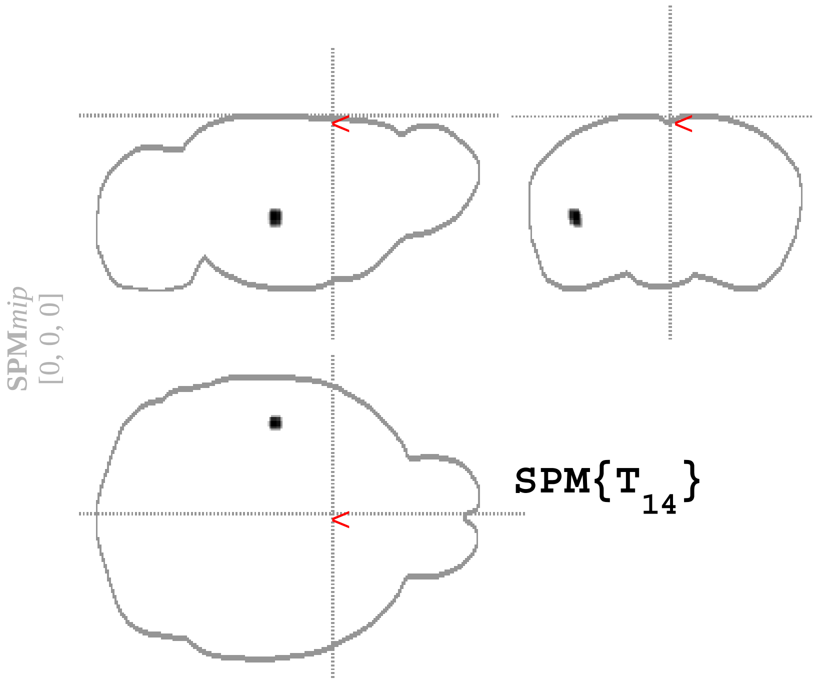

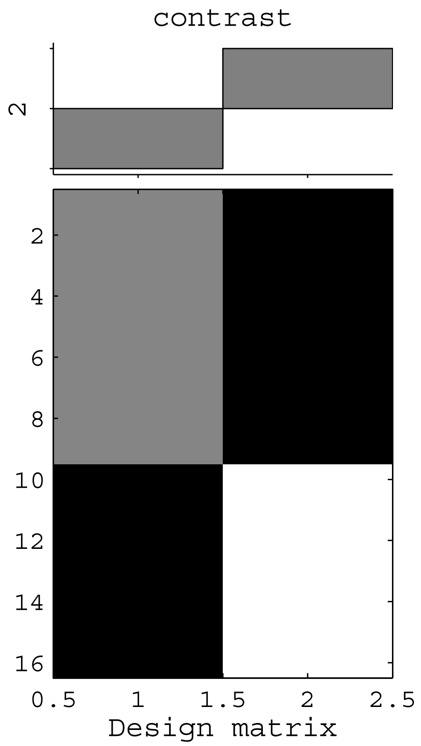

SPMresults: \Gray\result_KO-WT
Height threshold T = 2.976843 {p<0.005 (unc.)}
Extent threshold k = 20 voxels

Statistics: *p-values adjusted for search volume*

cluster-level				peak-level							
$p_{FWE-corr}$	$q_{FDR-corr}$	k_E	p_{uncorr}	$p_{FWE-corr}$	$q_{FDR-corr}$	T	$(Z_{\equiv})$	p_{uncorr}	mm	mm	mm
0.968	0.729	87	0.729	0.934	0.576	3.44	2.88	0.002	4	4	-2

table shows 3 local maxima more than 8.0mm apart

Height threshold: T = 2.98, p = 0.005 (0.991)
Extent threshold: k = 20 voxels, p = 0.888 (0.985)
Expected voxels per cluster, <k> = 652.372
Expected number of clusters, <c> = 4.19
FWEp: 6.527, FDRp: Inf, FWEc: Inf, FDRc: Inf

Degrees of freedom = [1.0, 14.0]
FWHM = 20.5 12.5 17.8 mm mm mm; 20.5 12.5 17.8 {vo
Volume: 377492 = 377492 voxels = 78.1 resels
Voxel size: 1.0 1.0 1.0 mm mm mm; (resel = 4551.86

KO-WT

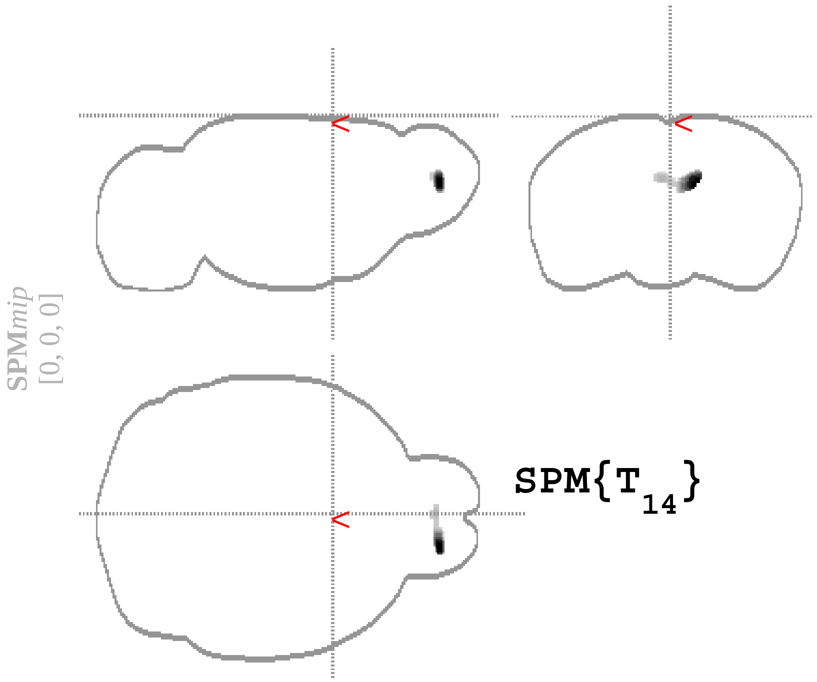

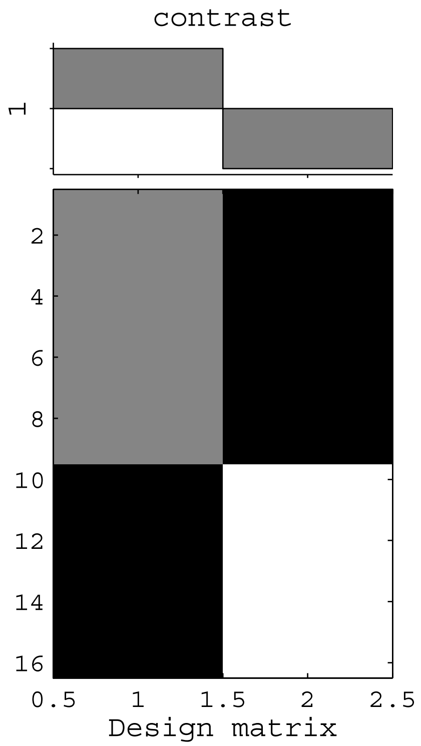

SPMresults: \Gray\result_KO-WT
Height threshold T = 2.624494 {p<0.01 (unc.)}
Extent threshold k = 100 voxels

Statistics: *p-values adjusted for search volume*

	cluster-level				peak-level							
	$p_{FWE-corr}$	$q_{FDR-corr}$	k_E	p_{uncorr}	$p_{FWE-corr}$	$q_{FDR-corr}$	T	$(Z_{\equiv})$	p_{uncorr}	mm	mm	mm
	**0.994**	**0.952**	**118**	**0.743**	**0.878**	**0.890**	**3.65**	**3.01**	**0.001**	**-1**	**3**	**6**
					0.997	0.890	2.81	2.46	0.007	0	3	6

table shows 3 local maxima more than 8.0mm apart

Height threshold: T = 2.62, p = 0.010 (0.999)
Extent threshold: k = 100 voxels, p = 0.767 (0.995)
Expected voxels per cluster, <k> = 971.951
Expected number of clusters, <c> = 5.27
FWEp: 6.527, FDRp: Inf, FWEc: Inf, FDRc: Inf

Degrees of freedom = [1.0, 14.0]
FWHM = 20.5 12.5 17.8 mm mm mm; 20.5 12.5 17.8 {vo
Volume: 377492 = 377492 voxels = 78.1 resels
Voxel size: 1.0 1.0 1.0 mm mm mm; (resel = 4551.86

WT-KO

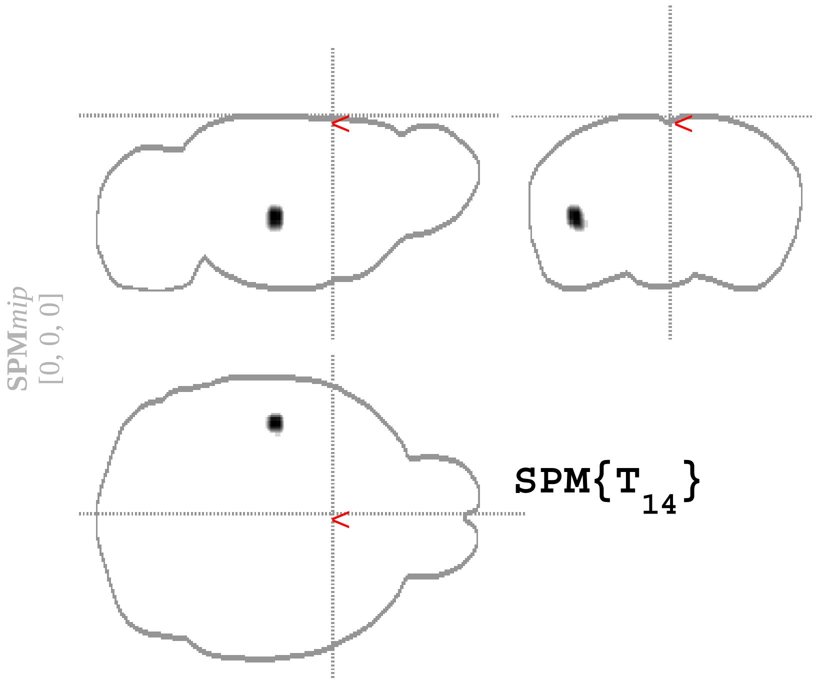

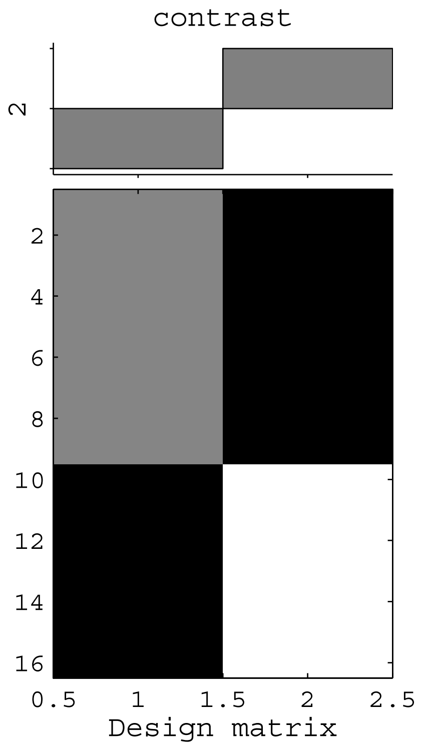

SPMresults: \Gray\result_KO-WT
Height threshold T = 2.624494 {p<0.01 (unc.)}
Extent threshold k = 100 voxels

Statistics: ***p-values adjusted for search volume***

	cluster-level				peak-level							
set-level	$p_{FWE-corr}$	$q_{FDR-corr}$	k_E	p_{uncorr}	$p_{FWE-corr}$	$q_{FDR-corr}$	T	$(Z_\equiv)$	p_{uncorr}	mm	mm	mm
	0.984	0.988	263	0.603	0.934	0.966	3.44	2.88	0.002	4	4	-2

table shows 3 local maxima more than 8.0mm apart

Height threshold: T = 2.62, p = 0.010 (0.999)
Extent threshold: k = 100 voxels, p = 0.767 (0.995)
Expected voxels per cluster, <k> = 971.951
Expected number of clusters, <c> = 5.27
FWEp: 6.527, FDRp: Inf, FWEc: Inf, FDRc: Inf

Degrees of freedom = [1.0, 14.0]
FWHM = 20.5 12.5 17.8 mm mm mm; 20.5 12.5 17.8 {vo
Volume: 377492 = 377492 voxels = 78.1 resels
Voxel size: 1.0 1.0 1.0 mm mm mm; (resel = 4551.86

KO-WT

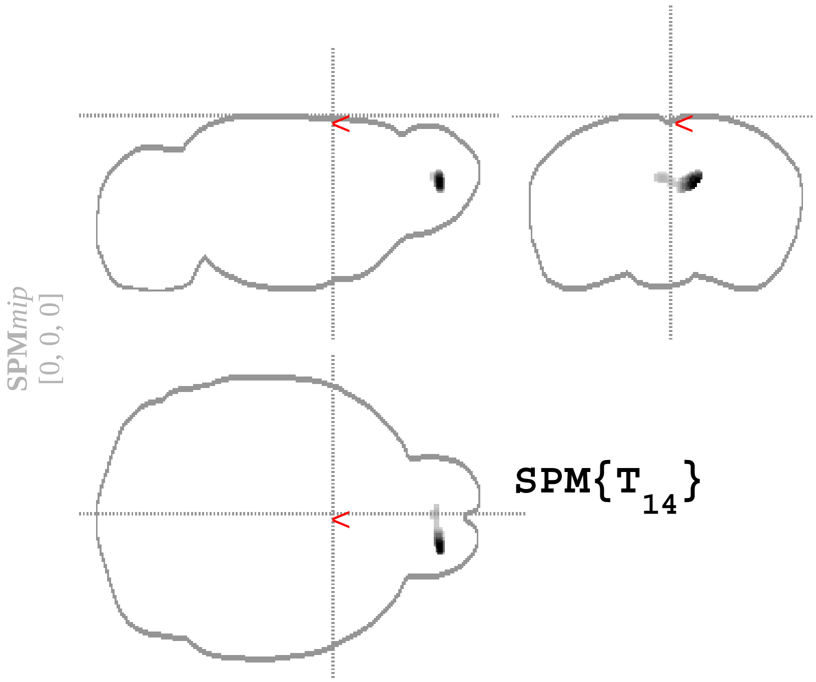

SPMresults: \Gray\result_KO-WT
Height threshold T = 2.624494 {p<0.01 (unc.)}
Extent threshold k = 50 voxels

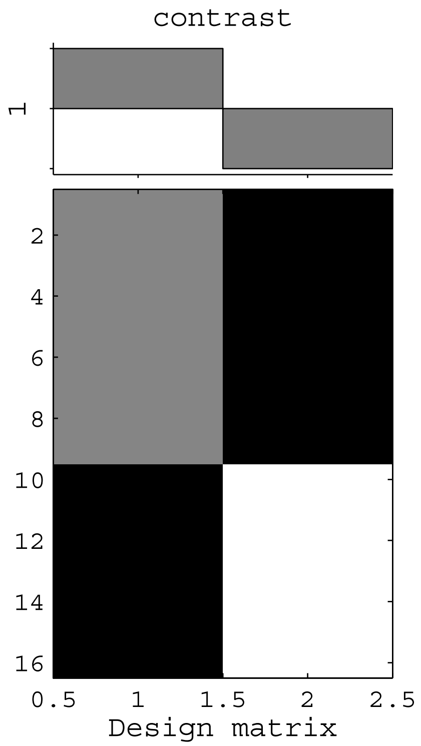

Statistics: ***p-values adjusted for search volume***

	cluster-level				peak-level					mm mm mm		
	$p_{FWE-corr}$	$q_{FDR-corr}$	k_E	p_{uncorr}	$p_{FWE-corr}$	$q_{FDR-corr}$	T	$(Z_{\equiv})$	p_{uncorr}			
	**0.994**	**0.952**	**118**	**0.743**	**0.878**	**0.890**	**3.65**	**3.01**	**0.001**	**-1**	**3**	**6**
					0.997	0.890	2.81	2.46	0.007	0	3	6

table shows 3 local maxima more than 8.0mm apart

Height threshold: T = 2.62, p = 0.010 (0.999) Degrees of freedom = [1.0, 14.0]
Extent threshold: k = 50 voxels, p = 0.846 (0.997) FWHM = 20.5 12.5 17.8 mm mm mm; 20.5 12.5 17.8 {vo
Expected voxels per cluster, <k> = 971.951 Volume: 377492 = 377492 voxels = 78.1 resels
Expected number of clusters, <c> = 5.81 Voxel size: 1.0 1.0 1.0 mm mm mm; (resel = 4551.86
FWEp: 6.527, FDRp: Inf, FWEc: Inf, FDRc: Inf

WT-KO

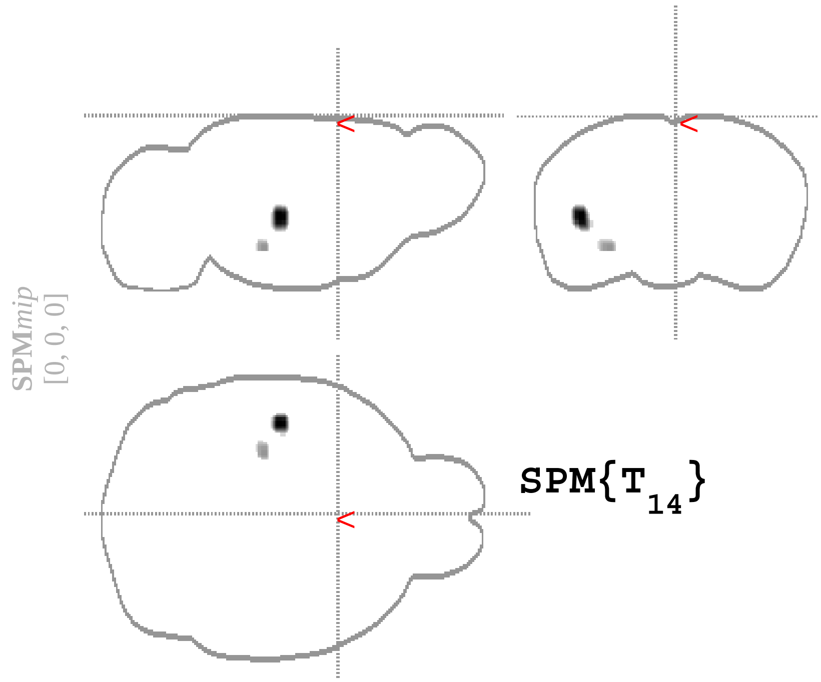

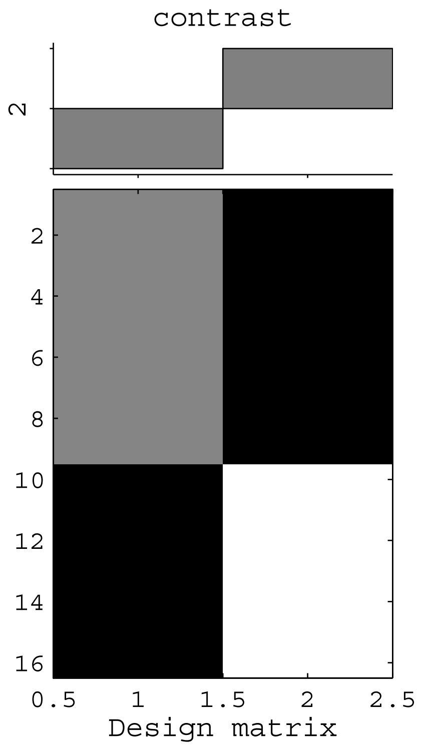

SPMresults: \Stay\result_KO-WT
Height threshold T = 2.624494 {p<0.01 (unc.)}
Extent threshold k = 50 voxels

Statistics: *p-values adjusted for search volume*

set-level		cluster-level				peak-level					mm mm mm
p	c	$p_{FWE-corr}$	$q_{FDR-corr}$	k_E	p_{uncorr}	$p_{FWE-corr}$	$q_{FDR-corr}$	T	$(Z_{\equiv})$	p_{uncorr}	
0.980	2	0.984	0.988	263	0.603	0.934	0.966	3.44	2.88	0.002	4 4 -2
		0.997	0.988	53	0.840	0.995	0.966	2.89	2.51	0.006	3 5 -3

table shows 3 local maxima more than 8.0mm apart

Height threshold: T = 2.62, p = 0.010 (0.999)
Degrees of freedom = [1.0, 14.0]
Extent threshold: k = 50 voxels, p = 0.846 (0.997)
FWHM = 20.5 12.5 17.8 mm mm mm; 20.5 12.5 17.8 {vo
Expected voxels per cluster, <k> = 971.951
Volume: 377492 = 377492 voxels = 78.1 resels
Expected number of clusters, <c> = 5.81
Voxel size: 1.0 1.0 1.0 mm mm mm; (resel = 4551.86
FWEp: 6.527, FDRp: Inf, FWEc: Inf, FDRc: Inf

KO-WT

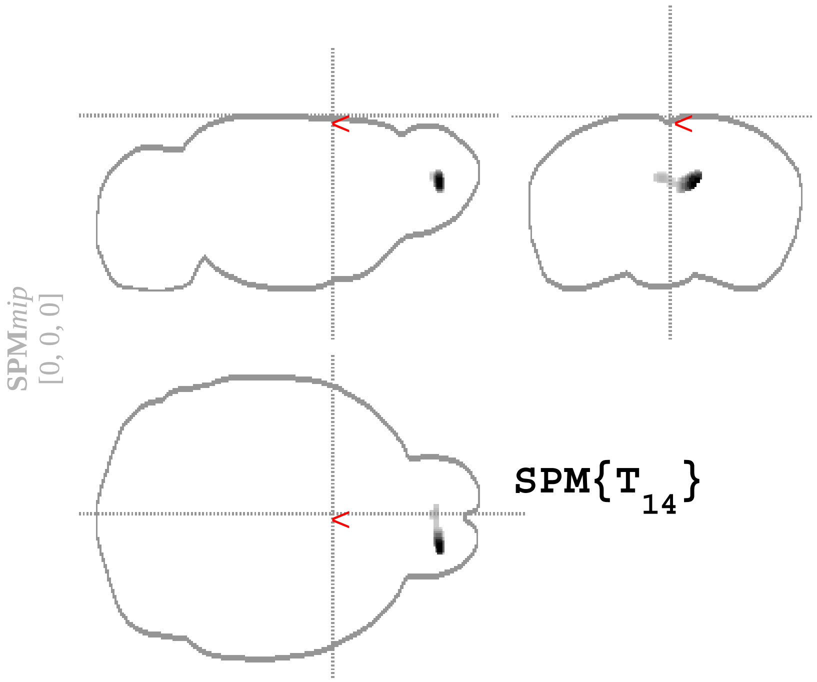

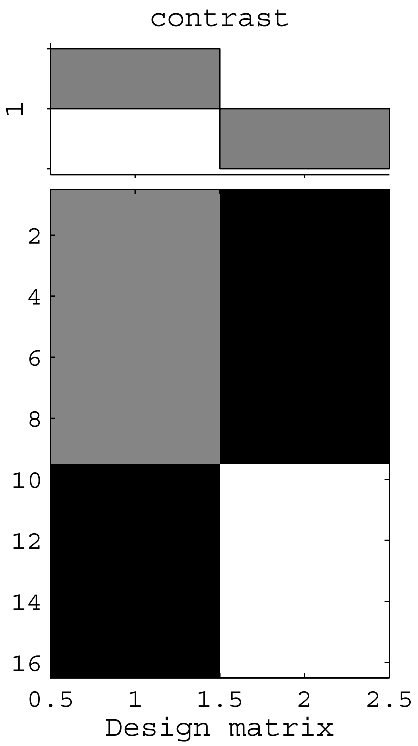

SPMresults: [illegible]ay\result_KO-WT
Height threshold T = 2.624494 {p<0.01 (unc.)}
Extent threshold k = 20 voxels

Statistics: *p-values adjusted for search volume*

	cluster-level				peak-level							
	$p_{FWE-corr}$	$q_{FDR-corr}$	k_E	p_{uncorr}	$p_{FWE-corr}$	$q_{FDR-corr}$	T	$(Z_{\equiv})$	p_{uncorr}	mm	mm	mm
	**0.994**	**0.952**	**118**	**0.743**	**0.878**	**0.890**	**3.65**	**3.01**	**0.001**	**-1**	**3**	**6**
					0.997	0.890	2.81	2.46	0.007	0	3	6

table shows 3 local maxima more than 8.0mm apart

Height threshold: T = 2.62, p = 0.010 (0.999)
Extent threshold: k = 20 voxels, p = 0.913 (0.998)
Expected voxels per cluster, <k> = 971.951
Expected number of clusters, <c> = 6.27
FWEp: 6.527, FDRp: Inf, FWEc: Inf, FDRc: Inf

Degrees of freedom = [1.0, 14.0]
FWHM = 20.5 12.5 17.8 mm mm mm; 20.5 12.5 17.8 {vo[illegible]
Volume: 377492 = 377492 voxels = 78.1 resels
Voxel size: 1.0 1.0 1.0 mm mm mm; (resel = 4551.86

WT-KO

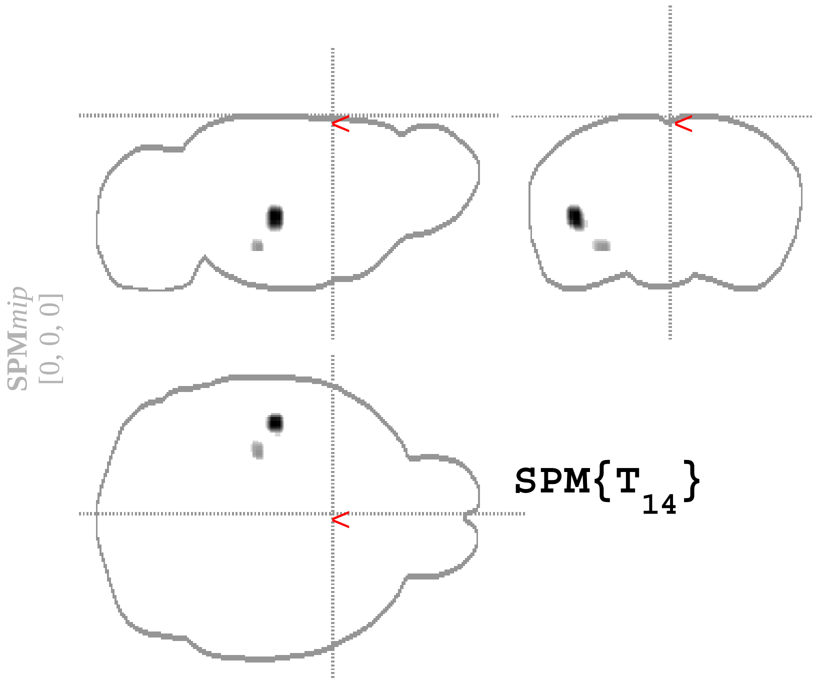

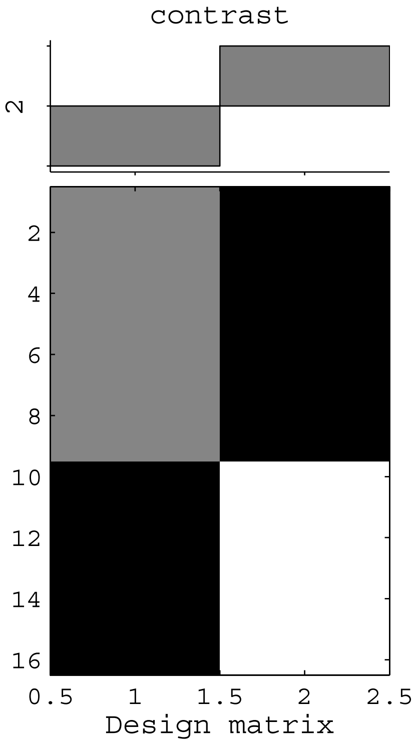

SPMresults: \Gray\result_KO-WT
Height threshold T = 2.624494 {p<0.01 (unc.)}
Extent threshold k = 20 voxels

Statistics: ***p-values adjusted for search volume***

set-level		cluster-level				peak-level							
p	c	$p_{FWE-corr}$	$q_{FDR-corr}$	k_E	p_{uncorr}	$p_{FWE-corr}$	$q_{FDR-corr}$	T	$(Z_{\equiv})$	p_{uncorr}	mm	mm	mm
0.986	2	0.984	0.988	263	0.603	0.934	0.966	3.44	2.88	0.002	4	4	-2
		0.997	0.988	53	0.840	0.995	0.966	2.89	2.51	0.006	3	5	-3

table shows 3 local maxima more than 8.0mm apart

Height threshold: T = 2.62, p = 0.010 (0.998)
Extent threshold: k = 20 voxels, p = 0.913 (0.998)
Expected voxels per cluster, <k> = 971.951
Expected number of clusters, <c> = 6.27
FWEp: 6.527, FDRp: Inf, FWEc: Inf, FDRc: Inf

Degrees of freedom = [1.0, 14.0]
FWHM = 20.5 12.5 17.8 mm mm mm; 20.5 12.5 17.8 {vo
Volume: 377492 = 377492 voxels = 78.1 resels
Voxel size: 1.0 1.0 1.0 mm mm mm; (resel = 4551.86

KO-WT

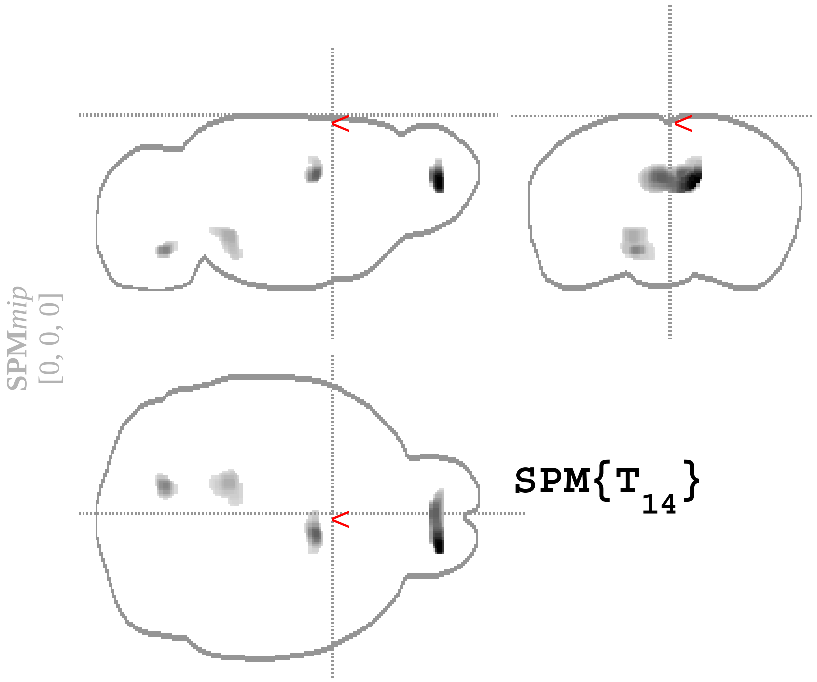

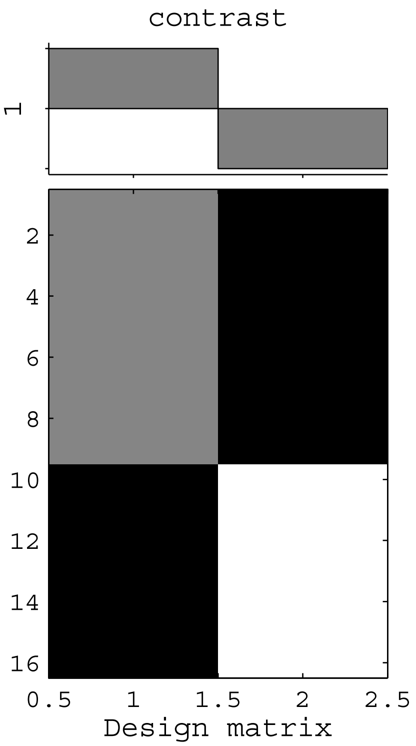

SPMresults:\Gray\result_KO-WT
Height threshold T = 1.761310 {p<0.05 (unc.)}
Extent threshold k = 100 voxels

Statistics: *p-values adjusted for search volume*

set-level		cluster-level				peak-level							
p	*c*	$p_{FWE-corr}$	$q_{FDR-corr}$	k_E	p_{uncorr}	$p_{FWE-corr}$	$q_{FDR-corr}$	*T*	$(Z_{\equiv})$	p_{uncorr}	mm	mm	mm
**0.997**	**4**	**1.000**	**0.992**	**727**	**0.669**	**0.878**	**0.991**	**3.65**	**3.01**	**0.001**	**-1**	**3**	**6**
						0.997	0.991	2.81	2.46	0.007	0	3	6
		**1.000**	**0.992**	**317**	**0.794**	**0.997**	**0.991**	**2.81**	**2.46**	**0.007**	**-1**	**2**	**-0**
		**1.000**	**0.992**	**162**	**0.863**	**1.000**	**0.991**	**2.50**	**2.23**	**0.013**	**1**	**5**	**-8**
		**1.000**	**0.992**	**622**	**0.696**	**1.000**	**0.991**	**2.18**	**2.00**	**0.023**	**1**	**5**	**-5**

table shows 3 local maxima more than 8.0mm apart

Height threshold: T = 1.76, p = 0.049 (1.000) Degrees of freedom = [1.0, 14.0]
Extent threshold: k = 100 voxels, p = 0.899 (1.000) FWHM = 20.5 12.5 17.8 mm mm mm; 20.5 12.5 17.8 {vo
Expected voxels per cluster, <k> = 3800.985 Volume: 377492 = 377492 voxels = 78.1 resels
Expected number of clusters, <c> = 11.80 Voxel size: 1.0 1.0 1.0 mm mm mm; (resel = 4551.86
FWEp: 6.527, FDRp: Inf, FWEc: Inf, FDRc: Inf

WT-KO

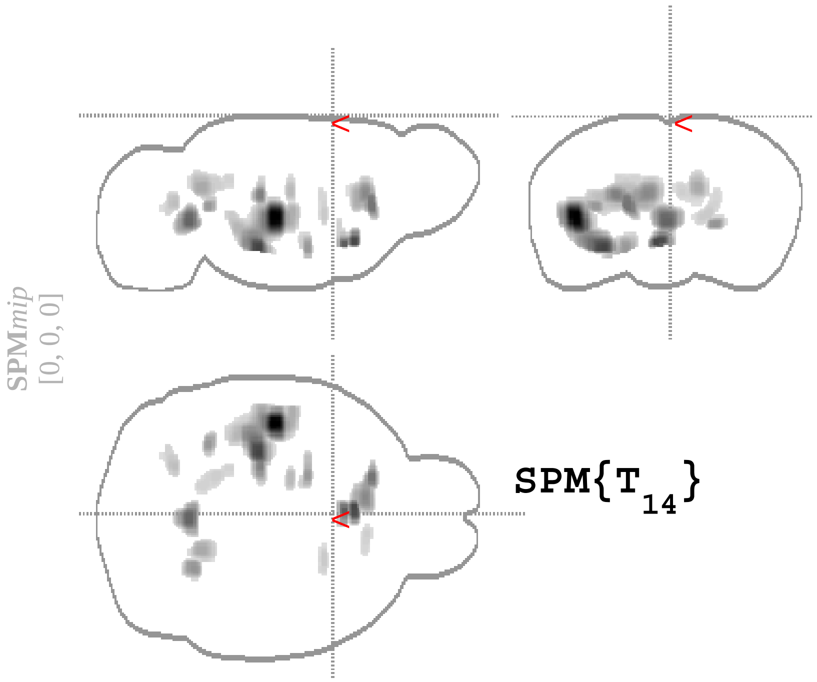

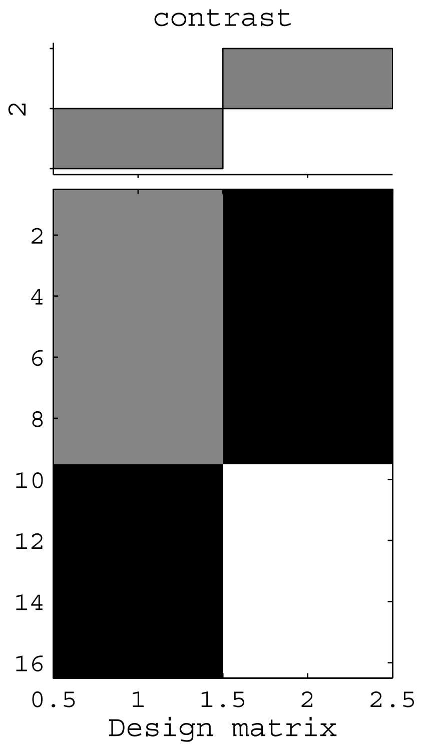

SPMresults: \Bay\result_KO-WT
Height threshold T = 1.761310 {p<0.05 (unc.)}
Extent threshold k = 100 voxels

Statistics: ***p-values adjusted for search volume***

set-level		cluster-level				peak-level					mm	mm	mm
p	c	$p_{FWE-corr}$	$q_{FDR-corr}$	k_E	p_{uncorr}	$p_{FWE-corr}$	$q_{FDR-corr}$	T	$(Z_{\equiv})$	p_{uncorr}			
0.401	13	0.966	0.992	4524	0.257	0.934	0.994	3.44	2.88	0.002	4	4	-2
						0.995	0.994	2.89	2.51	0.006	3	5	-3
						1.000	0.994	2.44	2.19	0.014	2	3	-3
		1.000	0.992	314	0.795	0.995	0.994	2.86	2.49	0.006	0	6	2
						1.000	0.994	1.86	1.74	0.041	0	5	1
		1.000	0.992	734	0.668	0.999	0.994	2.67	2.36	0.009	0	4	-7
		0.999	0.992	1163	0.578	0.999	0.994	2.55	2.28	0.011	2	4	2
						1.000	0.994	2.38	2.15	0.016	1	3	2
		1.000	0.992	166	0.861	1.000	0.994	2.31	2.09	0.018	-2	4	-7
		1.000	0.992	189	0.849	1.000	0.994	2.28	2.07	0.019	2	6	-1
		1.000	0.992	122	0.885	1.000	0.994	2.28	2.07	0.019	3	3	-6
		1.000	0.992	508	0.729	1.000	0.994	2.17	1.98	0.024	-1	2	-6
		1.000	0.992	192	0.848	1.000	0.994	2.05	1.89	0.030	2	3	-2
		1.000	0.992	203	0.842	1.000	0.994	2.00	1.85	0.032	2	3	-8
						1.000	0.994	1.86	1.74	0.041	3	3	-8
		1.000	0.992	110	0.892	1.000	0.994	1.93	1.79	0.037	-2	4	0
						1.000	0.994	1.83	1.71	0.044	-2	3	-0
		1.000	0.992	313	0.795	1.000	0.994	1.90	1.77	0.038	2	2	-5
		1.000	0.992	155	0.867	1.000	0.994	1.88	1.76	0.040	-1	3	2

table shows 3 local maxima more than 8.0mm apart

Height threshold: T = 1.76, p = 0.049 (1.000) Degrees of freedom = [1.0, 14.0]
Extent threshold: k = 100 voxels, p = 0.899 (1.000) FWHM = 20.5 12.5 17.8 mm mm mm; 20.5 12.5 17.8 {vo
Expected voxels per cluster, <k> = 3800.985 Volume: 377492 = 377492 voxels = 78.1 resels
Expected number of clusters, <c> = 11.80 Voxel size: 1.0 1.0 1.0 mm mm mm; (resel = 4551.86
FWEp: 6.527, FDRp: Inf, FWEc: Inf, FDRc: Inf

KO-WT

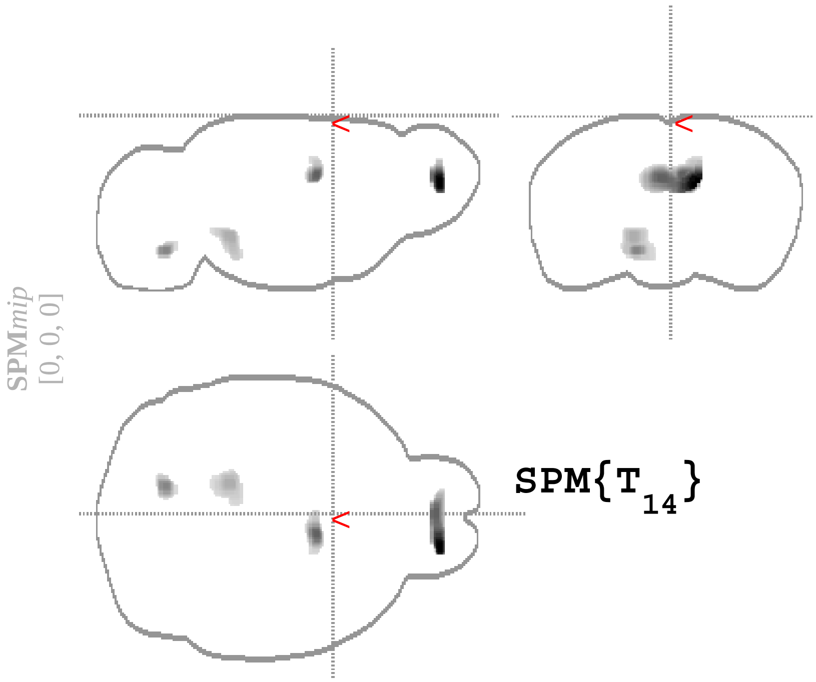

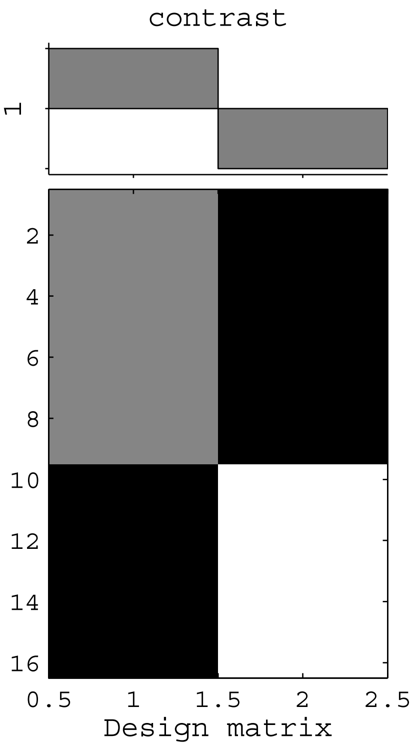

SPMresults: \Gray\result_KO-WT
Height threshold T = 1.761310 {p<0.05 (unc.)}
Extent threshold k = 50 voxels

Statistics: ***p-values adjusted for search volume***

set-level		cluster-level				peak-level					mm	mm	mm
p	c	$p_{FWE-corr}$	$q_{FDR-corr}$	k_E	p_{uncorr}	$p_{FWE-corr}$	$q_{FDR-corr}$	T	$(Z_{\equiv})$	p_{uncorr}			
**0.998**	**4**	**1.000**	**0.992**	**727**	**0.669**	**0.878**	**0.991**	**3.65**	**3.01**	**0.001**	**-1**	**3**	**6**
						0.997	0.991	2.81	2.46	0.007	0	3	6
		**1.000**	**0.992**	**317**	**0.794**	**0.997**	**0.991**	**2.81**	**2.46**	**0.007**	**-1**	**2**	**-0**
		**1.000**	**0.992**	**162**	**0.863**	**1.000**	**0.991**	**2.50**	**2.23**	**0.013**	**1**	**5**	**-8**
		**1.000**	**0.992**	**622**	**0.696**	**1.000**	**0.991**	**2.18**	**2.00**	**0.023**	**1**	**5**	**-5**

table shows 3 local maxima more than 8.0mm apart

Height threshold: T = 1.76, p = 0.049 (1.000) Degrees of freedom = [1.0, 14.0]
Extent threshold: k = 50 voxels, p = 0.935 (1.000) FWHM = 20.5 12.5 17.8 mm mm mm; 20.5 12.5 17.8 {vo
Expected voxels per cluster, <k> = 3800.985 Volume: 377492 = 377492 voxels = 78.1 resels
Expected number of clusters, <c> = 12.27 Voxel size: 1.0 1.0 1.0 mm mm mm; (resel = 4551.86
FWEp: 6.527, FDRp: Inf, FWEc: Inf, FDRc: Inf

WT-KO

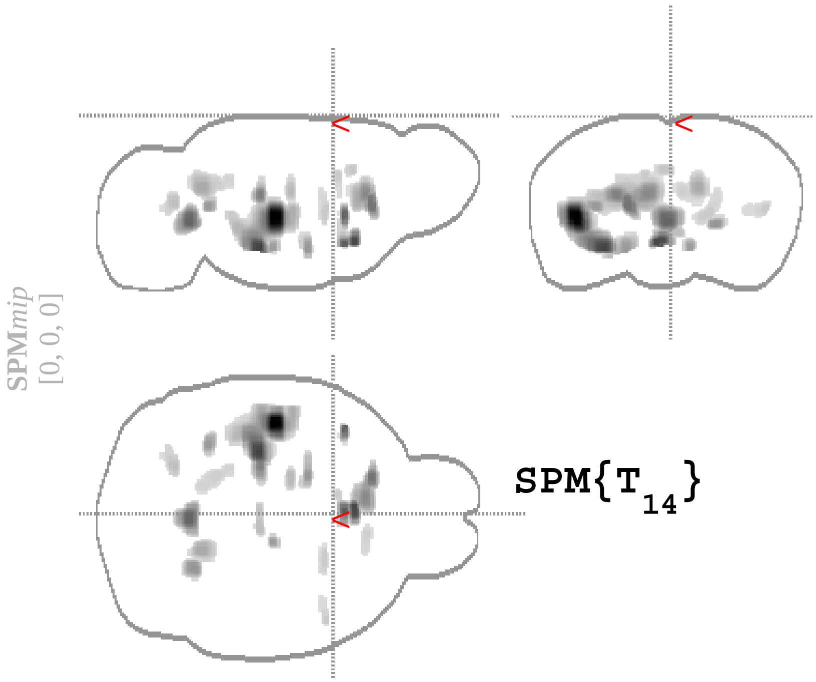

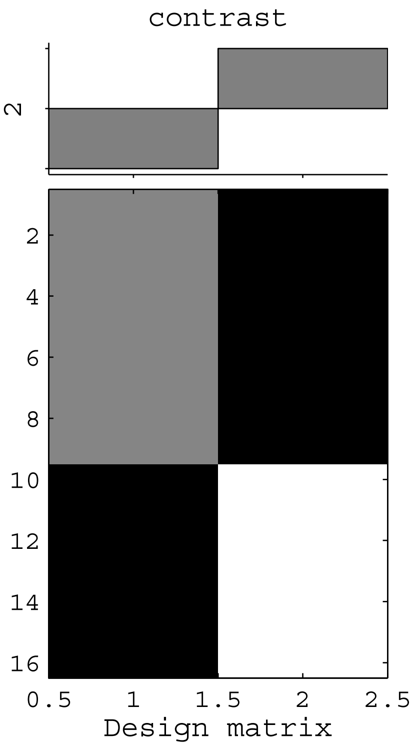

SPMresults: \Ray\result_KO-WT
Height threshold T = 1.761310 {p<0.05 (unc.)}
Extent threshold k = 50 voxels

Statistics: *p-values adjusted for search volume*

set-level		cluster-level				peak-level					mm	mm	mm
p	c	$p_{FWE-corr}$	$q_{FDR-corr}$	k_E	p_{uncorr}	$p_{FWE-corr}$	$q_{FDR-corr}$	T	$(Z_\equiv)$	p_{uncorr}			
0.045	19	0.966	0.992	4524	0.257	0.934	0.994	3.44	2.88	0.002	4	4	-2
						0.995	0.994	2.89	2.51	0.006	3	5	-3
						1.000	0.994	2.44	2.19	0.014	2	3	-3
		1.000	0.992	314	0.795	0.995	0.994	2.86	2.49	0.006	0	6	2
						1.000	0.994	1.86	1.74	0.041	0	5	1
		1.000	0.992	734	0.668	0.999	0.994	2.67	2.36	0.009	0	4	-7
		1.000	0.992	78	0.913	0.999	0.994	2.66	2.35	0.009	4	4	1
		0.999	0.992	1163	0.578	0.999	0.994	2.55	2.28	0.011	2	4	2
						1.000	0.994	2.38	2.15	0.016	1	3	2
		1.000	0.992	166	0.861	1.000	0.994	2.31	2.09	0.018	-2	4	-7
		1.000	0.992	189	0.849	1.000	0.994	2.28	2.07	0.019	2	6	-1
		1.000	0.992	122	0.885	1.000	0.994	2.28	2.07	0.019	3	3	-6
		1.000	0.992	56	0.930	1.000	0.994	2.24	2.04	0.021	-1	6	-3
		1.000	0.992	508	0.729	1.000	0.994	2.17	1.98	0.024	-1	2	-6
		1.000	0.992	192	0.848	1.000	0.994	2.05	1.89	0.030	2	3	-2
		1.000	0.992	73	0.917	1.000	0.994	2.02	1.87	0.031	-0	5	-3
						1.000	0.994	1.99	1.84	0.033	0	5	-3
		1.000	0.992	203	0.842	1.000	0.994	2.00	1.85	0.032	2	3	-8
						1.000	0.994	1.86	1.74	0.041	3	3	-8
		1.000	0.992	72	0.918	1.000	0.994	1.96	1.82	0.035	0	2	1
		1.000	0.992	110	0.892	1.000	0.994	1.93	1.79	0.037	-2	4	0
						1.000	0.994	1.83	1.71	0.044	-2	3	-0

table shows 3 local maxima more than 8.0mm apart

Height threshold: T = 1.76, p = 0.049 (1.000) Degrees of freedom = [1.0, 14.0]
Extent threshold: k = 50 voxels, p = 0.935 (1.000) FWHM = 20.5 12.5 17.8 mm mm mm; 20.5 12.5 17.8 {vo
Expected voxels per cluster, <k> = 3800.985 Volume: 377492 = 377492 voxels = 78.1 resels
Expected number of clusters, <c> = 12.27 Voxel size: 1.0 1.0 1.0 mm mm mm; (resel = 4551.86
FWEp: 6.527, FDRp: Inf, FWEc: Inf, FDRc: Inf

WT-KO

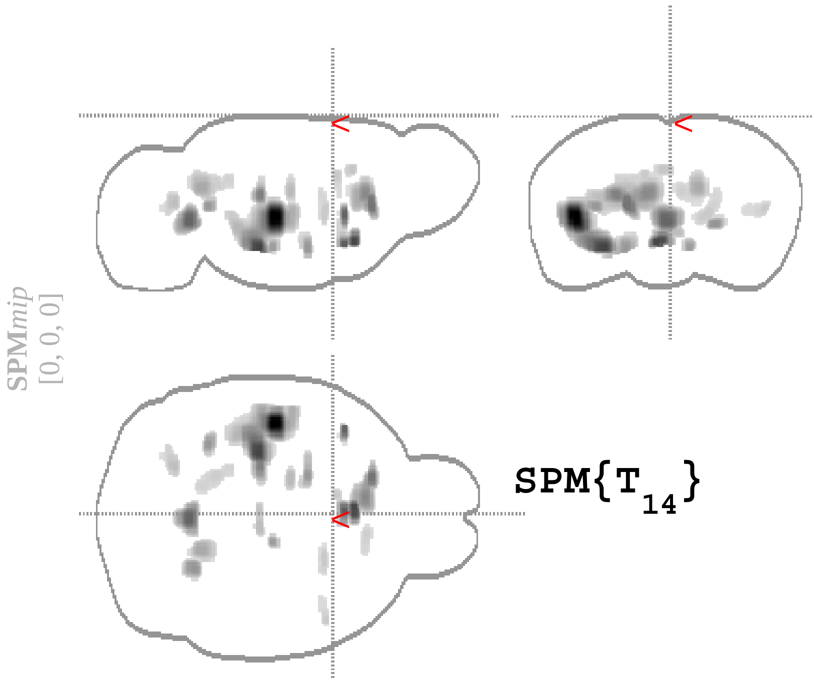

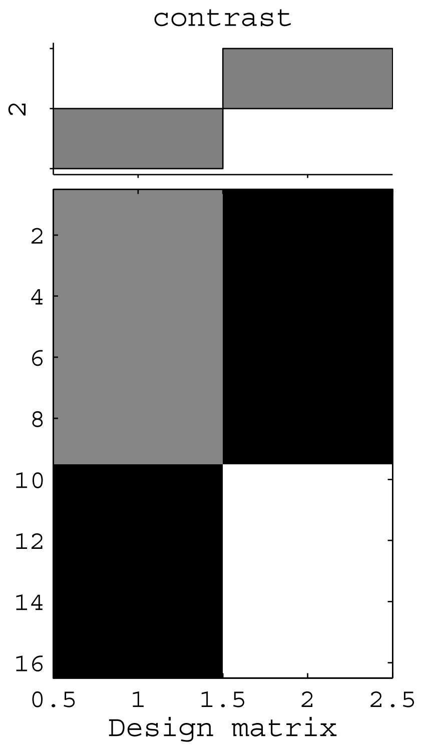

SPMresults: \Gray\result_KO-WT
Height threshold T = 1.761310 {p<0.05 (unc.)}
Extent threshold k = 50 voxels

Statistics: ***p-values adjusted for search volume***

set-level p	set-level c	cluster-level $p_{FWE-corr}$	$q_{FDR-corr}$	k_E	p_{uncorr}	peak-level $p_{FWE-corr}$	$q_{FDR-corr}$	T	$(Z_{\equiv})$	p_{uncorr}	mm	mm	mm
		1.000	0.992	81	0.911	1.000	0.994	1.91	1.78	0.037	-4	4	0
		1.000	0.992	75	0.916	1.000	0.994	1.91	1.78	0.038	1	2	1
		1.000	0.992	313	0.795	1.000	0.994	1.90	1.77	0.038	2	2	-5
		1.000	0.992	155	0.867	1.000	0.994	1.88	1.76	0.040	-1	3	2

table shows 3 local maxima more than 8.0mm apart

Height threshold: T = 1.76, p = 0.049 (1.000) Degrees of freedom = [1.0, 14.0]
Extent threshold: k = 50 voxels, p = 0.935 (1.000) FWHM = 20.5 12.5 17.8 mm mm mm; 20.5 12.5 17.8 {vo
Expected voxels per cluster, <k> = 3800.985 Volume: 377492 = 377492 voxels = 78.1 resels
Expected number of clusters, <c> = 12.27 Voxel size: 1.0 1.0 1.0 mm mm mm; (resel = 4551.86
FWEp: 6.527, FDRp: Inf, FWEc: Inf, FDRc: Inf

KO-WT

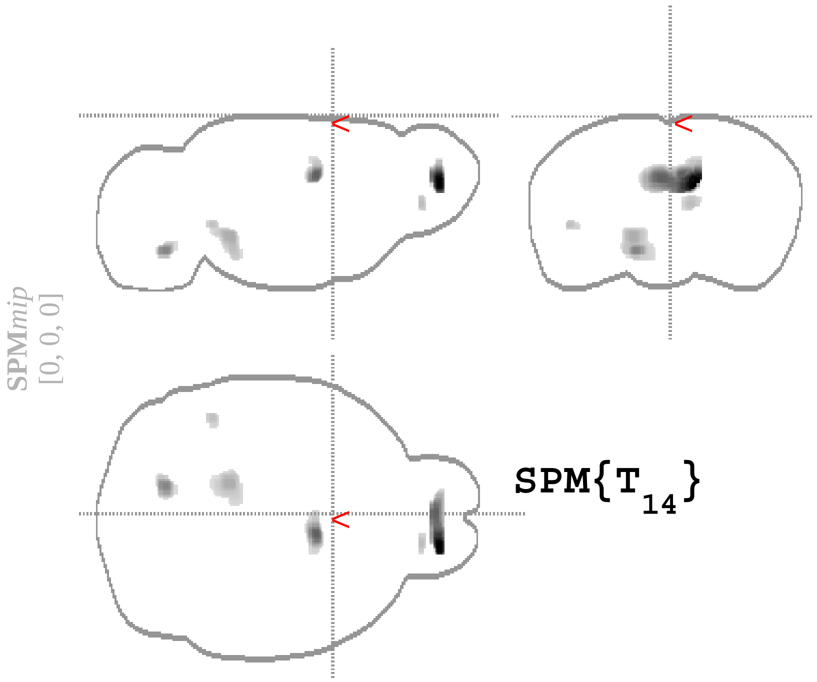

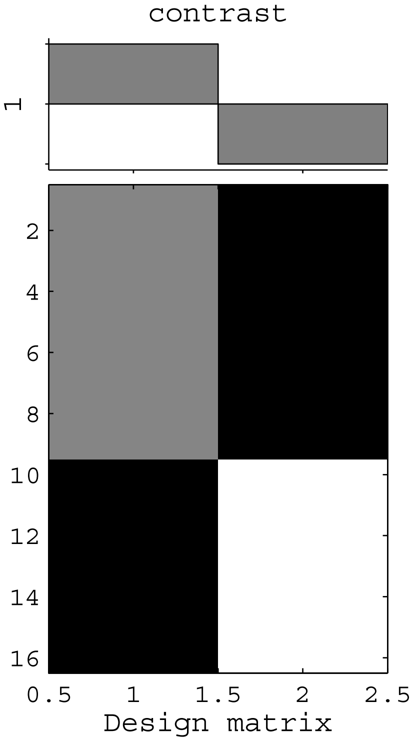

SPMresults: \Gray\result_KO-WT
Height threshold T = 1.761310 {p<0.05 (unc.)}
Extent threshold k = 20 voxels

Statistics: ***p-values adjusted for search volume***

set-level		cluster-level				peak-level							
p	c	$p_{FWE-corr}$	$q_{FDR-corr}$	k_E	p_{uncorr}	$p_{FWE-corr}$	$q_{FDR-corr}$	T	$(Z_{\equiv})$	p_{uncorr}	mm	mm	mm
0.987	6	1.000	0.992	727	0.669	0.878	0.991	3.65	3.01	0.001	-1	3	6
						0.997	0.991	2.81	2.46	0.007	0	3	6
		1.000	0.992	317	0.794	0.997	0.991	2.81	2.46	0.007	-1	2	-0
		1.000	0.992	162	0.863	1.000	0.991	2.50	2.23	0.013	1	5	-8
		1.000	0.992	622	0.696	1.000	0.991	2.18	2.00	0.023	1	5	-5
		1.000	0.992	36	0.947	1.000	0.991	2.03	1.87	0.031	-1	4	5
		1.000	0.992	30	0.953	1.000	0.991	2.00	1.85	0.032	4	4	-6

table shows 3 local maxima more than 8.0mm apart

Height threshold: T = 1.76, p = 0.049 (1.000)
Extent threshold: k = 20 voxels, p = 0.964 (1.000)
Expected voxels per cluster, <k> = 3800.985
Expected number of clusters, <c> = 12.66
FWEp: 6.527, FDRp: Inf, FWEc: Inf, FDRc: Inf

Degrees of freedom = [1.0, 14.0]
FWHM = 20.5 12.5 17.8 mm mm mm; 20.5 12.5 17.8 {vo
Volume: 377492 = 377492 voxels = 78.1 resels
Voxel size: 1.0 1.0 1.0 mm mm mm; (resel = 4551.86

WT-KO

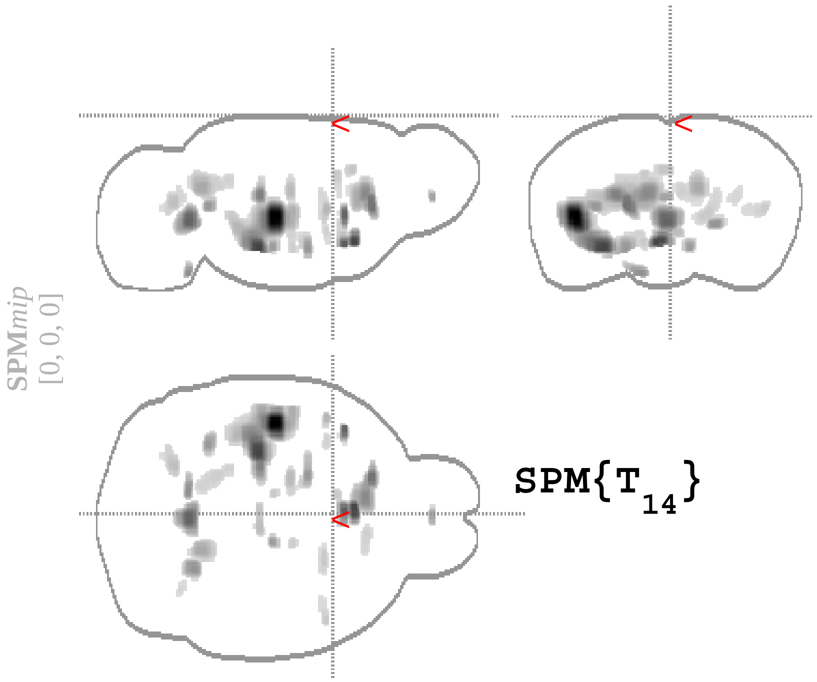

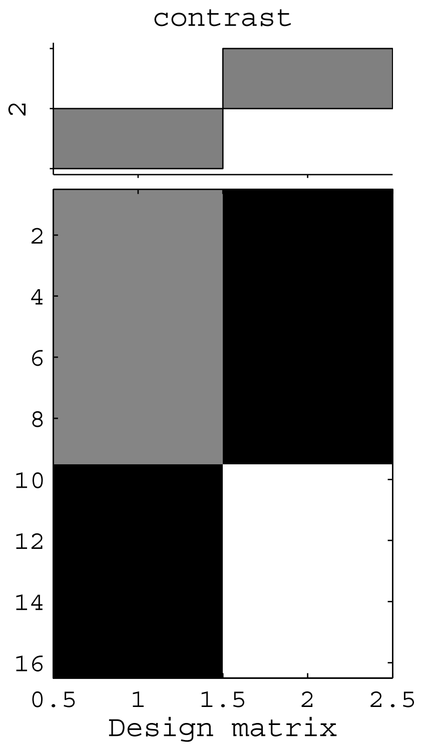

SPMresults: \Gray\result_KO-WT
Height threshold T = 1.761310 {p<0.05 (unc.)}
Extent threshold k = 20 voxels

Statistics: *p-values adjusted for search volume*

set-level		cluster-level				peak-level							
p	c	$p_{FWE-corr}$	$q_{FDR-corr}$	k_E	p_{uncorr}	$p_{FWE-corr}$	$q_{FDR-corr}$	T	$(Z_\equiv)$	p_{uncorr}	mm	mm	mm
**0.000**	**27**	**0.966**	**0.992**	**4524**	**0.257**	**0.934**	**0.994**	**3.44**	**2.88**	**0.002**	**4**	**4**	**-2**
						0.995	0.994	2.89	2.51	0.006	3	5	-3
						1.000	0.994	2.44	2.19	0.014	2	3	-3
		**1.000**	**0.992**	**314**	**0.795**	**0.995**	**0.994**	**2.86**	**2.49**	**0.006**	**0**	**6**	**2**
						1.000	0.994	1.86	1.74	0.041	0	5	1
		**1.000**	**0.992**	**734**	**0.668**	**0.999**	**0.994**	**2.67**	**2.36**	**0.009**	**0**	**4**	**-7**
		**1.000**	**0.992**	**78**	**0.913**	**0.999**	**0.994**	**2.66**	**2.35**	**0.009**	**4**	**4**	**1**
		**0.999**	**0.992**	**1163**	**0.578**	**0.999**	**0.994**	**2.55**	**2.28**	**0.011**	**2**	**4**	**2**
						1.000	0.994	2.38	2.15	0.016	1	3	2
		**1.000**	**0.992**	**44**	**0.940**	**1.000**	**0.994**	**2.34**	**2.11**	**0.017**	**1**	**6**	**-7**
		**1.000**	**0.992**	**166**	**0.861**	**1.000**	**0.994**	**2.31**	**2.09**	**0.018**	**-2**	**4**	**-7**
		**1.000**	**0.992**	**189**	**0.849**	**1.000**	**0.994**	**2.28**	**2.07**	**0.019**	**2**	**6**	**-1**
		**1.000**	**0.992**	**122**	**0.885**	**1.000**	**0.994**	**2.28**	**2.07**	**0.019**	**3**	**3**	**-6**
		**1.000**	**0.992**	**56**	**0.930**	**1.000**	**0.994**	**2.24**	**2.04**	**0.021**	**-1**	**6**	**-3**
		**1.000**	**0.992**	**30**	**0.953**	**1.000**	**0.994**	**2.20**	**2.01**	**0.022**	**0**	**4**	**6**
		**1.000**	**0.992**	**508**	**0.729**	**1.000**	**0.994**	**2.17**	**1.98**	**0.024**	**-1**	**2**	**-6**
		**1.000**	**0.992**	**40**	**0.944**	**1.000**	**0.994**	**2.06**	**1.90**	**0.029**	**4**	**4**	**0**
		**1.000**	**0.992**	**192**	**0.848**	**1.000**	**0.994**	**2.05**	**1.89**	**0.030**	**2**	**3**	**-2**
		**1.000**	**0.992**	**73**	**0.917**	**1.000**	**0.994**	**2.02**	**1.87**	**0.031**	**-0**	**5**	**-3**
						1.000	0.994	1.99	1.84	0.033	0	5	-3
		**1.000**	**0.992**	**203**	**0.842**	**1.000**	**0.994**	**2.00**	**1.85**	**0.032**	**2**	**3**	**-8**
						1.000	0.994	1.86	1.74	0.041	3	3	-8

table shows 3 local maxima more than 8.0mm apart

Height threshold: T = 1.76, p = 0.049 (1.000) Degrees of freedom = [1.0, 14.0]
Extent threshold: k = 20 voxels, p = 0.964 (1.000) FWHM = 20.5 12.5 17.8 mm mm mm; 20.5 12.5 17.8 {vo
Expected voxels per cluster, <k> = 3800.985 Volume: 377492 = 377492 voxels = 78.1 resels
Expected number of clusters, <c> = 12.66 Voxel size: 1.0 1.0 1.0 mm mm mm; (resel = 4551.86
FWEp: 6.527, FDRp: Inf, FWEc: Inf, FDRc: Inf

WT-KO

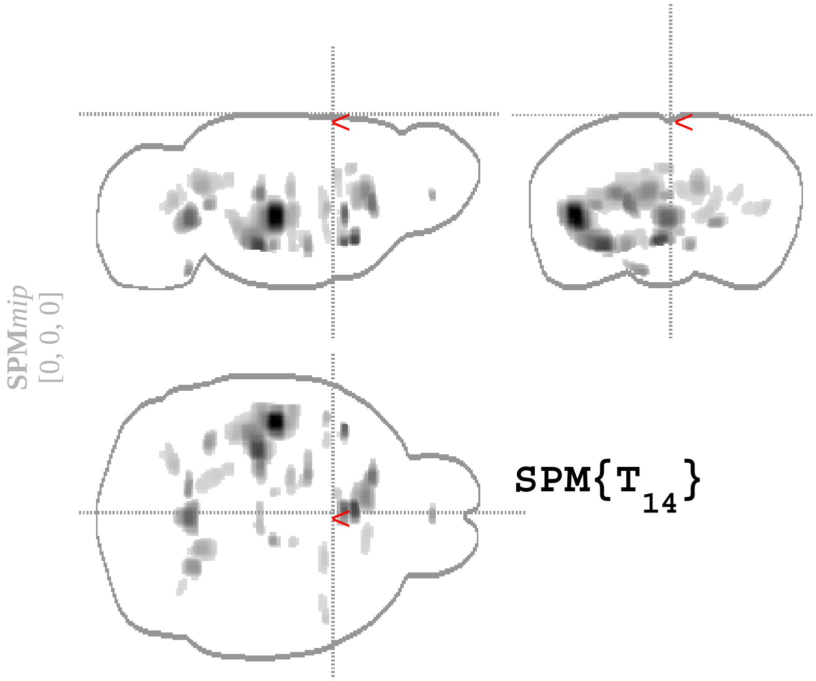

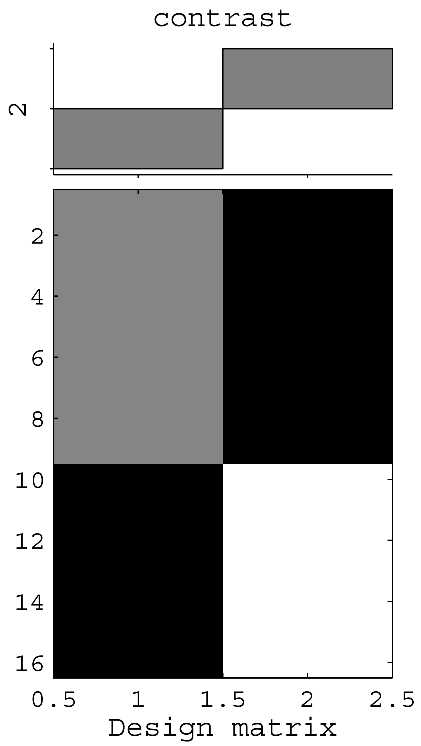

SPMresults: \Gray\result_KO-WT
Height threshold T = 1.761310 {p<0.05 (unc.)}
Extent threshold k = 20 voxels

Statistics: *p-values adjusted for search volume*

set-level		cluster-level				peak-level							
p	c	$p_{FWE-corr}$	$q_{FDR-corr}$	k_E	p_{uncorr}	$p_{FWE-corr}$	$q_{FDR-corr}$	T	$(Z_{\equiv})$	p_{uncorr}	mm	mm	mm
		1.000	0.992	72	0.918	1.000	0.994	1.96	1.82	0.035	0	2	1
		1.000	0.992	110	0.892	1.000	0.994	1.93	1.79	0.037	-2	4	0
						1.000	0.994	1.83	1.71	0.044	-2	3	-0
		1.000	0.992	81	0.911	1.000	0.994	1.91	1.78	0.037	-4	4	0
		1.000	0.992	75	0.916	1.000	0.994	1.91	1.78	0.038	1	2	1
		1.000	0.992	27	0.956	1.000	0.994	1.91	1.78	0.038	1	5	-2
		1.000	0.992	313	0.795	1.000	0.994	1.90	1.77	0.038	2	2	-5
		1.000	0.992	32	0.951	1.000	0.994	1.90	1.77	0.038	-1	6	-2
		1.000	0.992	155	0.867	1.000	0.994	1.88	1.76	0.040	-1	3	2
		1.000	0.992	20	0.964	1.000	0.994	1.86	1.74	0.041	1	5	-2
		1.000	0.992	42	0.942	1.000	0.994	1.85	1.73	0.042	3	5	0
		1.000	0.992	32	0.951	1.000	0.994	1.85	1.73	0.042	-3	3	-7

table shows 3 local maxima more than 8.0mm apart

Height threshold: T = 1.76, p = 0.049 (1.000) Degrees of freedom = [1.0, 14.0]
Extent threshold: k = 20 voxels, p = 0.964 (1.000) FWHM = 20.5 12.5 17.8 mm mm mm; 20.5 12.5 17.8 {vo
Expected voxels per cluster, <k> = 3800.985 Volume: 377492 = 377492 voxels = 78.1 resels
Expected number of clusters, <c> = 12.66 Voxel size: 1.0 1.0 1.0 mm mm mm; (resel = 4551.86
FWEp: 6.527, FDRp: Inf, FWEc: Inf, FDRc: In
